# Are “GABAergic” Agents Really So Selective for GABA? Implications for Single- versus Multi-Site Hypotheses From Promiscuous Behavior of Anesthetics and Their Molecular Targets In Vitro

**DOI:** 10.1213/ANE.0000000000007888

**Published:** 2026-01-23

**Authors:** Kush M. Kale, Peter A. Goldstein, Jaideep J. Pandit

**Affiliations:** From the 1 Nuffield Department of Anaesthetics, Oxford University Hospitals NHS Foundation Trust, Oxford, UK; 2 Department of Anesthesiology, Weill Cornell Medicine, New York, New York; 3 Department of Medicine, Weill Cornell Medicine, New York, New York; 4 Feil Family Brain and Mind Research Institute, Weill Cornell Medicine, New York, New York; 5 Nuffield Department of Clinical Neurosciences, University of Oxford, Oxford, UK.

## Abstract

In the literature, anesthetic agents are commonly described as if their hypnotic action is mediated by a predominant molecular target, using terminology such as “GABAergic” or “NMDAergic.” The justification for this is unclear. The authors might thereby imply a single target hypothesis (ie, the named target is the sole or predominant molecular mechanism for that agent); or that in an in vitro dose-response plot, the potency or efficacy of this agent is distinctly (ie, several orders of magnitude) highest for interaction with the named target as compared with others. We explored if this last was the case, through review of data reported across 310 in vitro studies. We found that all but a few agents were highly “promiscuous,” influencing several different molecular targets with similar potency, making it difficult to justify that any one was predominant. Notably, propofol, often cited as a “typical GABAergic agent” in fact shows higher potency at nicotinic receptors and HCN ion channels than for GABA_A_ receptors (GABA_A_Rs). Exceptions to promiscuity were, to an extent, etomidate and dexmedetomidine, which were relatively “monogomous” for GABA_A_R and α2-adrenoreceptors, respectively, and ketamine for NMDA receptors. Moreover, molecular targets were also promiscuous for which agents they were influenced by. This was in a manner that did not always correspond to the “preference” of an agent for a receptor. We discuss how this mutual promiscuity is consistent with a multi-site mechanism of anesthetic action.

**See Article, page 1033**

The report by Franks and Lieb^1^ (and earlier Ueda^2^) that anesthetics could bind to a pure protein target led in turn to their seminal review^3^ which emphasized a relatively small number of molecular targets, notably GABA_A_ receptors (γ-aminobutyric acid type A receptors; GABA_A_Rs). Subsequently, a language has emerged in discussion of molecular mechanisms that agents exhibit specificity for molecular targets and therefore can be readily described as “GABAergic” (for propofol, etomidate, and the barbiturates), “NMDAergic” (for those acting on N-methyl-D-aspartate receptors, like ketamine), etc. These receptor-level effects are argued to translate to meso-level descriptions for agents, wherein certain agents show “GABAergic” EEG signatures, whereas others exhibit “NMDAergic” and so on.^4^ Examples of language commonly used are provided for illustration in Supplemental Digital Content S1, https://links.lww.com/AA/F642. The implied framework is perhaps best exemplified by Brown et al.,^5^ who sought explicitly to categorize anesthetic action by the predominant receptor type for each agent, emphasizing 3: GABA_A_R (for propofol, thiopentone and other agents); NMDAR (for ketamine), and α2 receptors (for dexmedetomidine).

It is not always clear what justifies this target-specific terminology. One possibility is that the authors using it imply a “unitary hypothesis”: that hypnosis arises for the given agent through action at the named target. Some example sentences in Supplemental Digital Content S1, https://links.lww.com/AA/F642 appear explicit in this regard. Even if instead this terminology is useful shorthand used by authors who in fact acknowledge that anesthetics act at multiple sites, the implied emphasis is that the named target is nevertheless “predominant” warrants justification. An alternative explanation is that the first study to unambiguously demonstrate a statistically significant effect on a given target (as with propofol for GABA_A_R) establishes the precedent for characterization of the agent.^6^ Yet we know that all conventionally used anesthetic agents classed as “GABAergic” bind readily to other molecular targets. A different possibility, and one often alluded to in the literature (see Supplemental Digital Content S1, https://links.lww.com/AA/F642) is that “GABAergic” refers to the concentration-response relationships for in vitro single known channel studies: the “GABAergic” agent displaying quantitatively higher potency or efficacy (the maximum effect), or both, for GABA_A_R vs other receptors that it binds to. Potency is the concentration achieving 50% of the maximum effect (EC_50_ if excitatory; IC_50_ if inhibitory).

In this report, we examined if this last possibility is in fact the case. By reviewing results of in vitro studies on molecular targets, we constructed a series of concentration-response curves to assess whether anesthetic agents can be readily characterized as acting on a predominant, or exclusive receptor type, through specific affinity, higher potency, or higher efficacy. Our results challenge the single target-focused terminology. Instead, we argue (after Eckenhoff^7^) that anesthetics are highly “promiscuous.” Most, if not all, agents commonly bind to several targets with far less selectivity than implied in common terminology. Moreover, these various targets are themselves promiscuous for several agents.

We discuss the implications of this mutual promiscuity for molecular theories of anesthetic action. We argue that anesthetic action at the clinically relevant behavioral end points does not arise from agents targeting *one* unique target over another, but by targeting a *spectrum* of targets, this *spectrum* being unique for each agent. This notion restates summaries already in some authoritative texts which stress that the effects of anesthetics “…cannot be explained by a single molecular mechanism. Rather multiple targets contribute to the component actions comprising anesthesia….” This is in contrast to the examples given in Supplemental Digital Content S1, https://links.lww.com/AA/F642.^8^

## RECONSTRUCTING IN VITRO “CONCENTRATION-RESPONSE” CURVES

**Figure 1.**
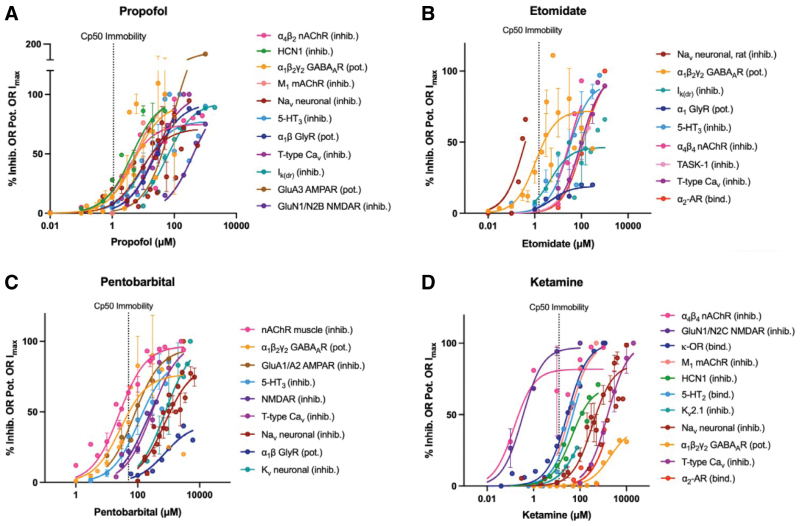
Concentration-response relationships for 4 intravenous anesthetics on various ligand receptors and ion channels. Individual data points represent mean (±SEM) effect averaged from different studies. The *y*-axis is the % inhibition (all values <100% maximum), % potentiation (eg, of a background current, as in TASK), or I_max_, where 100% I_max_ is the maximum response inducible by GABA, glycine, or 5-HT (and so where the agent could have effect >100% of this). Curves are fit to standard single receptor ligand binding. The vertical dashed line represents the approximate clinically relevant concentration for the endpoint (Cp50) of immobility. Note that some agents potentiate activity in a given target, whereas at other targets, the agent inhibits activity, which is denoted by: pot: potentiation and inhib: inhibition, respectively. The interaction of some agents with their targets was measured by binding affinity (bind); see detailed explanation in Supplemental Digital Content S2, https://links.lww.com/AA/F642.

**Figure 2.**
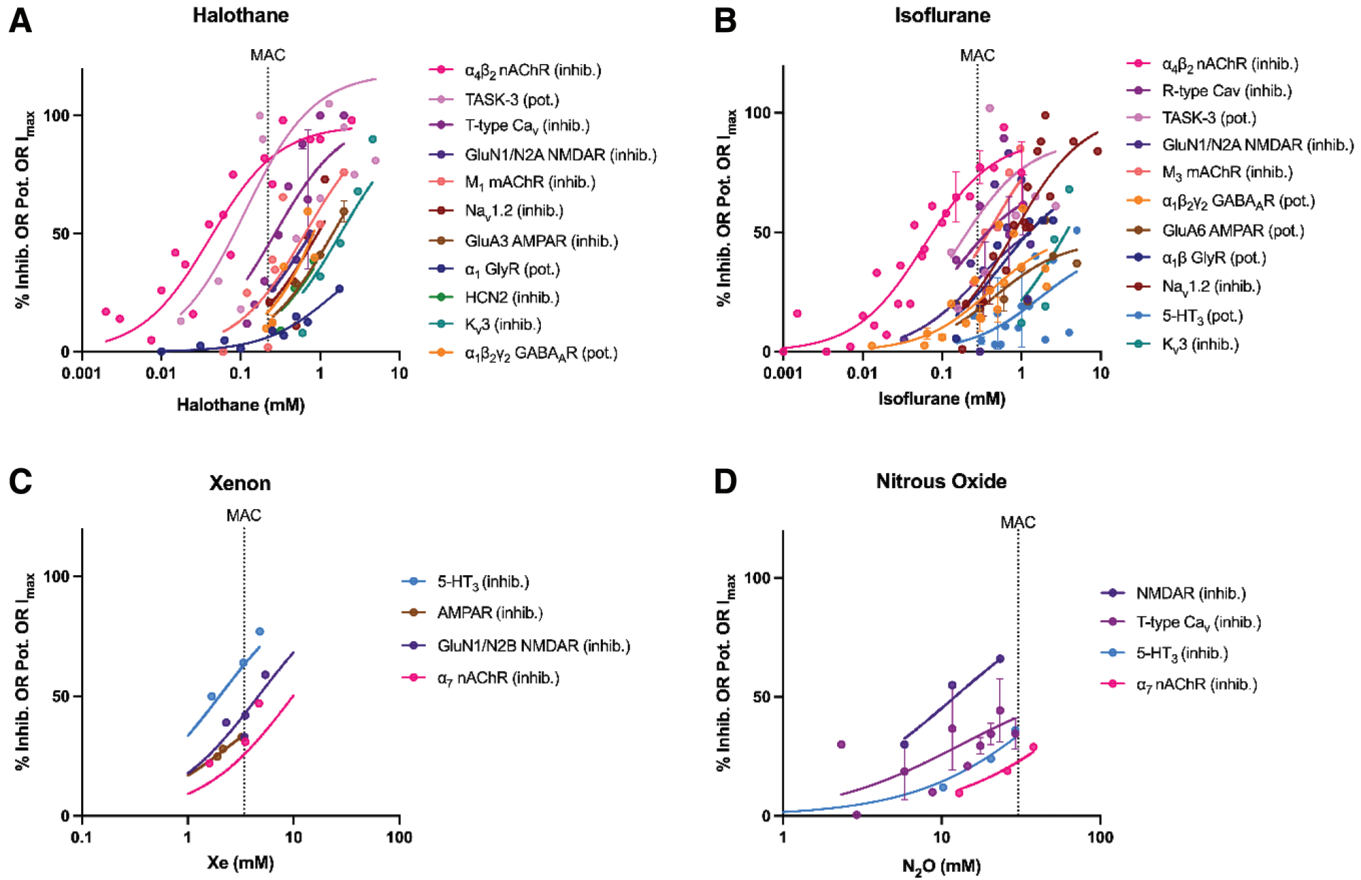
Concentration-response relationships for 4 inhaled anesthetics acting on various ligand receptors and ion channels. Individual data points represent mean (± SEM) effect averaged from different studies. The axes are as for Figure 1. Curves are fit to standard single receptor ligand binding. The vertical dashed line represents the approximate clinically relevant concentration for end point (MAC) of immobility. Note that some agents potentiate activity in a given target, whereas at other targets, the agent inhibits activity, which is denoted by: pot: potentiation and inhib: inhibition, respectively. The interaction of some agents with their targets was measured by binding affinity (bind); see detailed explanation in Supplemental Digital Content S2, https://links.lww.com/AA/F642.

We reviewed papers reporting in vitro concentration-response curves for the interactions of common anesthetic agents with target receptor activity; our method of data retrieval from the literature and analysis is presented in Supplemental Digital Content S2, https://links.lww.com/AA/F642. A summary of the key results from the perspective of the anesthetic agents (Figures 1 and 2) are presented (from 310 papers in total). We included only those studies presenting direct in vitro measurements of target-anesthetic interactions. For voltage-gated channels and ionotropic receptors, we used only data from electrophysiological measurements of current inhibition, activation, or potentiation. For metabotropic receptors (ie, G-protein coupled receptors; GPCRs), since end point measures of drug efficacy varied between studies, we present specific radioligand binding data as an indication of affinity. An exception to the latter was the muscarinic acetylcholine receptor (mAChR), as inhibition of a Ca^2+^-activated Cl^-^ current was frequently measured.

We focused on (a) the intravenous (IV) anesthetics propofol, etomidate, ketamine, and pentobarbital; (b) the contemporary inhaled anesthetics halothane, isoflurane, sevoflurane, nitrous oxide (N_2_O), and xenon (Xe); (c) the historical inhaled anesthetics diethyl ether, chloroform, cyclopropane, (d) IV agents generally used for sedation dexmedetomidine, diazepam, and midazolam. The agents in (c), albeit historical, nevertheless provide some insightful data concerning agent-target interaction. Agents used more commonly as adjuncts in (d) are nevertheless reported as sole agents for induction (eg, dexmedetomidine^9^ and midazolam^10^).

We investigated reports of their actions on: voltage-gated ion channels: Na_v_, K_v_, K_2P_, Ca_v_ and HCN (hyperpolarisation-activated, cyclic nucleotide-gated channel); ligand-gated ionotropic receptors: glycine receptors (GlyR), GABA_A_Rs, nicotinic acetylcholine receptor (nAChRs; both muscle and neuronal types), 5-hydroxytryptamine type 3 receptor (5-HT_3_), glutamatergic NMDA and AMPA (alpha-amino-3-hydroxy-5-methyl-4-isoxazolepropionic acid) receptors; and metabotropic muscarinic acetylcholine receptors (mAChRs), α2-adrenoreceptors (ARs), opioid receptors (ORs), and all other 5-HT receptors.

Several of these receptor subtypes were included for completeness, even though their role in the mechanisms of consciousness is far from established. The 5-HT_3_ receptor is generally viewed as being involved in nausea and vomiting. Although this is holistically relevant to anesthesia, these receptors have also been suggested to be involved in sleep mechanisms.^11^ These receptors also modulate synaptic release of many CNS neurotransmitters, including GABA, glutamate, and acetylcholine.^12^ The skeletal muscle nAChR is not present in the central nervous system (CNS). Yet, investigators have often used the receptor as a convenient model, and because immobility forms part of the clinical “triad” of anesthesia. Also, some emerging theories now more directly link muscle movement with consciousness.^13^ We present results from these receptor subtypes not directly involved in hypnosis separately from others (Supplemental Digital Content S2, https://links.lww.com/AA/F642.

Where targets were potentiated by anesthetics (mainly GABA_A_R and GlyR, and sometimes 5-HT_3_), studies typically reported potentiation in the presence of a background submaximal concentration of endogenous ligand (ie, GABA, glycine, or 5-HT). Literature data were often available for a range of control/background GABA/Gly/5-HT concentrations, but we restricted our data to potentiation of the most commonly used: ie, EC_10_-EC_20_ responses (concentrations eliciting 10%–20% of their maximal inducible response) for GABA, 5-HT and Gly (intravenous), and EC_5_-EC_20_ for Gly (volatiles); see Supplemental Digital Content S2, https://links.lww.com/AA/F642 for further detail. Reported values were converted into a percentage of I_max_ (ie, the maximal current inducible by a saturating concentration of GABA/Gly/5-HT) and the control response was subtracted to yield a concentration-response relationship representing the anesthetic-induced increase in target function.

For N_2_O and Xe, which are typically dosed by pressure (% atm), we extracted values for aqueous concentrations (mM) as reported by the authors. Where aqueous concentrations for N_2_O and Xe were not provided, saturated solutions of N_2_O and Xe were assumed to be 29.2 mM^14^ and 4.3 mM,^15^ respectively, under standard conditions. For cyclopropane, when the dose was reported in multiples of MAC, 1 MAC was assumed to be 0.84 mM,^16^ unless otherwise stated by the authors.

Where possible, data separation by receptor subtype has been preserved. This was dependent on the nature of the original data, for example, whether the authors specified “Ca_v_3.1” or “T-type Ca current.” We extracted data for multiple GABA_A_R subtypes and present α1β2γ2 as a representative, but the full spectrum of studied receptor subunit configurations is presented in Supplemental Digital Content S2, https://links.lww.com/AA/F642. Data were plotted as a series of concentration-response curves for each anesthetic agent and receptor. The graph for each anesthetic agent contains the subtypes of each receptor that are most sensitive to that agent as representatives and fit to standard ligand binding curves as described.

## KEY FINDINGS

### Intravenous Agents

Across all the receptors for which it has affinity, propofol (Figure 1A) shows the greatest potency at neuronal α4β2 nAChR (where it is inhibitory; IC_50_ 3.13 µM), and at clinical concentrations for the end point of immobility, both neuronal α4β2 nAChR and HCN1 channels (IC_50_ 4.19 µM) appear to be most affected (~20% inhibition). Propofol’s efficacy at HCN1 is high (E_max_ 96.6%), and is similarly so for GABA_A_R (E_max_ 91.9%) and α1β Gly receptor (E_max_ 92.4%). It is worth noting that the relevance of the in vitro inhibitory activity seen at HCN1 channels has been confirmed by in vivo gene deletion studies demonstrating that loss of HCN1 results in a 47% increase in the ED_50_ for hypnosis in mice.^17^

Etomidate, more so than propofol (Figure 1B) could be described as predominantly (albeit not exclusively) “GABAergic,” showing the highest potency at GABA_A_R (EC_50_ 1.08 µM). Interestingly, results on its action on Na_V_ channels fell into 3 distinct groups: one, which exhibited a higher potency (EC_50_ 0.393 µM) than for GABA_A_R, but the others had much lower potencies (EC_50_ 95.1 µM and 264 µM). We disregard the apparently more potent interaction of etomidate with rat NaV channel (EC_50_ 0.393 µM; Figure 1B) because a species difference clearly exists; the human channel exhibits much lower potency (EC_50_ 264 µM; and the mouse is also low at 95.1 µM). Across the concentrations studied, the highest efficacy occurs at neuronal α4β4 nAChR (E_max_ 100%).

Pentobarbital’s potency (Figure 1C and Supplemental Digital Content, Figure S2, https://links.lww.com/AA/F642) is a little higher at muscle nAChR (IC_50_ 25.1 µM) than for GABA_A_R (EC_50_ 27.9 µM). However, at clinically relevant concentrations in terms of efficacy, it is the neuronal α4β2 nAChR that is most affected (78% inhibition; Supplemental Digital Content, Figure S26A, https://links.lww.com/AA/F642). AMPAR (IC_50_ 76.4 µM), 5-HT_3_ (IC_50_ 111 µM), and NMDAR (IC_50_ 186 µM) receptors are also influenced, albeit to a lesser extent.

Ketamine (Figure 1D) shows the highest potency at the neuronal α4β4 nAChR (IC_50_ 0.144 µM), closely followed by NMDARs (IC_50_ 0.338 µM). At clinical concentrations, NMDAR is almost saturated for effect, with smaller effects on several other receptor types, but not GABA_A_R or Ca_v_. Efficacy is maximal at NMDARs (E_max_ 97.0%), mAChR (E_max_ 100%), and also T-type Ca_v_ channels (E_max_ 100%). Although from the receptor’s perspective, ketamine is the least potent of all the agents that bind to HCN1, and from the agent’s perspective, HCN1 is only in the middle of the potency range of targets ketamine binds to, there is evidence from HCN1 knockout mice that this receptor may play a key role in ketamine’s anesthetic action.^17^ This reflects that genomic studies are not always concordant with the pharmacological approach, as we discuss later.

### Inhalational Agents

For the volatiles, when separately viewed, halothane and isoflurane (see Figure 2 and Supplemental Digital Content S2, https://links.lww.com/AA/F642) show their highest potency at neuronal nAChRs (IC_50_ for α4β2 subtype 40.9 and 59.9 µM, respectively). Conversely, sevoflurane shows its highest potency at K_v_1.2 (EC_50_ 9.7 µM). At clinical concentrations for the immobility end point (minimum alveolar concentration, MAC), in efficacy terms, both halothane and isoflurane exert their greatest effect on neuronal α4β2 nAChR (81% and 73% inhibition, respectively), whereas for sevoflurane it is on TRESK (143% potentiation). In general, their efficacy (E_max_) is highest at K_2P_ channels (typically >100%), but appreciable effects occur at almost all receptor types studied, and in this regard, the promiscuity seen here is comparable to that observed with the more promiscuous intravenous agents.

N_2_O (Figure 2D) shows its highest potency at NMDARs (IC_50_ 12,100 µM), albeit with submaximal efficacy, whereas Xe shows its highest potency at AMPAR (IC_50_ 2481 µM). It is difficult to comment on their definitive efficacy at other receptor types as studies using a full range of concentrations were scarce. At clinical concentrations of these volatiles, NMDARs are most inhibited with N_2_O (71.4% inhibition), and 5-HT_3_ with Xe (63.4% inhibition). However, like the other inhaled agents studied, both N_2_O and Xe are promiscuous.

### Perspective From Molecular Targets

However, the view is different from the perspective of the molecular targets (see Supplemental Digital Content S2, https://links.lww.com/AA/F642). Generally, the literature takes the perspective of the diverse targets for agents; less common is the focus on the diverse affinity of a receptor for multiple agents. Just because an agent’s “preferred” (ie, in potency) is a certain target, promiscuity means that this target does not always reciprocate and exhibit the strongest affinity for this agent. Only sometimes is there a “match”; as with dexmedetomidine and the α2 receptor, ketamine and NMDAR, and, to a large extent, etomidate and GABA subtypes (Supplemental Digital Content, Figures S16A, S37, and S45, https://links.lww.com/AA/F642).

The α_1_β_2_γ_2_ GABA_A_R is most sensitive to etomidate (EC_50_ 1.08 µM), then propofol (5.44 µM), and to a much lesser extent, pentobarbital (27.9 µM). The concentration-response relationship for etomidate is rather flat with low efficacy (E_max_ 72.0%), whereas the highest efficacy occurs with propofol (E_max_ 91.9%). For the volatiles considered separately, EC_50_ values are several orders of magnitude higher than for the IV agents. This GABA_A_R subtype is also sensitive to isoflurane, sevoflurane, and halothane in an order of potency (at EC_50_ 405, 470, and 2164 µM, respectively).

K_2P_ channels are similarly sensitive to most volatiles and of the IV agents, apparently only to etomidate, which is, in contrast, inhibitory. Efficacy at each K_2P_ subtype varies by agent.

HCN channels are most sensitive to dexmedetomidine among the IV agents (see Supplemental Digital Content, Figure S24, https://links.lww.com/AA/F642; IC_50_ 0.14 µM), though the highest efficacy occurs with propofol (E_max_ 96.6%), and they are also sensitive to the common volatiles and Xe.

The α_2_ receptor is also exquisitely sensitive to dexmedetomidine (as measured by binding studies; K_D_ 0.0012 µM). If we compare the different agents’ potencies at the α2 receptor versus HCN, dexemedetomidine is several orders of magnitude more potent at the former than the latter, where its dose-response curve lies closer to that of other agents.

Similarly, the NMDAR shows considerable selectivity for ketamine, but also is quite sensitive to volatile agents; sevoflurane shows potency (IC_50_ 144 µM) that is comparable to pentobarbital (IC50 186 µM; Supplemental Digital Content, Figures S37, S38, https://links.lww.com/AA/F642).

Neuronal nAChRs are sensitive to ketamine (α4β4 subtype, IC_50_ 0.144 µM); their sensitivity to common volatiles (but not ether, cyclopropane, N_2_O and XE) is also high (for neuronal subtypes, EC_50_ ranges between 10.9 and 674 µM) Most of the agents studied here exhibit high efficacy at these receptors (E_max_ often, or close to, 100%).

## DISCUSSION

Our conclusions depend on perspective. If our starting point, for example, is that propofol is decidedly an anesthetic agent and we wish to know at which receptor it is most potent, the conclusion would be the neuronal α4β2 nAChR or HCN1 (IC_50_ 3.13 and IC_50_ 4.19 µM, respectively) and not at α1β2γ2 GABA_A_R, the most common GABA_A_R subunit combination in the human brain (EC_50_ 5.44 µM).^18^ At other receptors, it is less potent; so by the terminology in common current use, we should logically describe propofol as typically or predominantly “anticholinergic,” or “HCNergic,” not “GABAergic.” Similarly, halothane and isoflurane would also be described as “anticholinergic,” as for their actions they are most potent at neuronal α4β2 nAChRs (the most common nAChR subtype in the CNS). We could therefore go on to say that general anesthesia, conducted commonly using a combination of propofol induction and isoflurane maintenance (or propofol total intravenous methods) “predominantly” arises as a “central anticholinergic phenomenon.”

If, on the other hand, our starting point is to consider potentiation of GABA_A_Rs with the above configuration to be pivotal to anesthetic-induced hypnosis (unconsciousness), and to ask which agent has the greatest affinity for this particular target (ie, which is the most potent), then we would conclude it is of the IV agents, etomidate (EC_50_, 1.08 µM). The modern volatiles have much lower potency than the IV agents, but similar potency here to each other. We would then say that “typical” GABAergic agents are, for IV agents, etomidate (but not propofol, EC_50_ 5.44 µM; or pentobarbital, EC_50_ 27.9 µM), and separately considered, the volatiles isoflurane (EC_50_ 405 µM) and sevoflurane (EC_50_ 470 µM) but not halothane (EC_50_ 2164 µM).

The terminologies used in the above paragraph are clearly simplistic, but no less simplistic than the current paradigm of classing agents predominantly as “GABAergic,” “NMDAergic,” etc. A different, more sophisticated, approach would be to accept that: (a) all the agents examined here are general anesthetics; (b) they demonstrate affinity for molecular targets in a promiscuous manner; and (c) the putative molecular targets contributing to hypnosis are equally promiscuous for the agents they bind to. Arguably, many of the authors illustrated in Supplemental Digital Content S1, https://links.lww.com/AA/F642 do recognize this, but nevertheless, the language in their articles, and many more like them, emphasize a dominant if not exclusive interaction with a single target. For example, Brown et al.^5^ describe the sequence of events occurring after administering a “GABA agonist,” and explicitly discuss the “mechanisms of GABA-mediated altered arousal,” all as being distinct from those elicited by “NMDA antagonists.”

Before we combine these 3 notions (a-c) above, it is necessary to consider features not encompassed within our concentration-response relationships, which complement and lend greater completeness to our analysis.

### Relevant Factors Other Than Potency/Efficacy in Concentration-Response Relationships

One is the issue of “clinically relevant concentration.” It is often assumed that all results obtained in vitro at concentrations much higher than those applied clinically should be disregarded as irrelevant or potentially toxic effects.^3^ In this regard, we have marked on our plots the approximate concentration for the clinical end point of immobility (which is greater than that required for hypnosis). Eckenhoff and Johansson, however, have pointed out the flaws in that argument.^19^ In vivo, there exist numerous elements such as the cytoskeleton, soluble proteins, and other large molecules that are space-occupying and reduce the volume available for water and ligands such that the thermodynamic activities, and hence binding equilibria, of the latter can be significantly affected. It has also been suggested that brain concentrations can be higher than those seen in plasma.^20^ As Eckenhoff and Johansson argue, the tightly controlled experimental conditions of in vitro studies may allow ligands to behave in a more thermodynamically ideal manner. Further, there is no necessary direct correlation between a concentration-response relationship in vitro and the population dose-response relationship obtained from clinical or whole-animal studies. The former is constructed from direct measurements of continuous effects across very wide dose ranges to identify the concentrations needed for saturation of effect (and hence the EC_50_, Hill slope, and other parameters). The latter is a more subjective construct derived from a binary effect (awake versus unconscious) in each animal/subject, the “effect” being the proportion of these reaching the given end point. The population might be considered “saturated,” but it is unknown if this is also the case for the protein targets of each individual within it. What happens at the molecular target level if we continue to administer an anesthetic to an animal that has reached the point of unresponsiveness may provide insights into relevant actions at the cell or tissue level that cannot be readily measured clinically due to the unresponsiveness of the animal. Therefore, in the concentration-response curves presented here, it would be misleading to consider only those results in the narrow “therapeutic” dose range for each agent that have been indicated, and discard the rest, as we seek more properly to classify agents. Efficacy could be as relevant as potency in characterizing the “predominant” molecular target/s for an agent.

In turn, this is related to a second factor, which is the (unknown) magnitude of effect at the target that contributes to anesthesia. If an agent such as propofol has a strong effect at a receptor such as the neuronal α4β2 nAChR at a clinically relevant concentration (eg, ~20% inhibition at 1.1 μM propofol) but affects other receptors less at this concentration (eg, ~8% inhibition at Na_v_), and if nAChR is a putative mediator of hypnosis, then we might be tempted to conclude that the nAChR interaction is the main (or only) one relevant to anesthesia. What this interpretation ignores is that we do not know how these respective effects at the receptor level (20% at nAChR vs 8% at Na_v_) translate to the desired behavioral response. It is entirely possible that the smaller effect on Na_v_ is more important physiologically than the larger nAChR effect.

A third factor is the distribution and quantity of targets.^21^ The exquisite sensitivity of an anesthetic at a single receptor may be striking but meaningless if hardly any are expressed anywhere, especially in brain regions relevant to the behavioral end point of interest. Equally, if an anesthetic has only a very weak effect at a different protein target, but one that is widely expressed and distributed across those relevant brain regions, this second interaction evidently becomes the more pertinent interaction.

A fourth factor is that in vivo, receptors can behave differently in their natural environment than in vitro. We illustrate using our experience with the background 2-pore acid-sensitive potassium channel (TASK) in carotid body oxygen sensing, which has several subtypes, of which TASK-1 and TASK-3 are the most relevant and can be studied separately as expressed channels.^22^ However in vivo, they dimerize and the heterodimer behaves uniquely.^23^ Another example is the endogenous mechanisms by which TASK channel activity can be regulated by “SUMOylation” through the binding of a small ubiquitin-like modifier (SUMO) polypeptide.^24^

In relation to this, account should also be taken of there being multiple binding sites on some receptors, like TASK and notably, GABA. This last is typified by benzodiazepines, which bind selectively to a high-affinity site on GABA_A_ receptors where they are low-efficacy agonists. At much higher concentrations they act on other sites on GABA_A_ receptors, including one common to etomidate, again with low efficacy.^25^ The combined effects of benzodiazepine occupying both sites are likely sufficient to induce general anesthesia.^10^ There are also “peripheral benzodiazepine receptors” in mitochondria that enhance sedative neurosteroid synthesis; an effect which is uncertainly linked to anesthesia.^25^ These dose-related interactions could in fact be viewed as consistent with multi-site notion of “promiscuity.”

A fifth factor is that we have not taken full account of in vivo genetic manipulation studies. The principle here is that by modifying the expression or structure of a single putative molecular target, more precise information can be obtained concerning individual drug-receptor binding interactions, and the extent to which those interactions may contribute to the anesthetic-induced state. Sometimes there is concordance between genomic studies and in vitro electrophysiological data: to wit, the concentration-response curve is right-shifted (*ie*, MAC for loss-of-righting reflex is increased) in mice wherein the GABA_A_R α1 subunit was engineered to be insensitive to isoflurane and enflurane (but not halothane) in vitro.^26^ At other times, however, there is discordance between these approaches. For example, deletion of the GABA_A_R α6 subunit has no effect on various anesthetic (both IV and volatile) end points,^27,28^ yet these agents positively modulate α6 subunit-containing GABA_A_Rs in vitro. Conversely, deletion of the NMDARε_1_ subunit reduces sensitivity to propofol in mice, but our summary results would suggest there is little or no effect on NMDARs in vivo at clinical concentrations in the first place.^29^ Independently from our in vitro concentration-response analysis, these results imply that action at multiple targets is necessary, since no single genetic manipulation completely abolishes sensitivity to anesthesia, but only alters it.

## CONCLUSIONS: SINGLE- VS MULTISITE THEORIES

There are broadly 2 frameworks for understanding molecular mechanisms of anesthesia.^30–32^ One is that there is a key, unique “Holy Grail” molecular target, affected by all agents, which is consistent with a unitary mechanism or single-site hypothesis. This notion might include the “lipid theory” in which the membrane is the common target, or the GABA_A_R within the “protein theory” of anesthetic action. The alternative, multi-site hypothesis is that each agent acts on several targets, with each anesthetic-receptor combination contributing in some fashion to the final effect.

Although the terminology often used with respect to agent action (“GABAergic,” etc) is more consistent with the former hypothesis, the indisputable promiscuity of both agents and their targets demonstrated here strongly suggests that the latter, multi-site hypothesis, is the more likely. When any drug like propofol is administered, it will deterministically bind to all the receptors illustrated here. Although we cannot be certain that each interaction will contribute to the resulting anesthetic state, the opposite (ie, that of all the interactions it is only one, for example, GABA_A_R, that is clinically relevant) is highly improbable.^33^ This interpretation has been offered before^34–36^ and is separately supported by the genetic manipulation literature discussed above.^32,37^

It is notable within our data that those agents which are amongst the least promiscuous (eg, diazepam and midazolam for α1β2γ2 GABA_A_R; dexmedetomidine for α2 AR) are also those not routinely, if ever, used solely to provide maintenance anesthesia for sustained surgeries. The same might be said of ketamine (NMDAR): the highly selective NMDAR inhibitor MK801 does not produce anesthesia (in rodents, as measured by loss of righting reflex).^38^ For all these agents, reports of their use as sole anesthetics (eg, ketamine for minor procedures, generally in lower- and middle-income countries^39^), may be due to high dosing that then affects receptors other than their most potent target. We could therefore say that “monogamy does not produce anesthesia.”

An analogy for the multi-site hypothesis is to consider which element of a computer, car engine or central heating system should be regarded as the “most important.” All elements play their part, and contribute to overall function. Disrupting multiple components in a small way can seriously impair the machine’s operations. Even though most focus to date has been on the GABA_A_R, it is evident even from first principles that these receptors can only have a limited contribution. Patients with antiGABA_A_R antibodies remain sensitive to anesthetics,^40^ and those with antiNMDAR do not appear to exhibit any altered clinical sensitivity to ketamine.^41^ Equally in this analogy, just as a car can cease to function due to factors other than its engine structure (eg, lack of fuel), function can be disrupted in ways other than through presumed anesthetic action at cell surface targets. Anesthetic- and genetically-induced mitochondrial dysfunction reduce ATP production, which inhibits synaptic transmission, independent of GABAergic effects.^42^

Studies at meso-level face a dual challenge in their interpretation. On the one hand, there may be a commonality of signatures because there is a likely to be some common feature (eg, a final common pathway) to being unconscious. On the other hand, there may be unique features related to the agent’s distinct molecular spectrum. To continue the analogy, disrupted function may be seen as a slow speed or noise in a car, but many different underlying problems can lead to this common outcome. Thus Akeju et al. reported that sevoflurane, like propofol, induced coherent frontal α and slow oscillations, even though their molecular spectra of activity are dissimilar.^43^ They noted, however, that sevoflurane also exhibited a distinct θ coherence signature, which could in retrospect reflect the differences in molecular activities. In other words, classifying EEG, fMRI and other functional imaging patterns as “agent-specific” (an approach used by Purdon et al.^4^) is likely to be more precise and better reflect a multi-site hypothesis than attempting to classify them as “target-specific.”

### Avenues for Research

The development of high-affinity selective modulators for different molecular targets, like MK801 mentioned above, could be useful for understanding mechanisms of anesthesia. Even if these agents are not in themselves anesthetics, each could be used to characterize the less-than-anesthesia picture (including EEG signature) of a given molecular target being specifically modulated. This could build, bottom-up as it were, a full picture of “anesthesia” were several such highly specific agents to be used together. Using mixtures of these in different combinations could be used, as proof of principle, to mimic the spectrum of phenotypic and EEG responses as obtained with common agents like propofol or barbiturates. The prediction would be that phenotypic and EEG responses would be similar for the mix of high-affinity single-receptor agents, as for the commonly used promiscuous anesthetics.

Our analysis suggests one other avenue using mixtures that might help further discriminate between the single- vs multi-site hypotheses, which is to study agent combinations depending on their potency/efficacy at the given target. In this argument, we use the term “agonist” to refer to the ability of a ligand to alter the *function* of a given receptor, regardless of whether the change in functionality is activation/potentiation or inactivation/inhibition. Thus, although the effect of ketamine is to reduce the function of HCN1 channels (as measured by a decrease in maximal current and a left-shift in the half-activation potential, V_1/2_),^17^ we consider it an “agonist” using our agnostic functional definition above. Based on our definition, ketamine behaves as a “partial agonist,” as it fails to reach the maximum effect of other agents, like propofol, which might be considered a full agonist at this receptor. Similarly, halothane, isoflurane and sevoflurane appear to be “partial agonists” at GABA_A_Rs, where again propofol might be considered a full agonist.

Classical pharmacology predicts that mixtures of a partial and full agonist should result in mutually competitive, infra-additive behavior at the receptor or channel. This is notwithstanding the possibility of different actions at the binding site (ie, propofol is an allosteric modulator of GABA_A_Rs and so may not directly “compete” with other drugs that may be orthosteric modulators), or different binding sites within the target (see below). For these situations, partial agonist behavior on channel function by one agent would not then antagonize full agonist behavior by another agent. Nevertheless, mutually competitive inhibition has already been confirmed for halothane (full agonist) and isoflurane (partial agonist) at the TASK channel.^44^

**Figure 3.**
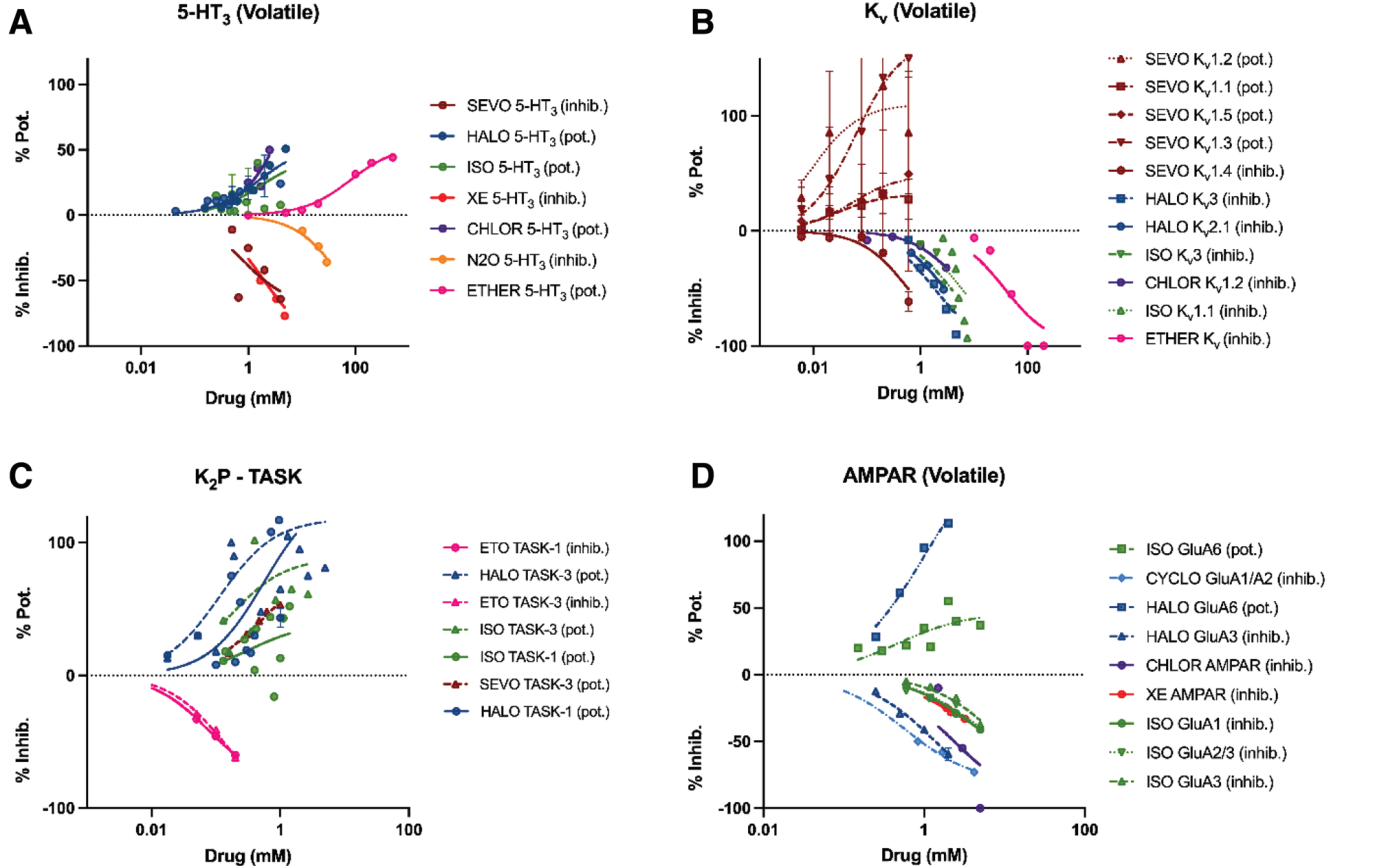
Concentration-response relationships for anesthetics with 4 receptors where some agents potentiate activity, whereas other agents inhibit activity. In a single-site hypothesis, these receptors cannot be unique targets that underlie anesthetic action (see text for argument). The *y*-axis here, in contrast to Figures 1 and 2, is divided between the % potentiation (Pot) or inhibition (Inhib) and each agent specified as to whether it potentiates (pot) or inhibits (inhib). See detailed explanation in Supplemental Digital Content S2, https://links.lww.com/AA/F642.

Moreover, we identified 4 target proteins (5-HT, K_2P_, K_v_, and AMPAR; Figure 3) where the action of agents can be bidirectional. Some anesthetics activate these, whereas others inhibit them. By definition, any single-site theory of anesthesia cannot rest on these targets as it is not possible to explain the same biological result both by activation and inhibition of the same process. This does not preclude these receptors from making some contribution to the conscious level, and each agent influences the resulting neurocognitive state in subtle ways, on the one hand through receptor activation, and on the other hand through inhibition.

If the single-site hypothesis were true, an infra-additive interaction of 2 agents at a target that was key to mediating consciousness should predictably translate to the whole-body level, where such mixtures would produce less hypnosis (ie, higher MAC) than with the full agonist alone. If, on the other hand, mutually antagonistic behavior is observed at a single target, but never at whole-body level, this would undermine the notion of a “key” single-site of action and be more consistent with a multi-site hypothesis. For instance, the TASK channel is known to be involved in halothane and isoflurane anesthesia, as knockout of TASK-1 and/or TASK-3 produces a right-shift in the concentration-response curve for immobility (ie, increased MAC).^45^ Yet, in contrast to their interaction at TASK, where they exhibit competition,^44^ their MACs are additive.^46,47^

In principle, a systematic study of mixing the full agonists at each receptor with the corresponding partial agonist would identify the combinations (and hence the key receptor/s), if any, that resulted in “less anesthesia.” One such “key target” may be HCN1,^48^ where our results show clear differences in efficacy of propofol and ketamine (Supplemental Digital Content, Figure S24, https://links.lww.com/AA/F642). Propofol and ketamine have been found to interact infra-additively in humans,^46^ and a theoretical modeling approach^49^ suggests that the EEG spectral changes induced by ketamine-propofol mixtures arose from their infra-additive interaction at HCN1 (as opposed to GABA_A_R or NMDAR). Infra-additive effects of mixtures may more readily be seen in single-channel or cell studies, but demonstrating any effects on sedation or anesthesia in vivo may be limited by (a) low receptor occupancy producing the desired effects and (b) where drugs act on multiple targets (eg, the benzodiazepine example above), synergy may occur at 1 target along with competition at another, again, obscuring interactions. In this regard, suitable end points will need to chosen carefully. For example, ketamine’s sympathomimetic activity raises processed EEG scores,^50^ likely by increasing monoaminergic signaling in the cerebral cortex. Combining other anesthetics with ketamine may appear to be subadditive because ketamine is a circuit-level neuro-activator, not because it competes with other drugs for receptor occupation.

In summary, our analysis of in vitro pharmacologic data reveals considerable promiscuity in both anesthetics and receptor behavior. This undermines the notion that we should regard agents as primarily GABAergic, others as NMDAergic, etc. With few exceptions, this terminology is unhelpful and misleading, especially as many of those agents conventionally classed in these ways are in fact more potent or efficacious at completely different target sites. Promiscuity strongly favors the multi-site, rather than the single-site, hypothesis for anesthetic action. Our overview leads to a specific approach that might help resolve which hypothesis is the more likely correct, involving study of agent mixtures to assess additivity or infra-additivity. We suggest that the commonly used single-target nomenclature should be abandoned in favor of one that more accurately reflects the spectrum of target activity for the anesthetic in question.

## DISCLOSURES

**Conflicts of Interest:** J. J. Pandit is Editor-in-Chief, *Anesthesia & Analgesia*, and was not involved in the handling of this article. No funding or competing interests declared. P. A. Goldstein is Section Editor, *Anesthesia & Analgesia*, and was not involved in the handling of this manuscript. He is a coinventor on patents related to the development of novel alkylphenols for the treatment of neuropathic pain and serves on the Scientific Advisory Board for Akelos (New York, NY), a research-based biotechnology company that has secured a licensing agreement for the use of those patents. No other authors declared Conflicts of Interest. **Funding:** K. M. Kale was funded by a grant from the *British Journal of Anaesthesia* and Royal College of Anaesthetists. **This manuscript was handled by:** Ken B. Johnson, MD.

## Supplementary Material
