# Supplementary material for: Are “GABAergic” Agents Really So Selective for GABA? Implications for Single- versus Multi-Site Hypotheses From Promiscuous Behavior of Anesthetics and Their Molecular Targets In Vitro
Source: Anesth Analg. 2026 Jan 23;142(6):1039–49. doi: 10.1213/ANE.0000000000007888 (PMC13155216; doi:10.1213/ANE.0000000000007888)
Supplement: Supplementary file 1 [file ane-142-1039-s001.pdf]

SUPPLEMENTARY DIGITAL CONTENT

S1 Examples of terminology in the literature classing anesthetic agents by a presumed predominant action on a single molecular target

Twelve articles are selected as illustrative of (a) use of the terms GABA-ergic (or sometimes also 'NMDA-ergic) and so on [we highlight the relevant language in bold]; (b) implying a high degree, if not exclusive selectivity of action of agents at a specific receptor. The Brown et al. paper specifically seeks to classify agents according to this predominant molecular target action.

[1] Brown EN, Purdon PL, Van Dort CJ. General anesthesia and altered states of arousal: a systems neuroscience analysis. Annu Rev Neurosci. 2011; 34: 601-28.

"We perform a systems neuroscience analysis of the altered arousal states induced by five classes of intravenous anesthetics by relating their behavioral and physiological features to the molecular targets and neural circuits at which these drugs are purported to act...that is, relating the actions of the drugs at specific molecular targets in specific neural circuits to the behavioral and physiological states that comprise general anesthesia."

"We review the altered arousal states of **five classes** of intravenous anesthetic drugs: gamma-amino butyric acid type A **(GABAA) receptor agonists**, opioid receptor agonists, N-methyl D-aspartate receptor **(NMDA) antagonists**, **alpha 2 receptor agonists**, and dopamine receptor antagonists."

[2] Turski CA, Ikonomidou C. Neuropathological sequelae of developmental exposure to antiepileptic and anesthetic drugs. Front Neurol. 2012; 3: 120.

"Sedative and **anesthetic drugs interact with glutamate and GABA receptors to produce their desired effects**..."

[3] Khan KS, Hayes I, Buggy DJ. Pharmacology of anaesthetic agents I: intravenous anaesthetic agents. Cont Educ Anaesth, Crit Care Pain. 2014; 14: 100-105.

"...**most agents exert their action through potentiation of GABAA receptor activity**....the Unitary Hypothesis stated that while known general anaesthetics are chemically diverse agents, they produce their anaesthetic effects by a similar (unknown) mechanism...."

"Potentiation at GABA A receptors by volatile anaesthetics and inhibition at N-methyl- D -aspartate (NMDA) receptors by the anaesthetic gases N2O and xenon are likely to be important mechanisms of action."

[4] Brohan J, Goudra BG. The Role of GABA receptor agonists in anesthesia and sedation. CNS Drugs. 2017; 31: 845-856.

"**The GABAA receptor (GABAAR) has a central role in modern anesthesia** and sedation practice, which is **evident from the high proportion of agents that target the GABAAR**. Many GABAAR agonists are used in anesthesia practice and sedation, including propofol, etomidate, methohexital, thiopental, isoflurane, sevoflurane, and desflurane."

“**The central role of the GABAAR in modern anesthesia and sedation practice is evident from the high proportion of agents that target the GABAAR**. Of the ten inhalational and intravenous anesthetics traditionally used to induce or maintain general anesthesia (nitrous oxide, isoflurane, sevoflurane, desflurane, xenon, propofol, etomidate, ketamine, methohexital, and thiopental), seven agents enhance the function of GABAARs. **Ketamine, nitrous oxide, and xenon, in contrast, inhibit inotropic glutamate receptors—mainly NMDA** receptors. In addition to general anesthetic agents, **benzodiazepines exert their hypnotic and amnesic effects by activation of the GABAAR**.”

“The specific effects of GABA-aminergic anesthetic and sedative agents have been linked to different GABAAR subtypes present in different brain regions.”

[5] Sahinovic MM, Struys MMRF, Absalom AR. Clinical pharmacokinetics and pharmacodynamics of propofol. Clin Pharmacokinet. 2018; 57: 1539-1558.

“Propofol is an intravenous hypnotic drug that is used for induction and maintenance of sedation and general anaesthesia. **It exerts its effects through potentiation of the inhibitory neurotransmitter gamma-aminobutyric acid (GABA) at the GABAA receptor**...”

[6] Brown, E. N., Pavone, K. J., & Naranjo, M. (2018). Multimodal general anesthesia: theory and practice. Anesthesia and Analgesia, 127(5), 1246–1258.

“The intravenous anesthetic propofol and the inhaled ether anesthetic sevoflurane...**primary targets of these anesthetics are the γ-aminobutyric acid subtype A (GABAA) receptor** synapses”

[7] Saxena N, Muthukumaraswamy SD, Richmond L, Babic A, Singh KD, Hall JE, Wise RG, Shaw AD. A comparison of GABA-ergic (propofol) and non-GABA-ergic (dexmedetomidine) sedation on visual and motor cortical oscillations, using magnetoencephalography. Neuroimage. 2021; 245: 118659.

“**Propofol was used as the representative GABA-ergic drug** in this study...”

“Future study designs with **alternative GABA-ergic sedatives (such as midazolam, which acts only on GABA-A receptors**)”

[8] Tanaka R, Tanaka S, Hayashi K, Iida K, Sawa T, Kawamata M. The histamine H1 receptor antagonist hydroxyzine enhances sevoflurane and propofol anesthesia: A quantitative EEG study. Clin Neurophysiol. 2022; 132: 2054-2061.

“**GABAergic anesthetics** hyperpolarize the thalamic and cortical circuits and change the thalamus to the oscillatory mode...Thus, bicoherence analysis is suitable for assessing the anesthetic depth induced by **GABAergic anesthetics**.”

“...**propofol and sevoflurane**, which is a widely used volatile anesthetic, **have similar GABAergic neural circuit mechanisms** for inducing unconsciousness...”

“...**α and δ oscillations** of an EEG, which **are considered to be markers of loss of consciousness induced by GABAergic anesthetics** in a clinical setting.”

[9] Luppi AI, Mediano PAM, Rosas FE, Allanson J, Pickard JD, Williams GB, Craig MM, Finoia P, Peattie ARD, Coppola P, Owen AM, Naci L, Menon DK, Bor D, Stamatakis EA. Whole-brain modelling identifies distinct but convergent paths to unconsciousness in anaesthesia and disorders of consciousness. Commun Biol. 2022; 5: 384.

"...a key role of spatially-specific local inhibition for reproducing the functional MRI activity observed during anaesthesia with **the GABA-ergic agent propofol**..."

"**Since propofol is a well-known GABA-ergic agonist**, these results confirm...Likewise, our framework could be adapted to model individual susceptibility to **anaesthesia with GABA-ergic agents**..."

[10] Niu Y, Yan J, Jiang H. Anesthesia and developing brain: What have we learned from recent studies. Front Mol Neurosci. 2022; 15: 1017578.

"**General anesthetics act primarily by blocking N-Methyl-D-Aspartate (NMDA) recepters and/or activating gamma-aminobutyric acid (GABA) receptors**...."

[11] Durga P, Singham G, Baradaa A. Understanding the GABA-A receptor: implications for anesthesia and beyond. J Neuroanaesthesiol Crit Care 2023;10:155–166.

"The widely used **positive GABA A R modulators include benzodiazepines (anxiolytic and anticonvulsant), general anesthetics** (volatile agents like isoflurane, and intravenous agents like barbiturates, etomidate, and propofol)..."

[12] Mogianos K, Persson AK. Anesthesia depth monitoring during opioid free anesthesia - a prospective observational study. BMC Anesthesiol. 2025; 25: 37.

"...there are currently no published data on what to expect, regarding neurophysiologic signaling and pEEG-pattern, when combining **GABA-ergic, NMDA-antagonists and alpha2-agonists** as well as non-sedating agents such as NSAID, betamethasone and paracetamol.

S2: Details of methods and results of literature review

Data Collection

We searched PubMed and Web of Science for various combinations of sedatives/anaesthetics and receptors, e.g. 'propofol' and 'sodium channel', 'Na channel', 'Na_v' etc. Abstracts were screened to identify potentially relevant papers, which were then examined for *in vitro* dose-response data corresponding to drug effects on a defined channel/receptor. The reference lists of retrieved papers were also reviewed.

We sought to examine the IV anaesthetics propofol, etomidate, ketamine and pentobarbital; the volatile anaesthetics diethyl ether, chloroform, cyclopropane, halothane, isoflurane, sevoflurane, nitrous oxide (N_2O) and xenon (Xe); and the IV sedatives dexmedetomidine, diazepam and midazolam. The effects of these agents on ionotropic Na_v, K_v, K_{2P}, Ca_v, HCN, GlyR, $GABA_AR$, nAChR, 5-HT_3, NMDAR and AMPAR; and metabotropic mAChR, α_2-AR, opioid receptors, and all other 5-HT receptors were investigated.

Inclusion and exclusion criteria

Only papers presenting direct *in vitro* measurements of a receptor being affected by an anesthetic were included. For ion channels and ionotropic receptors, only data from electrophysiological measurements of current inhibition or activation/potentiation were used. Where current inhibition was available for multiple holding potentials (V_H), we obtained values at, or close to, -70mV (physiologic membrane potential).

We excluded all non-mammalian data because we noted major discrepancies in target sensitivity to an anaesthetic between older studies that used non-mammalian species (e.g. frog or squid axons).

Our initial search (employing key words such as propofol, etomidate, etc; $GABA_AR$, NMDAR, etc.; and combinations thereof) yielded 14437 articles. We excluded 430 abstracts, letters, opinion pieces like editorials, retracted publications, erratums and reprints, and included only 14007 full papers. Screening of abstracts (see inclusion/exclusion criteria) left 2245 papers for inspection of the full paper. In total, data from 310 papers are presented.

Data analysis

For metabotropic G-protein coupled receptors (GPCRs), since endpoint measures of drug efficacy varied between studies, we also present specific radioligand binding data as an indication of affinity. An exception to the latter was mAChR, as inhibition of a Ca^{2+}-activated Cl^- current was frequently measured.

Exact values for drug effect and concentration were extracted when listed by the original authors. In most cases, however, these were not available, and we therefore estimated values by careful analysis of the original graphs. Where several results were obtained at the same drug concentration, only the mean value for effect, and we plotted the data with its SEM for effect as error bars.

We then plotted the extracted data points on 'dose-response' plots using the equation below, and fit the compiled data for each dose-response relationship using non-linear regression with a standard Hill slope (1.00) (GraphPad Prism Vers. 10.5.0 (GraphPad Software Inc, La Jolla, CA). A minimum of three data points, plus zero, were required for curve fitting. The goodness of fit parameters (degrees of freedom and sum of squares) for each curve are listed in the accompanying data tables.

$$V = \frac{V_{max}[S]}{[K_{0.5}] + [S]}$$

Where V = effect (inhibition or potentiation); V_{max} = maximal effect; [S] = drug concentration; $[K_{0.5}]$ = IC_{50}/EC_{50}

If fewer than 3 data points were available, or there were >3 data points but these were unfittable (indicated by ^), the drug-receptor interaction was excluded from plots and instead the available values are listed in the accompanying tables.

Where target function was potentiated by anaesthetics (mainly $GABA_AR$ and GlyR, and sometimes 5-HT_3), most studies reported potentiation in the presence of a submaximal concentration of endogenous agonist (i.e., GABA, glycine, or 5-HT). Data were available for a range of such control or background GABA/Gly/5-HT concentrations but were most often in the EC_{10}-EC_{20} range (i.e., GABA/Gly/5-HT concentrations eliciting 10-20% of their maximal own inducible response). We therefore restricted our data to potentiation of EC_{10}-EC_{20} currents for GABA, 5-HT and Gly (the last for intravenous agents). This meant exclusion of 22 of 99 papers for $GABA_AR$; none for 5-HT. However, for studies of volatile anesthetic action at GlyR, an experimental norm appeared to be to use control/background concentrations in the EC_5-EC_{20} range, and so excluded 5 of 23 papers for GlyR (volatiles). The exclusions were across all the agents, such that the reported model fits (EC50 or IC50 values) for any single agent were not affected by including these; although the model fits were worse due to the greater heterogeneity of data (e.g., for propofol-GABA interaction the EC50 5.47μM with inclusions and 5.44 μM as reported below, after exclusions).

Reported literature values were converted to, and are expressed here as, a percentage of I_{max}, where 100% I_{max} equates to the maximal current induced by a saturating concentration of the endogenous ligand. The averaged control (basal 10-20% I_{max}) response was subtracted

from each data point to yield a concentration-response relationship representing the anesthetic-induced increase in target response.

Where possible, data separation by receptor subtype has been preserved. This was dependent on the nature of the original data, e.g. whether the authors specified '$Ca_v3.1$' or 'T-type Ca channel'. One exception to this was where the basic structure and function of a receptor was highly conserved across species (i.e., the receptor homologues), as their sensitivity to the relevant anesthetic agents was similar between species. In these cases, results were grouped under the human nomenclature for the receptor (e.g., the GluN1 NMDAR subunit in humans is referred to as ε1 in mice).

Data presentation

The graph for each drug usually contains the receptors subtypes that are most sensitive to the agent as representatives. An exception to this was $GABA_AR$ owing to the large number of possible subtypes; instead, data for the $\alpha_1\beta_2\gamma_2$ subtype (by far the most common) were selected[1].

Receptor types are consistently colour-coded across graphs for clarity. Separate receptor graphs for IV and volatile agents are also presented, which include all available data for drug-receptor subtype interactions.

The EC_{50}, IC_{50} or K_D for each drug-receptor combination is listed along each graph. *n for* the number of data points used, and for the number of papers are included.

Etomidate, Ketamine and Dexmedetomidine

These agents were studied in both enantiomer and racemic preparations. Here, we included data only for racemic etomidate and ketamine, and for dexmedetomidine (but not levomedetomidine or medetomidine).

N_2O, Xe and Cyclopropane

As N_2O, Xe are typically dosed by pressure (% atm), we converted all values to aqueous concentrations (mM) as per the original authors' measurements. Where soluble concentrations for N_2O and Xe were not reported, 100% N_2O and 100% Xe were taken to be 29.2 mM[2] and 4.3 mM[3], respectively, under standard conditions. Cyclopropane was often reported in multiples of MAC, and 1 MAC was assumed to be 0.84mM[4], unless otherwise stated by the authors.

Determination of 'clinical' concentrations

For the IV agents, published values for plasma concentration at which 50% of subjects are immobilised (Cp50 for immobility) were obtained for propofol, etomidate, ketamine and pentobarbital, as below (Table S1).

Table S1. Cp50 immobility for propofol, etomidate, ketamine and pentobarbital,

**Anesthetic**	**Cp50 Immobility (μM)**
Propofol	1.1*[5–8]
Etomidate	1.5[9]
Ketamine (racemic)	12.4**[10]
Pentobarbital	50.0^[11]

** For propofol, older studies reported values of 15.2[5] and 14.3[6] mg/ml, whereas more recent studies are 4.47[7] and 5.2[8] mg/ml. An average of 10 mg/ml was therefore taken and assuming 98% protein binding, this corresponds to 1.1 μM*
***For surrogate measure loss of eyelash reflex. Plasma protein binding reported as 10-30%[12], thus assumed to be 20% in calculations.*
^A directly measured concentration during human anaesthesia was not available and was therefore estimated as follows. The therapeutic concentration for pentobarbital is estimated at ~30 mg/L[13,14] and assuming 61% plasma protein binding[15], this corresponds to 51.8 μM, which is similar to Franks & Lieb's earlier estimation of 50 μM[11].

For the volatile agents, human aqueous MAC equivalents for were obtained from previously reported values[4], as below (Table S2).

Table S2. Human aqueous MAC equivalents from previously reported values

**Anesthetic**	**MAC (μM)**
Halothane	220[4]
Isoflurane	280[4]
Sevoflurane	340[4]
Diethyl Ether	9,500[16]
Cyclopropane	840[4]
Chloroform	790[16]
Xenon	3,100*
Nitrous Oxide	30,400*

**For xenon (MAC 72%[17]) and N_2O (MAC 104%[17]), aqueous MAC equivalents were calculated assuming saturated solutions of N_2O and Xe to be 29.2 mM[2] and 4.3 mM[3], respectively.*

For dexmedetomidine, diazepam and midazolam, measured values for sedative concentrations were obtained and adjusted for plasma protein binding, as below (Table S3).

Table S3. Sedative concentrations for dexmedetomidine, diazepam and midazolam

**Agent**	**Sedative conc. (nM)**
Dexmedetomidine	0.569*[18]
Diazepam	34.0[19]
Midazolam	76.7**[20]

* *Assuming 94% plasma protein binding*[21].
** *Taking value 500ng/ml for deep sedation and assuming 95% plasma protein binding*[22].

General comments on figures and tables

The following plots are the complete results, with the three plots from the main paper repeated for completeness.

The y-axis scale is shown for the magnitude of 'effect of agent', regardless of whether the agent's action is activating or inhibitory on the target. The scale is as a % potentiation or inhibition, where the former is marked as 'pot.' and the latter as 'inhib.'. Inhibition cannot be >100%. However, potentiation can be >100% for studies where a background endogenous ligand is employed (e.g., GABA) and the agent's modulatory effect is greater than this baseline activity.

A vertical dotted line is shown approximating the Cp50 for immobility (IV agents), or minimum alveolar concentration (MAC, for inhalational agents)

Within each panel of the figure, the color coded legend is ordered by the EC_{50} value.

Below each figure is the detailed table of results, ordered first by agent, then within an agent, by the EC_{50} value.

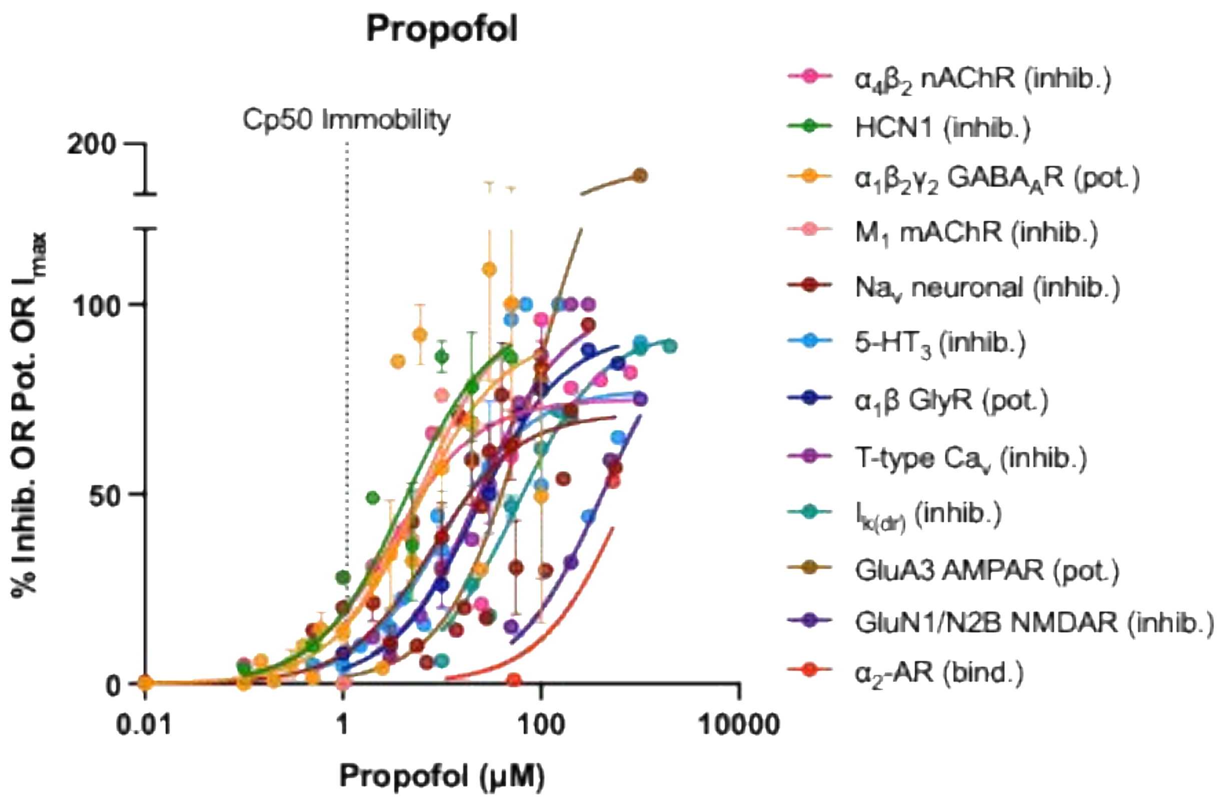

Figure S1

Receptor (subtype)	$EC_{50}/IC_{50}/K_D$ μM (95% CI)	E_{max}	Goodness of Fit		n	
			df	ss	values	studies
$\alpha_4\beta_2$ nAChR[23,24]	3.13 (0.387-21.7)	75.0%	9	3097	11	2
HCN1[25–28]	4.19 (0.748-8.98)	96.6%	11	4426	13	4
$\alpha_1\beta_2\gamma_2$ $GABA_AR$[29–42]	5.44 (2.69-10.6)	91.9%	86	64682	88	13
M_1 mAChR[43]	5.80 (0.015-38.7)	100%	2	555	4	1
Na_v (neuron)[44–53]	8.08 (3.23-16.9)	71.3%	50	23009	52	10
$5\text{-}HT_{3A}$[46,54–57]	10.2 (3.96-23.8)	77.6%	30	16320	32	5
$\alpha_1\beta$ GlyR[58]	22.6 (16.5-30.5)	92.4%	6	74.7	8	1
T-type Ca_v[59–61]	23.7 (15.3-33.7)	100%	17	3243	19	3
$I_{k(dr)}$[62–65]	56.5 (43.0-73.3)	93.2%	13	448	15	4
GluA3 AMPAR[66]	89.3 (12.7-486)	162%	2	223	4	1
GluN1/N2B NMDAR[67]	416 (73.1-652)	100%	2	57.2	4	1
α_2-AR[68]	758	100%	2	774	4	1

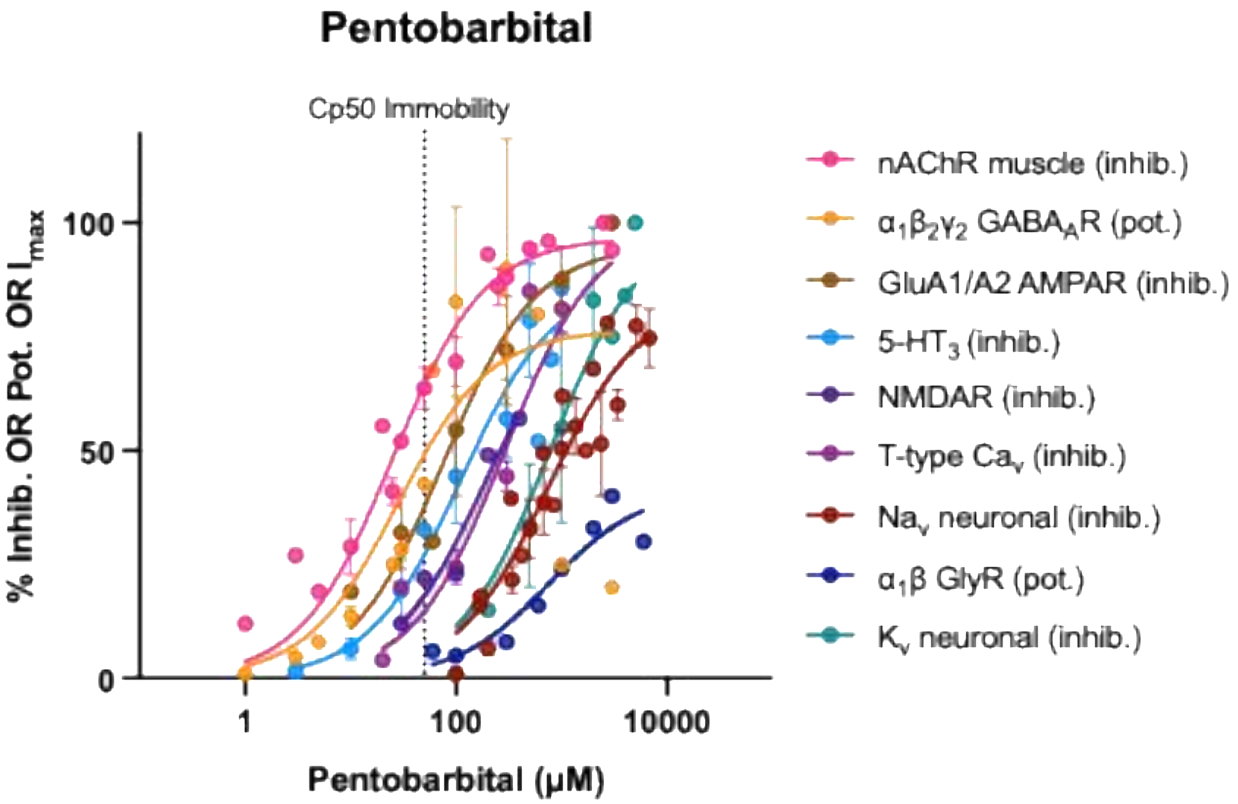

Figure S2

Receptor (subtype)	$EC_{50}/IC_{50}/K_D$ μM (95% CI)	E_{max}	Goodness of Fit		n	
			df	ss	values	studies
nAChR (muscle)[69–72]	25.1 (18.7-33.1)	96.8%	23	1325	25	4
$\alpha_1\beta_2\gamma_2$ $GABA_AR$ [29,30,37,39,73–76]	27.9 (10.8-63.2)	76.5%	28	17378	30	8
GluA1/A2 AMPAR[77,78]	76.4 (35.9-139)	95.0%	10	1356	12	2
$5\text{-}HT_{3A}$[54,56,79,80]	111 (57.0-215)	87.0%	30	6195	32	4
NMDAR[81]	186 (49.6-380)	85.4%	3	86.6	5	1
T-type Ca_v[59–61,82]	291 (218-372)	100%	21	2654	23	4
Na_v (neuron)[83,44,84–90]	739 (529-1020)	83.3%	49	5309	51	9
$\alpha_1\beta$ GlyR[58]	756 (273-1922)	41.4%	6	131	8	1
K_v (neuron)[44,63]	776 (338-1330)	100%	9	2406	11	2

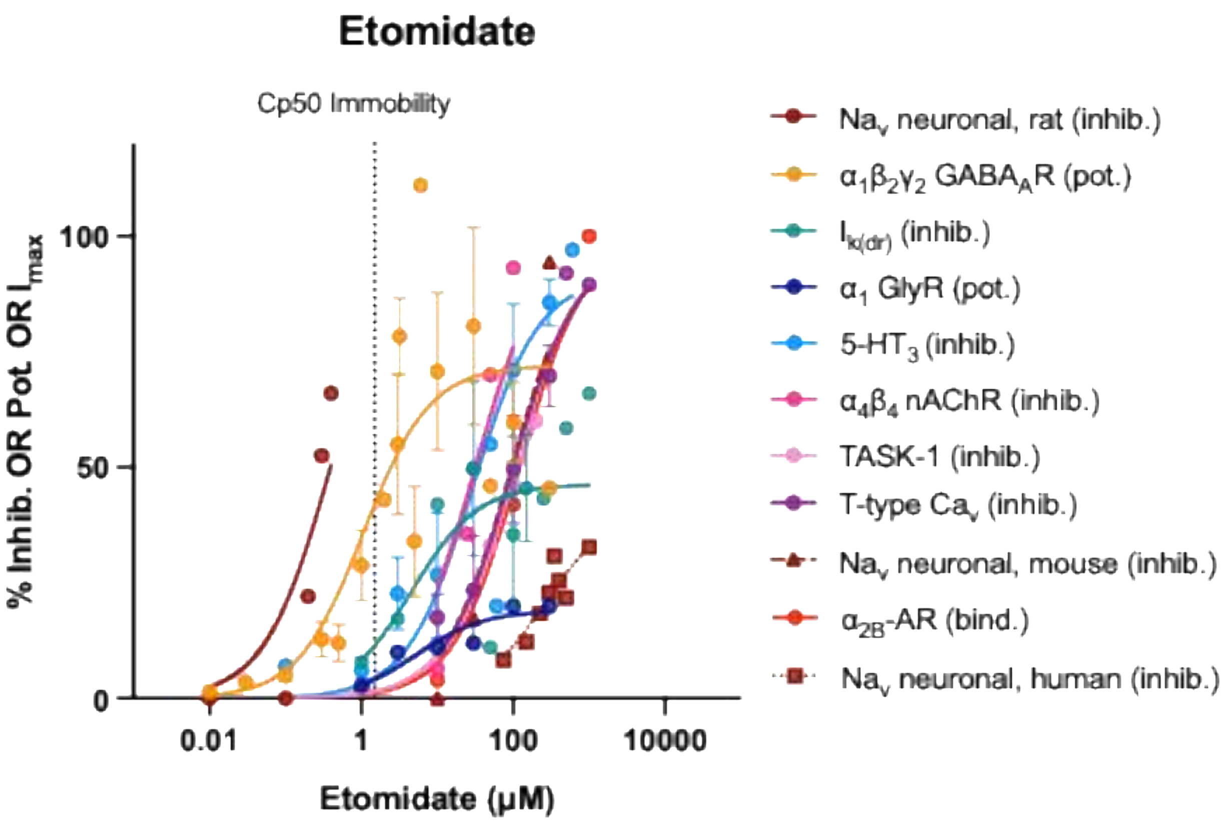

Figure S3

Receptor (subtype)	$EC_{50}/IC_{50}/K_D$ µM (95% CI)	E_{max}	Goodness of Fit		n	
			df	ss	values	studies
$\alpha_1\beta_2\gamma_2$ $GABA_AR$[30,91,58,75,37,92–95,39,96,41,97]	1.08 (0.44-2.31)	72.0%	53	37094	55	13
$I_{k(dr)}$[62,98,99]	4.59 (0.150-152)	46.3%	10	2664	12	3
α_1 GlyR[58]	5.37 (0.791-33.0)	19.0%	4	34.7	6	1
$5\text{-}HT_{3A}$[46,56,94,100]	29.5 (6.99-70.3)	91.4%	20	8793	22	4
$\alpha_4\beta_4$ nAChR[101]	31.8	100%	2	757	4	1
TASK-1[102]	77.3 (19.8-154)	82.8%	1	0.80	3	1
T-type Ca_v[59–61]	93.9 (30.9-172)	97.2%	13	3909	15	3
α_{2B} AR	121 (22.1-432)	100%	2	142	4	1
Na_v (neuron, rat)[103] **	0.393	100%	3	880	5	1
Na_v (neuron, mouse)[103] **	95.1	100%	2	505	4	1
Na_v (neuron, human)[103] **	264 (82.3-754)	41.3%	6	90.3	8	1
TREK-1[104]	60.2% potentiation at 10µM ETO				1	1

*** The Na_v data were clearly in three groups – see main text for discussion.*

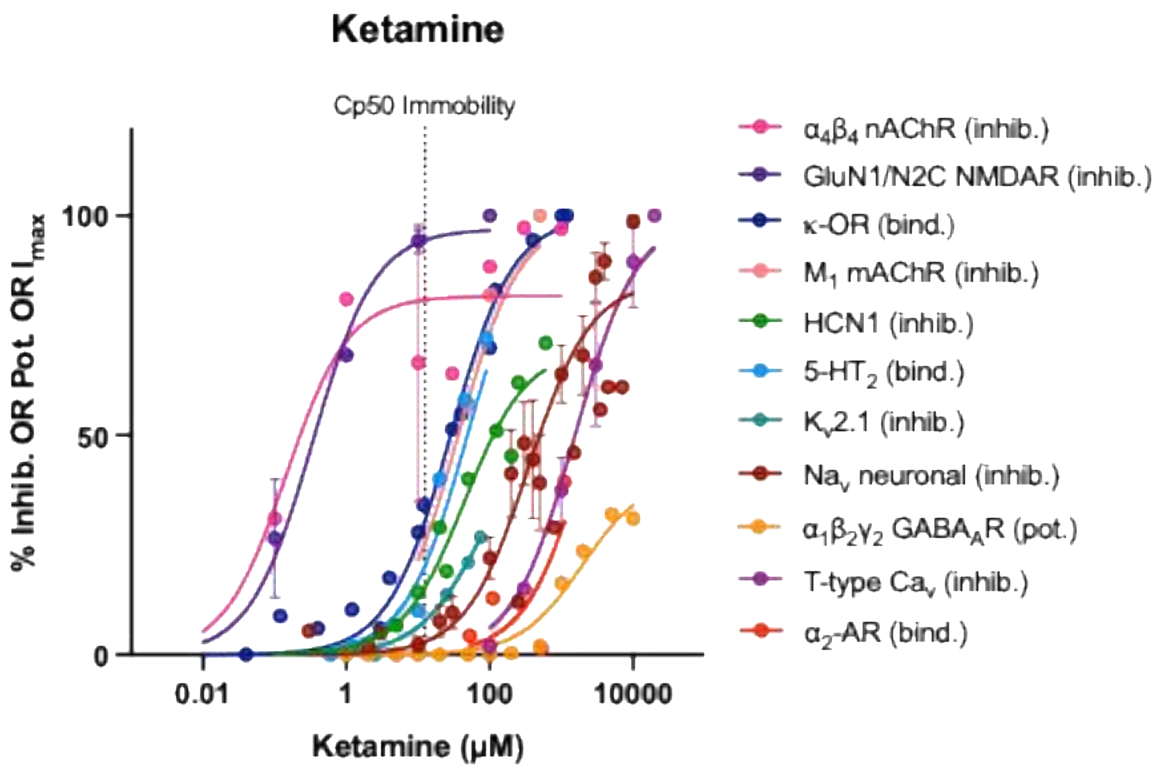

Figure S4

Receptor (subtype)	$EC_{50}/IC_{50}/K_D$ μM (95% CI)	E_{max}	Goodness of Fit		n	
			df	ss	values	studies
$\alpha_4\beta_4$ nAChR[105,101]	0.144	81.7%	6	3302	8	2
GluN1/N2C NMDAR[106,107]	0.338 (0.132-0.757)	97.0%	5	465	7	2
κ-OR[108,109]	28.5 (22.7-34.0)	100%	18	344	20	2
M_1 mAChR[110]	36.5 (4.55-125)	100%	2	256	4	1
HCN1[111,112]	44.4 (23.7-86.4)	69.7%	8	287	10	2
5-HT_2[113]	48.9 (15.9-66.2)	100%	6	435	8	1
K_v2.1[114]	71.7 (9.79-239)	52.1%	1	0.24	3	1
Na_v (neuron)[44,115,46,116–121]	348 (206-571)	85.2%	71	22831	73	9
$\alpha_1\beta_2\gamma_2$ $GABA_AR$[101]	1951 (1104-3567)	40.7%	11	88.4	13	1
T-type Ca_v[59,60]	1551 (815-2498)	100%	7	818	9	2
α_2-AR[68]	2493 (221-4606)	100%	22	5961	24	1

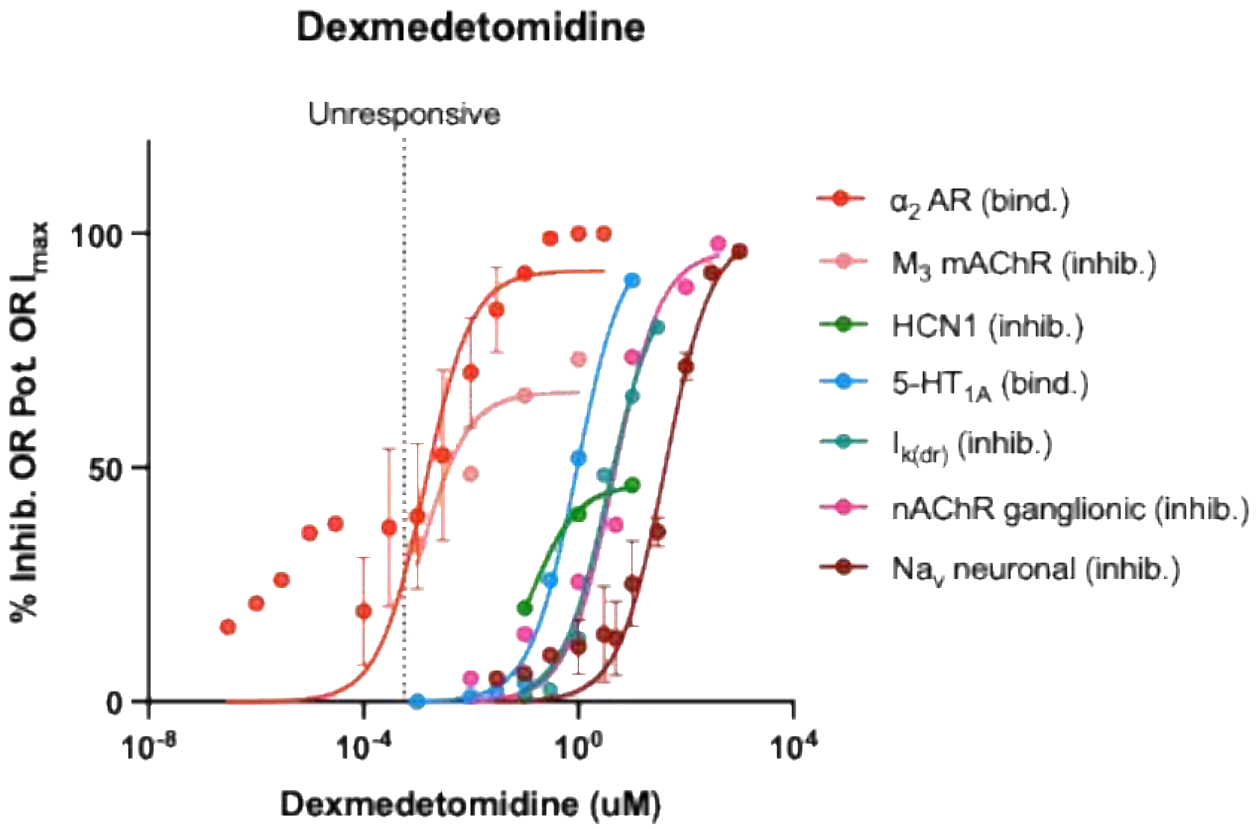

Figure S5

Receptor (subtype)	$EC_{50}/IC_{50}/K_D$ μM (95% CI)	E_{max}	Goodness of Fit		n	
			df	ss	values	studies
α_{2A} AR[122–124]	0.0014 (0.0004-0.004)	92.1%	28	12522	30	3
M_3 mAChR[125]	0.001	66.1%	2	170	4	1
HCN1[126]	0.135 (0.026-0.531)	47.5%	1	1.10	3	1
5-HT_{1A}[124]	0.928 (0.692-1.18)	98.7%	5	36.9	7	1
$I_{k(dr)}$ [127]	3.54 (1.79-6.66)	90.0%	4	114	6	1
nAChR (ganglion)[128]	4.62 (1.52-10.3)	96.3%	5	476	7	1
Na_v (neuron) [129,127,130,131,128,132]	41.3 (25.7-53.9)	100%	25	2580	27	6

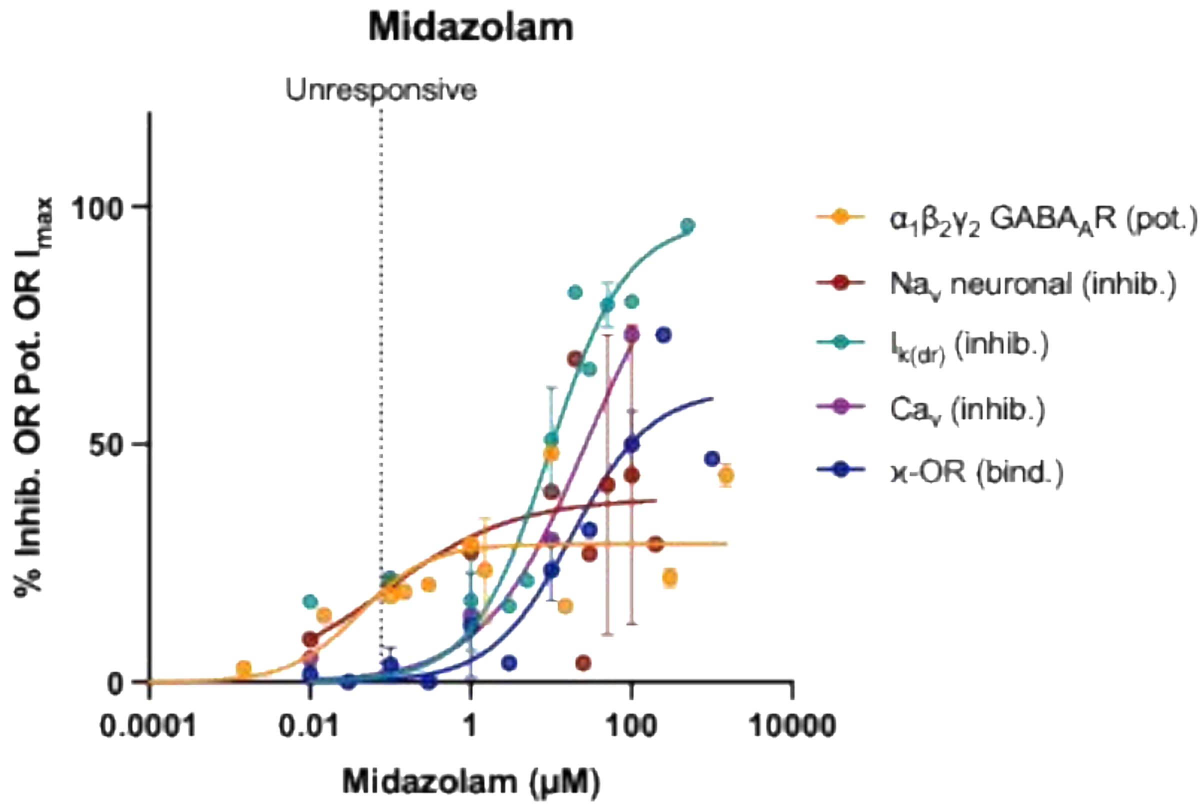

Figure S6

Receptor (subtype)	$EC_{50}/IC_{50}/K_D$ µM (95% CI)	E_{max}	Goodness of Fit		n	
			df	ss	values	studies
$\alpha_1\beta_2\gamma_2$ $GABA_AR$[133,134]	0.041 (0.001-0.649)	29.1%	20	3310	22	2
Na_v (neuron)[135–137]	0.074	37.0%	10	6392	12	3
nAChR (muscle)[138]	0.143 (0.034-0.580)	58.0%	4	219	6	1
$I_{k(dr)}$[63,136,137]	9.03 (4.00-16.7)	94.4%	12	1952	14	3
Ca_v[136]	16.0 (0.594-99.4)	84.2%	2	112	4	1
κ-OR[139,140]	16.8 (6.01-42.8)	59.6%	14	1133	16	2

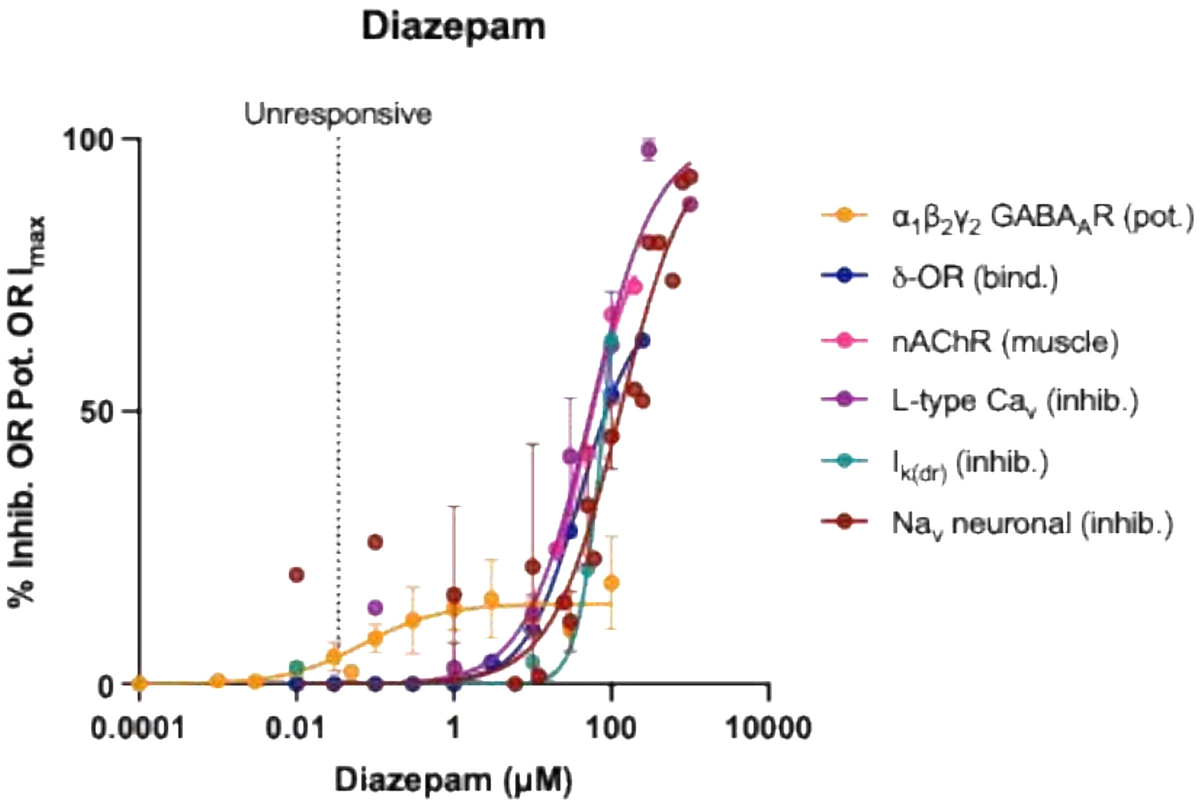

Figure S7

Receptor (subtype)	$EC_{50}/IC_{50}/K_D$ μM (95% CI)	E_{max}	Goodness of Fit		n	
			df	ss	values	studies
$\alpha_1\beta_2\gamma_2$ $GABA_AR$[141–144,74,145–147,41]	0.070 (0.013-0.264)	14.5%	38	1825	40	6
δ-OR[140]	54.0 (44.3-66.0)	78.2%	8	13.8	10	1
nAChR (muscle)[148]	57.0 (13.0-103)	97.1%	2	52.9	4	1
L-type Ca_v[149–151]	52.0 (27.8-78.0)	100%	15	2418	17	3
$I_{k(dr)}$[136]	99.8 (9.83-243)	100%	4	418	6	1
Na_v (neuron)[152–154,136]	137 (72.1-181)	100%	29	5936	31	4

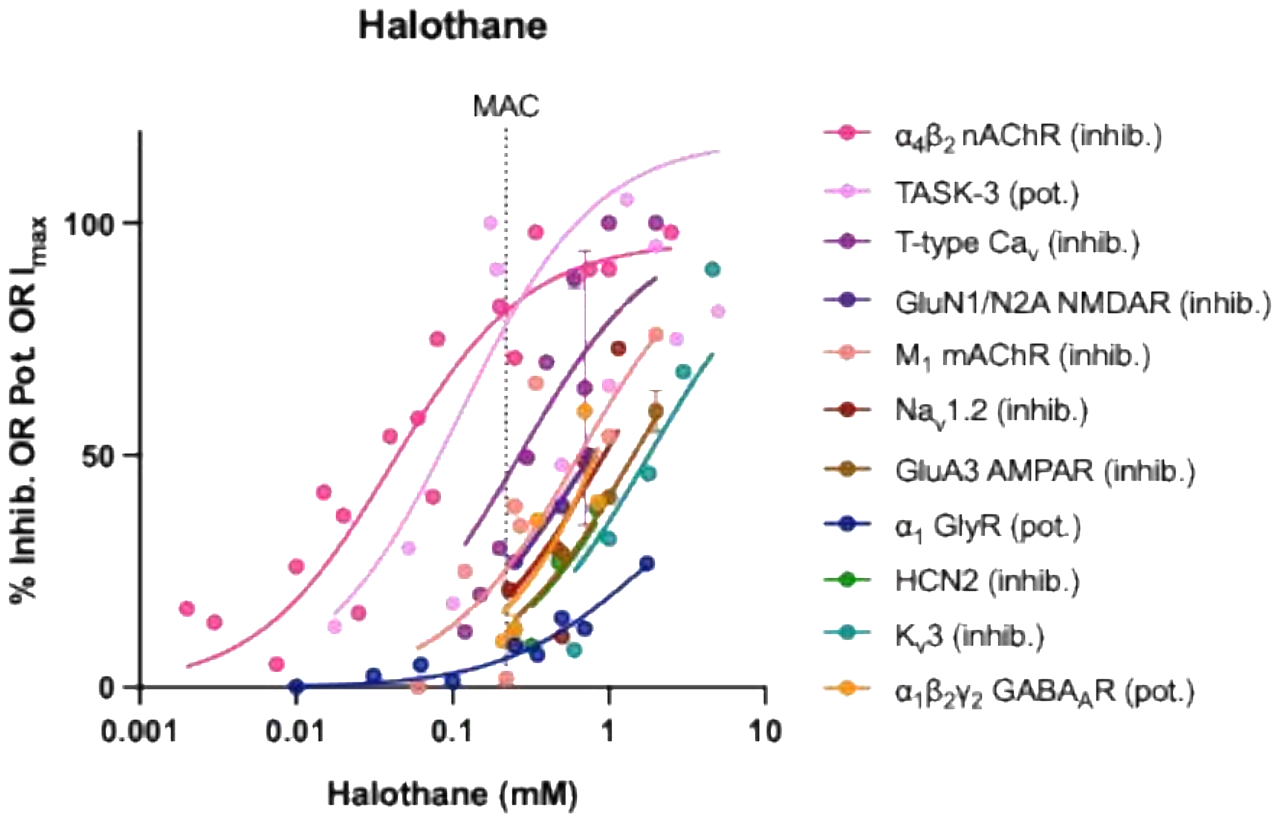

Figure S8

Receptor (subtype)	$EC_{50}/IC_{50}/K_D$ mM (95% CI)	E_{max}	Goodness of Fit		n	
			df	ss	values	studies
$\alpha_4\beta_2$ nAChR[24,155,156]	0.041 (0.023-0.066)	96.1%	15	2000	17	3
TASK-3[157–159]	0.113 (0.003-0.834)	118%	11	27022	13	3
T-type Ca_v[59,160,161]	0.270	100%	10	4067	12	3
GluN1/N2A NMDAR[162]	0.594	88.3%	1	3.08	3	1
M_1 mAChR[163,164]	0.649 (0.081-1.67)	100%	7	2766	9	2
Na_v1.2[50,165]	0.930	100%	3	940	5	2
GluA3 AMPAR[166,167]	1.38 (0.495-1.84)	100%	3	55.6	5	2
α_1 GlyR^[168–170]	1.50 (0.529-8.56)	48.6%	7	39.6	9	3
HCN2[171]	1.51	100%	1	90.1	3	1
5-HT_3[172–176]	1.59 (0.536-5.60)	53.5%	18	1245	20	5
K_v3[177]	1.81 (0.193-4.62)	100%	3	678	5	1
$\alpha_1\beta_2\gamma_2$ $GABA_A$R[178–182]	2.16	182%	4	612	6	5

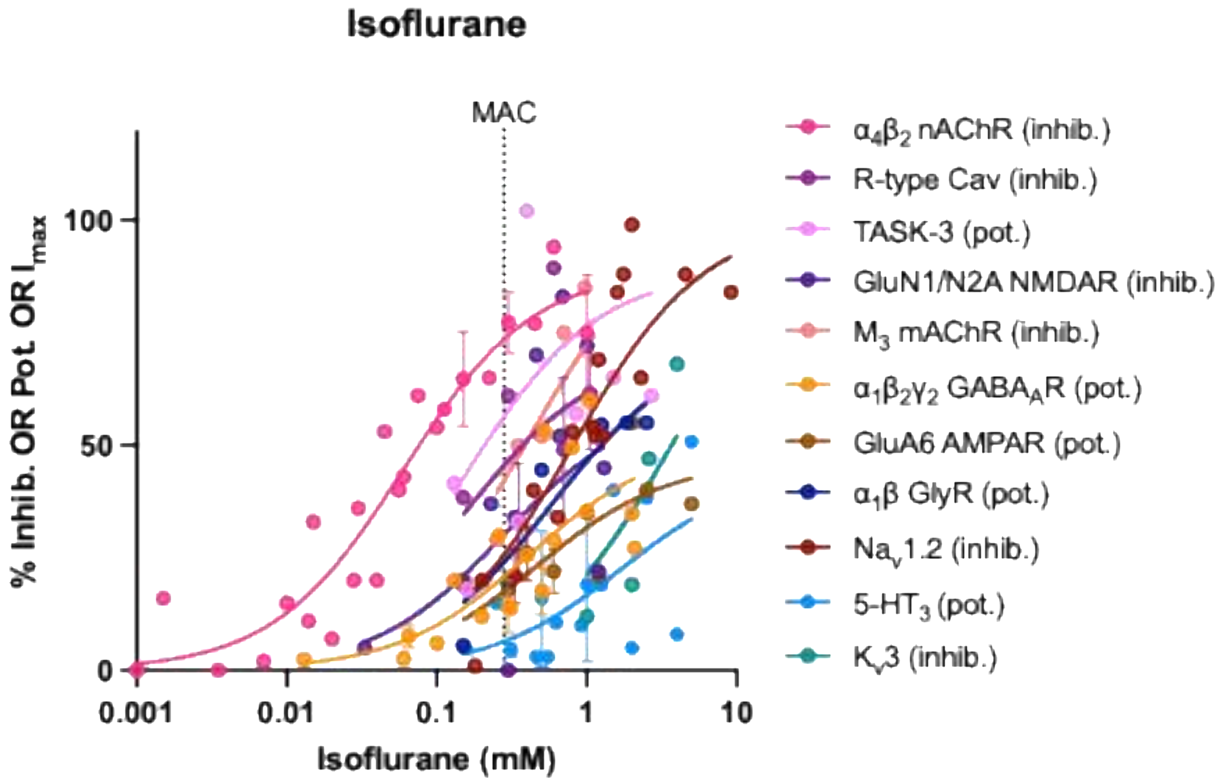

Figure S9

Receptor (subtype)	$EC_{50}/IC_{50}/K_D$ mM (95% CI)	E_{max}	Goodness of Fit		n	
			df	ss	values	studies
$\alpha_4\beta_2$ nAChR[23,24,183–185,156]	0.060 (0.038-0.094)	89.2%	27	3458	29	6
R-type Ca_v[186,187]	0.159	71.2%	7	3155	9	2
TASK-3[157–159]	0.162	88.8%	6	8132	8	2
GluN1/N2A NMDAR [162,184,188–191]	0.282	60.3%	11	6896	13	6
M_3 mAChR[192,193]	0.384 (0.009-0.720)	100%	3	424	5	2
$\alpha_1\beta_2\gamma_2$ $GABA_AR$[181,194–198]	0.405 (0.140-1.06)	50.9%	21	2483	23	6
GluA6 AMPAR[166,167]	0.471 (0.019-2.22)	46.7%	7	657	9	2
$\alpha_1\beta$ GlyR[199]	0.571 (0.154-2.57)	72.9%	4	268	6	1
$Na_v1.2$[165,200–202]	0.810 (0.542-1.07)	100%	17	2646	19	4
$5\text{-}HT_3$[172–176]	1.69	45.0%	18	3073	20	5
K_v3[177]	3.70	100%	2	635	4	1
I_h[203]	45% inhibition at 0.9mM ISO				1	1

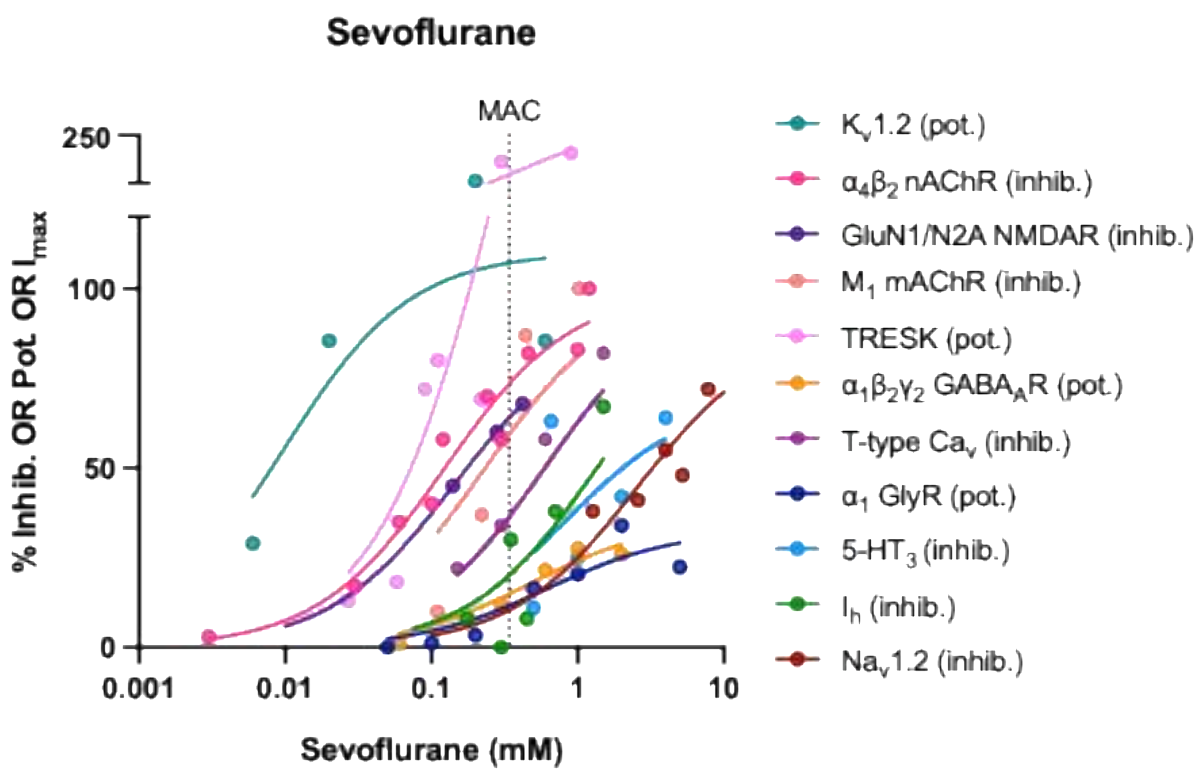

Figure S10

Receptor (subtype)	$EC_{50}/IC_{50}/K_D$ mM (95% CI)	E_{max}	Goodness of Fit		n	
			df	ss	values	studies
K_v1.2[204]	0.0097	110%	6	32096	8	1
$\alpha_4\beta_2$ nAChR[24,156]	0.123 (0.074-0.164)	100%	8	425	10	2
GluN1/N2A NMDAR[188]	0.144 (0.104-0.192)	91.2%	1	0.06	3	1
M_1 mAChR[192]	0.233	100%	2	1434	4	1
TRESK[205]	0.348 (0.085-2.16)	290%	5	4730	7	1
$\alpha_1\beta_2\gamma_2$ $GABA_A$R[195]	0.470 (0.093-2.37)	35.5%	3	34.8	5	1
T-type Ca_v[161]	0.593 (0.023-1.25)	100%	2	170	4	1
α_1 GlyR[206]	0.615 (0.128-2.81)	32.7%	5	168	7	1
5-HT_3[174,176]	0.819	70.2%	3	1525	5	2
I_h[207,208]	1.35 (0.276-2.79)	100%	6	1143	8	2
Na_v1.2[200]	2.57 (0.782-4.91)	89.5%	5	289	7	1

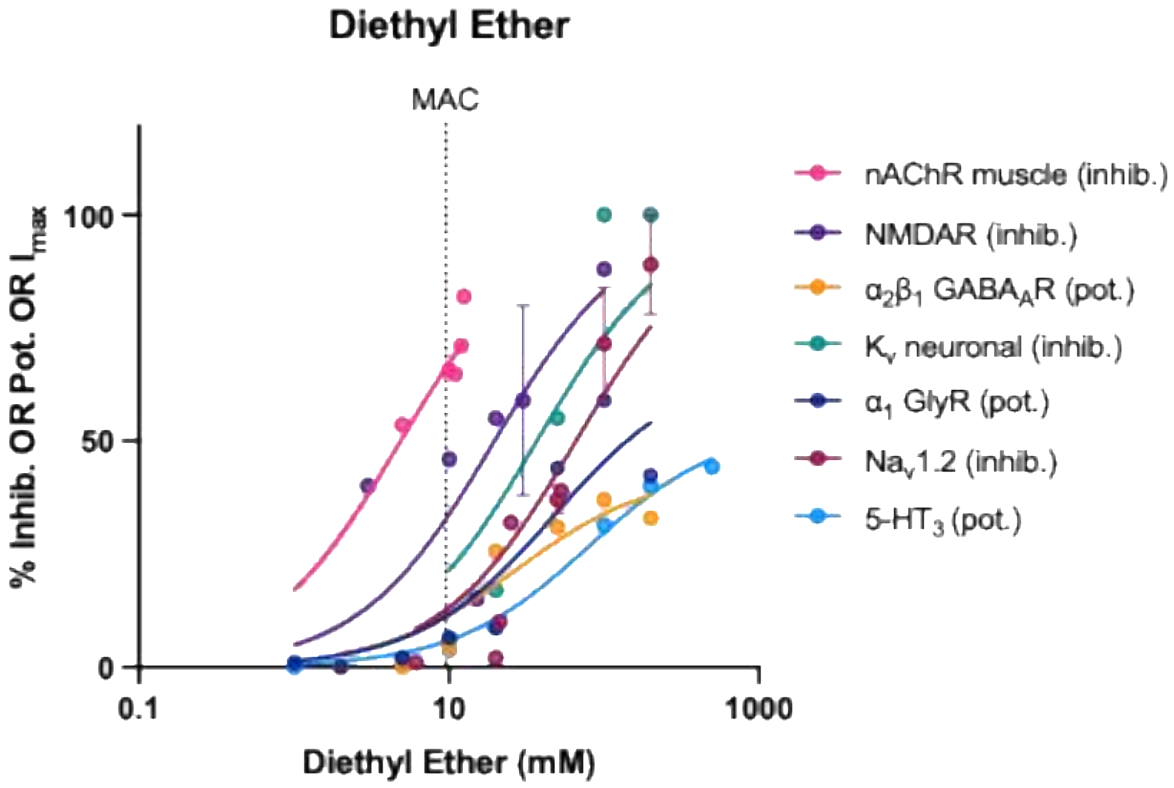

Figure S11

Receptor (subtype)	$EC_{50}/IC_{50}/K_D$ mM (95% CI)	E_{max}	Goodness of Fit		n	
			df	ss	values	studies
nAChR (muscle)[209]	4.87	100%	3	132	5	1
NMDAR[210,211]	19.4	99.4%	5	2593	7	2
$\alpha_2\beta_1$ $GABA_AR$[206]	27.3 (7.55-110)	43.1%	5	206	7	1
K_v (neuron)[44]	36.9 (1.90-159)	100%	3	1540	5	1
α_1 GlyR[206]	47.3 (11.6-250)	66.9%	6	594	8	1
$Na_v1.2$[212,44,200]	65.9 (35.2-105)	100%	9	2045	11	3
$5\text{-}HT_3$[213]	80.0 (52.2-124)	53.4%	5	19.6	7	1
TREK-1[214]	80% potentiation at 0.6mM ETHER				1	1
AMPAR[169]	20% inhibition at 15mM ETHER; 57% at 20mM				2	1

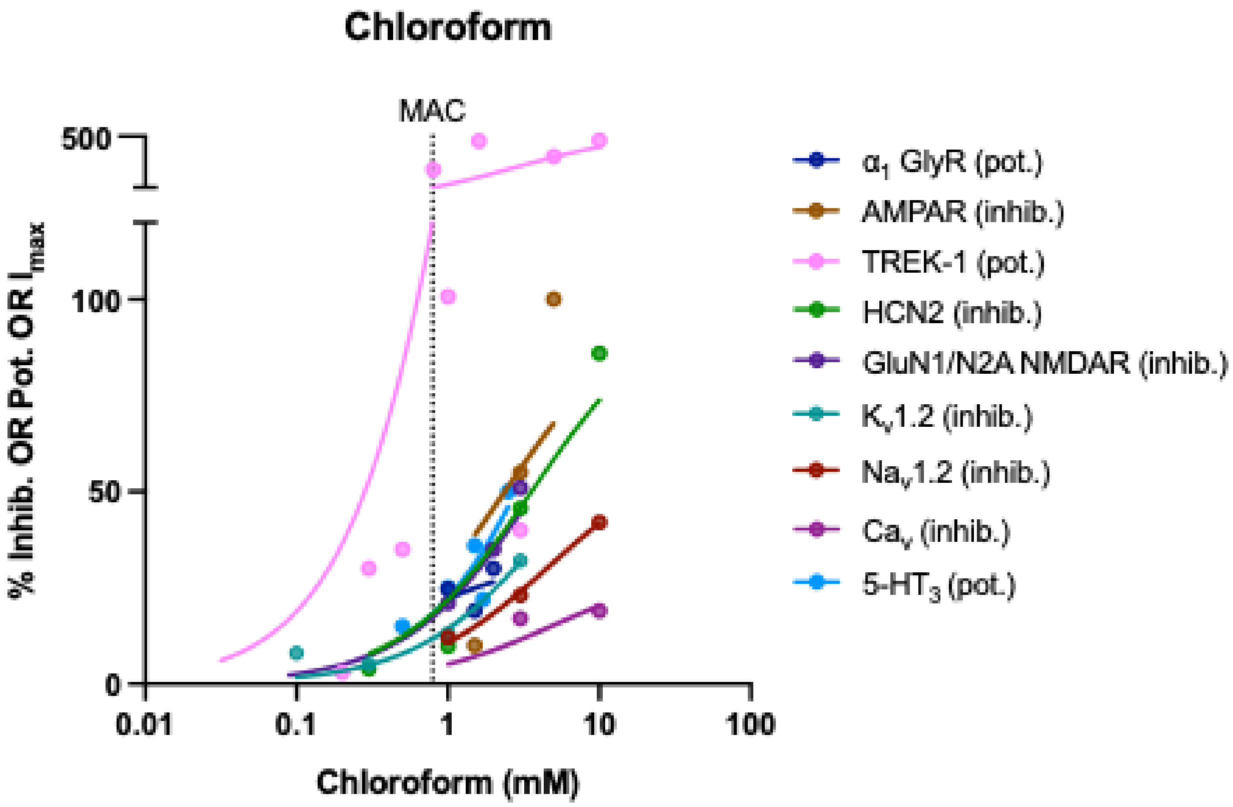

Figure S12

Receptor (subtype)	$EC_{50}/IC_{50}/K_D$ mM (95% CI)	E_{max}	Goodness of Fit		n	
			df	ss	values	studies
α1 GlyR[215–217]	0.480	32.9%	1	53.2	3	3
AMPAR[210]	2.37	100%	1	1862	3	1
TREK-1[104,214,218,219]	2.74	536%	9	161090	11	4
HCN2[220]	3.56 (0.167-16.1)	100%	2	312	4	1
GluN1/N2A[162]	3.77	100%	1	45.0	3	1
Kv1.2[221]	4.63 (0.006-17.5)	80.8%	2	41.5	4	1
Na_v1.2[222]	4.68 (0.116-24.8)	61.3%	1	2.41	3	1
Ca_v[222]	4.88	30.2%	1	82.9	3	1
5-HT_3[175,176]	6.39	164%	3	211	5	2
α1β2γ2 $GABA_AR$[179,181]	8% increase at 0.9mM CHLOR				2	2

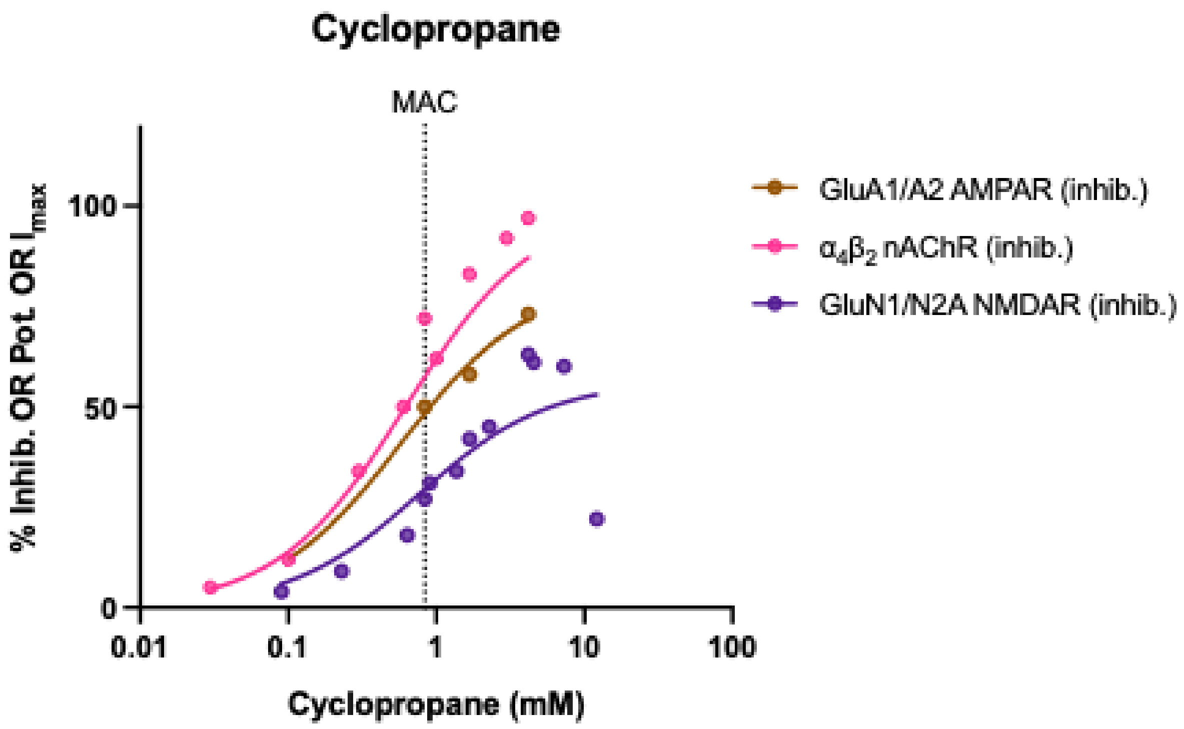

Figure S13

Receptor (subtype)	$EC_{50}/IC_{50}/K_D$ mM (95% CI)	E_{max}	Goodness of Fit		n	
			df	ss	values	studies
GluA1/A2 AMPAR[169]	0.578	81.6%	1	11.7	3	1
$\alpha_4\beta_2$ nAChR[223,169]	0.622 (0.262-0.741)	100%	7	501	9	2
GluN1/N2A NMDAR[169,162,191]	0.764 (0.180-2.20)	56.4%	10	1549	12	3
TREK-1^[224]	0% potentiation at 0.09mM CYCLO; 14% at 0.46mM; 26% at 0.91mM; 113% at 3.65mM; 189% at 5.48mM; 277% at 7.3mM				6	1
Na_v1.2[165]	20% inhibition at 3.29 mM CYCLO				1	1
$\alpha_1\beta_2\gamma_2$ $GABA_AR$^[225]	0.1% increase at 0.82mM CYCLO; 1.6% at 2.5mM; 5.0% at 5.7mM; 10% at 9.8mM				4	1

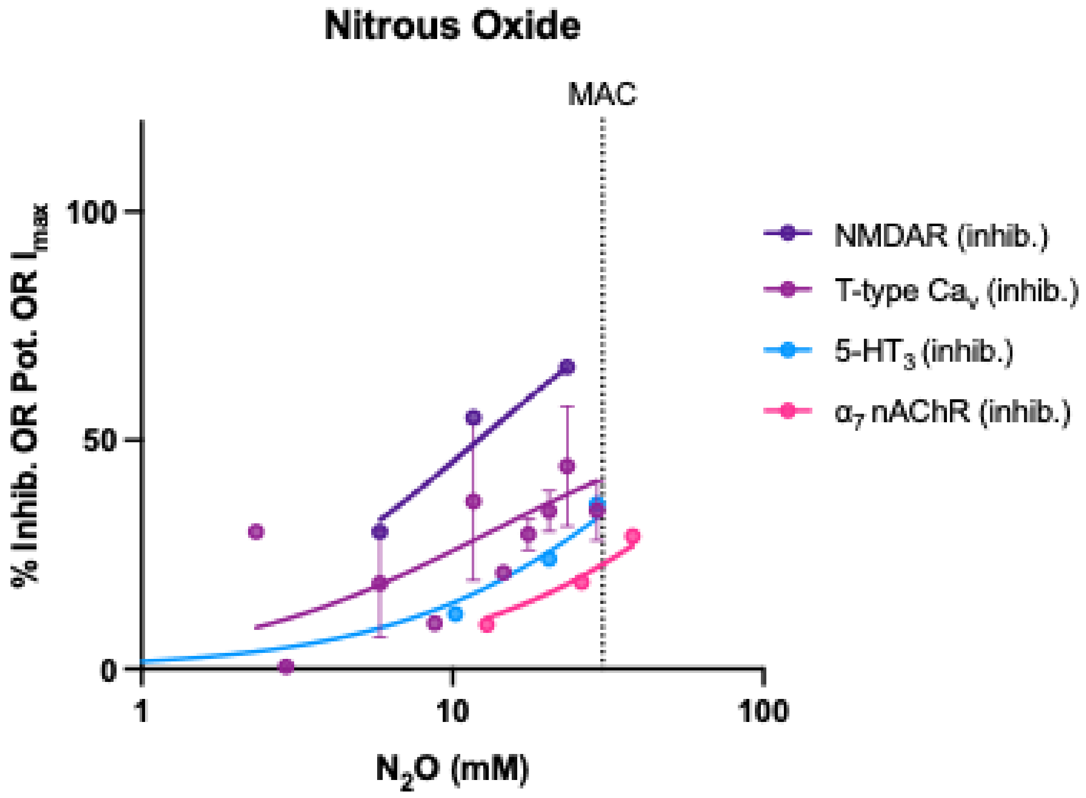

Figure S14

Receptor (subtype)	$EC_{50}/IC_{50}/K_D$ mM (95% CI)	E_{max}	Goodness of Fit		n	
			df	ss	values	studies
NMDAR[226]	12.1	100%	1	41.2	3	1
T-type Ca_v[227,61,228]	13.0 (0.755-53.3)	59.5%	19	6310	21	3
$5\text{-}HT_3$[174]	59.2	100%	1	19.0	3	1
α_7 nAChR[229]	102.6	100%	1	7.53	3	1
GluA1/A2 AMPAR[184]	20% inhibition at 12.2mM N2O				1	1
TREK-1^[224]	3% potentiation at 1.46mM N2O; 6% at 5.84mM; 10% at 11.7mM; 13% at 17.5mM; 26% at 23.4mM				5	1
$\alpha_1\beta_2\gamma_2$ $GABA_AR$[230]	11% increase at 29.2mM N2O				1	1
α_1 GlyR^^[168,184]	1.7% increase at 12.2mM N2O; 3.8% at 22.5mM; 5.7% at 113mM; 25% at 225mM; 69.6% at 338mM				5	2

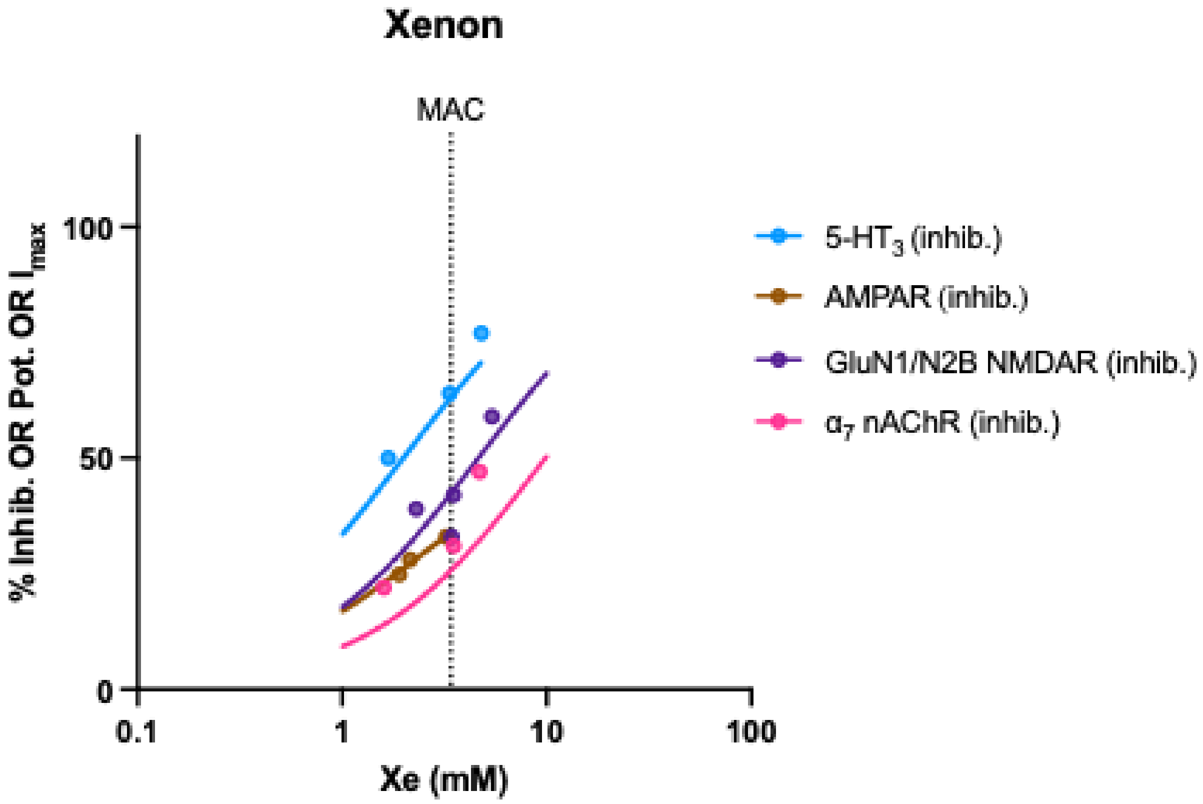

Figure S15

Receptor (subtype)	$EC_{50}/IC_{50}/K_D$ mM (95% CI)	E_{max}	Goodness of Fit		n	
			df	ss	values	studies
AMPAR[231,232]	2.48	58.7%	1	1.02	3	2
5-HT_3[174]	1.99	100%	1	59.7	3	1
GluN1/N2B NMDAR[4,190,233]	4.64	100%	2	154	4	3
α_7 nAChR[229]	9.86	100%	1	304	3	1
TREK-1[224,234,235]	6% potentiation at 0.22mM XE; 10% at 0.86mM; 14% at 1.72mM; 26% at 2.58mM; 35.7% at 3.44mM				7	3
I_h[236]	33.4% inhibition at 1.9mM XE				1	1
$\alpha_1\beta_2\gamma_2$ $GABA_AR$[230]	6.2% increase at 3.9mM XE				1	1

Individual Receptors

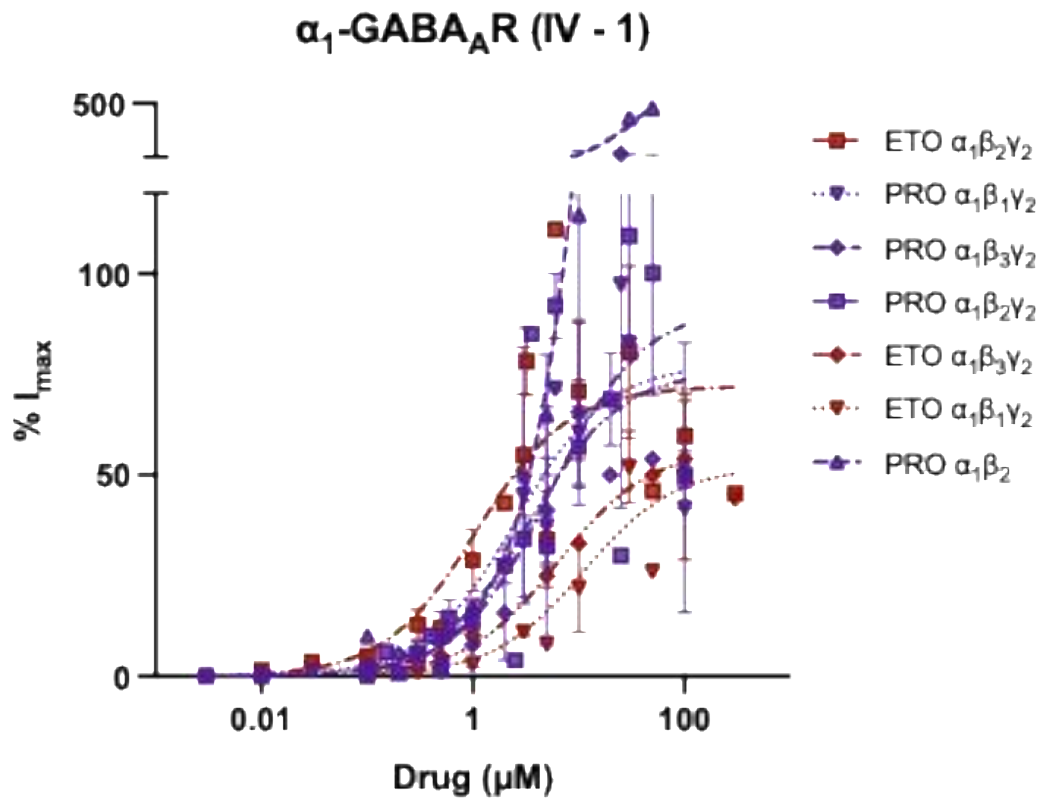

Figure S16A

Drug	Subtype	EC_{50} μM (95% CI)	E_{max}	Goodness of Fit		n	
				df	ss	values	studies
ETO	α1β2γ2[30,91,58,75,37,92–95,39,96,41,97]	1.08 (0.44-2.31)	72.0%	53	37094	55	13
	α1β3γ2[91]	6.32 (4.69-8.45)	56.5%	5	13.8	7	1
	α1β1γ2[30,91,237]	11.2 (3.38-34.6)	52.2%	14	2053	16	3
	α1β1ε[237]	2% increase at 3μM ETO; 44% at 30μM				2	1
	α1β2ε[237]	4% increase at 0.3μM ETO; 54% at 3μM				2	1
	α1β1[237]	18% increase at 3μM ETO; 43% at 30μM				2	1
	α1β2[237]	16% increase at 3μM ETO; 86% at 30μM				2	1
PRO	α1β1γ2[30,33,58,237–239]	2.58 (0.72-7.30)	77.6%	27	18358	29	6
	α1β3γ2[33,240–243]	3.38 (0.85-10.3)	76.0%	20	14973	22	5
	α1β2γ2[29–42]	5.44 (2.69-10.6)	91.9%	86	64682	88	13
	α1β2[31,244]	86.72	1333%	9	10172	11	2
	α1β1[237]	94.4% increase at 10μM PRO				1	1
	α1β1ε[237]	55.4% increase at 10μM PRO				1	1

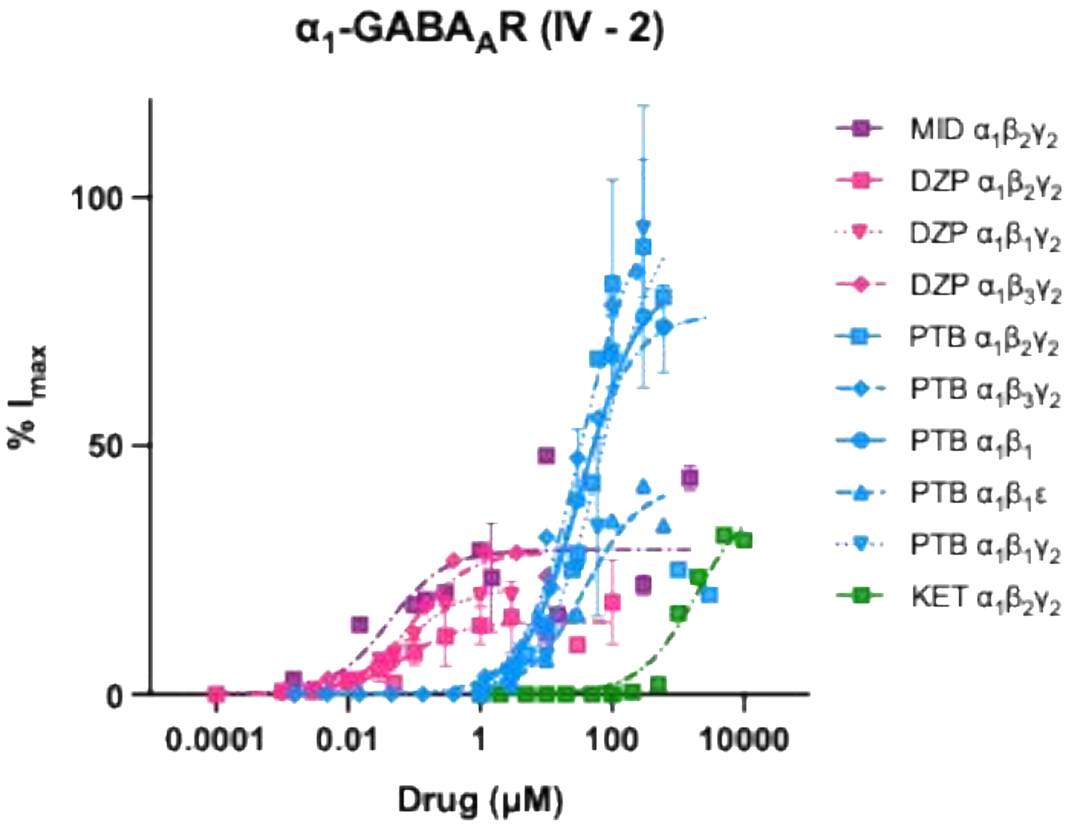

Figure S16B

Drug	Subtype	EC50 μM (95% CI)	Emax	Goodness of Fit		n	
				df	ss	values	studies
MID	α1β2γ2[133,134]	0.041 (0.001-0.649)	29.1%	20	3310	22	2
DZP	α1β2γ2[141,144,74,146,147,41]	0.070 (0.013-0.264)	14.5%	38	1825	40	6
	α1β1γ2[142,143,145]	0.074 (0.06-0.09)	21.2%	8	3.31	10	3
	α1β3γ2[245]	0.094 (0.048-0.176)	28.6%	7	51.9	9	1
PTB	α1β2γ2[29,30,37,39,73–76]	27.9 (10.8-63.2)	76.5%	28	17378	30	8
	α1β3γ2[245–247]	33.7 (23.6-47.9)	99.4%	14	298	16	3
	α1β1[237]	35.1 (21.4-57.2)	83.4%	5	106	7	1
	α1β1ε[237]	42.5 (16.1-90.9)	42.5%	5	84.9	7	1
	α1β1γ2[30,58,145,237,239]	69.8 (35.3-132)	97.9%	31	11080	33	5
KET	α1β2γ2[101]	1951 (1104-3567)	40.7%	11	88.4	13	1

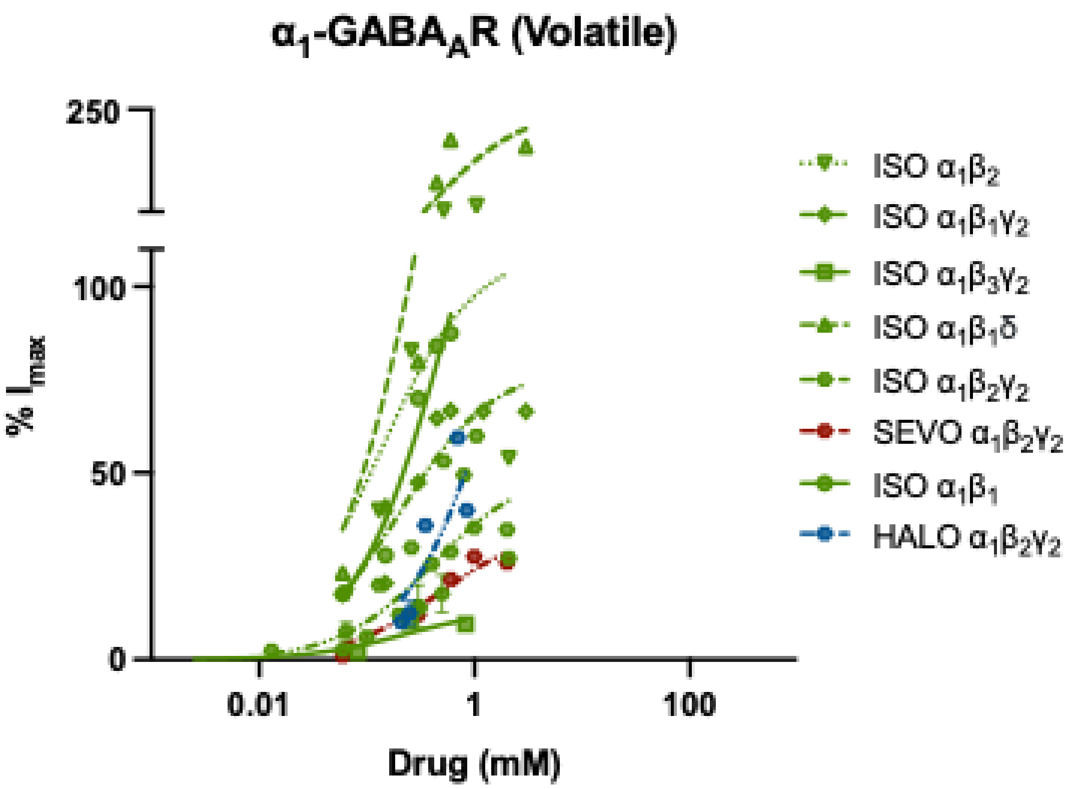

Figure S17

Drug	Subtype	EC50 mM (95% CI)	Emax	Goodness of Fit		n	
				df	ss	values	studies
ISO	α1β2[196]	0.132	110%	4	5198	6	1
	α1β1γ2[248]	0.203 (0.071-0.473)	78.8%	5	394	7	1
	α1β3γ2[249]	0.224	13.4%	3	19.2	5	1
	α1β1δ[248]	0.377 (0.062-1.50)	254%	4	6157	6	1
	α1β2γ2[181,194–198]	0.405 (0.140-1.06)	50.9%	21	2483	23	6
	α1β1[248]	0.576	182%	3	197	5	1
SEVO	α1β2γ2[195]	0.470 (0.093-2.37)	35.5%	3	34.8	5	1
HALO	α1β2γ2[178–182]	2.16	182%	4	612	6	5
CHLOR	α1β2γ2[179,181]	8% increase at 0.9mM CHLOR				2	2
CYCLO	α1β2γ2^[225]	0.1% increase at 0.82mM CYCLO; 1.6% at 2.5mM; 5.0% at 5.7mM; 10% at 9.8mM				4	1
N2O	α1β2γ2[230]	11% increase at 29.2mM N2O				1	1
XE	α1β2γ2[230]	6.2% increase at 3.9mM XE				1	1

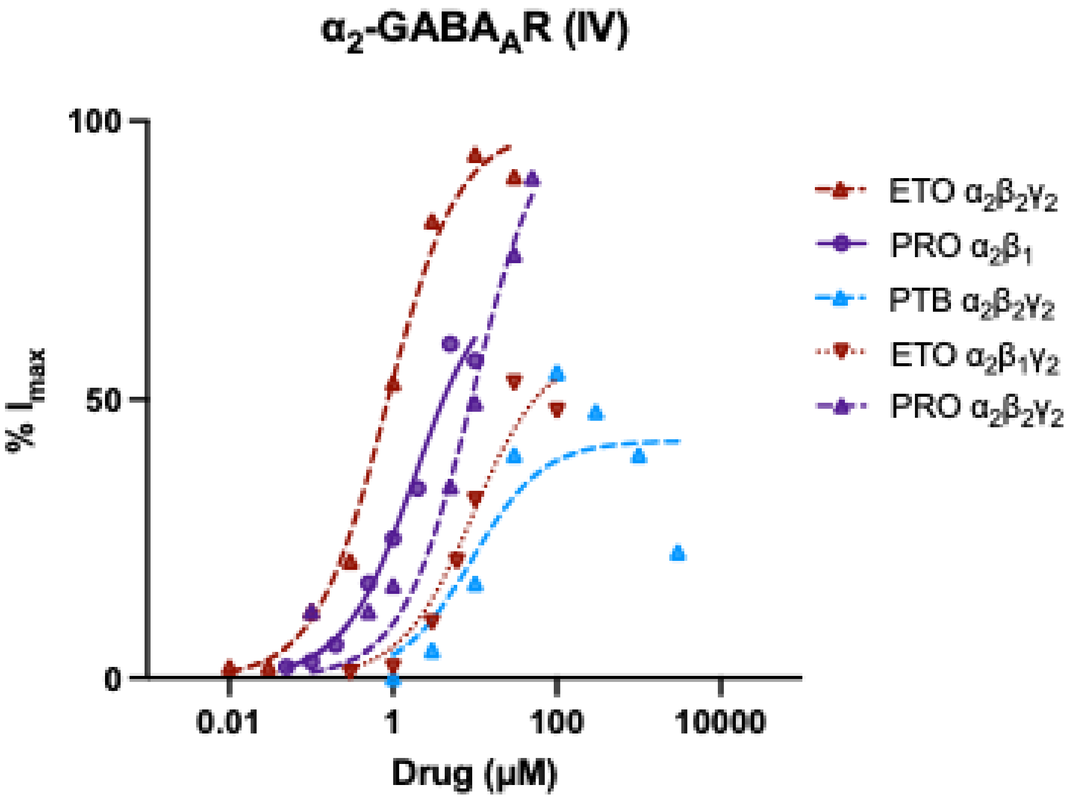

Figure S18

Drug	Subtype	EC_{50} μM (95% CI)	E_{max}	Goodness of Fit		n	
				df	ss	values	studies
ETO	α2β2γ2[30]	0.840 (0.597-1.17)	98.4%	6	98.6	8	1
	α2β1γ2[30]	9.47 (4.32-19.9)	58.8%	5	139	7	1
	α2β1[250]	23% increase at 10μM ETO				1	1
PRO	α2β1[250]	1.84 (1.08-3.15)	72.2%	6	85.3	8	1
	α2β2γ2[31]	9.64 (3.73-25.1)	103%	5	241	7	1
PTB	α2β2γ2[73]	9.23 (1.26-44.1)	42.6%	6	820	8	1
DZP	α2β3γ2[251]	17% increase at 1μM DZP				1	1

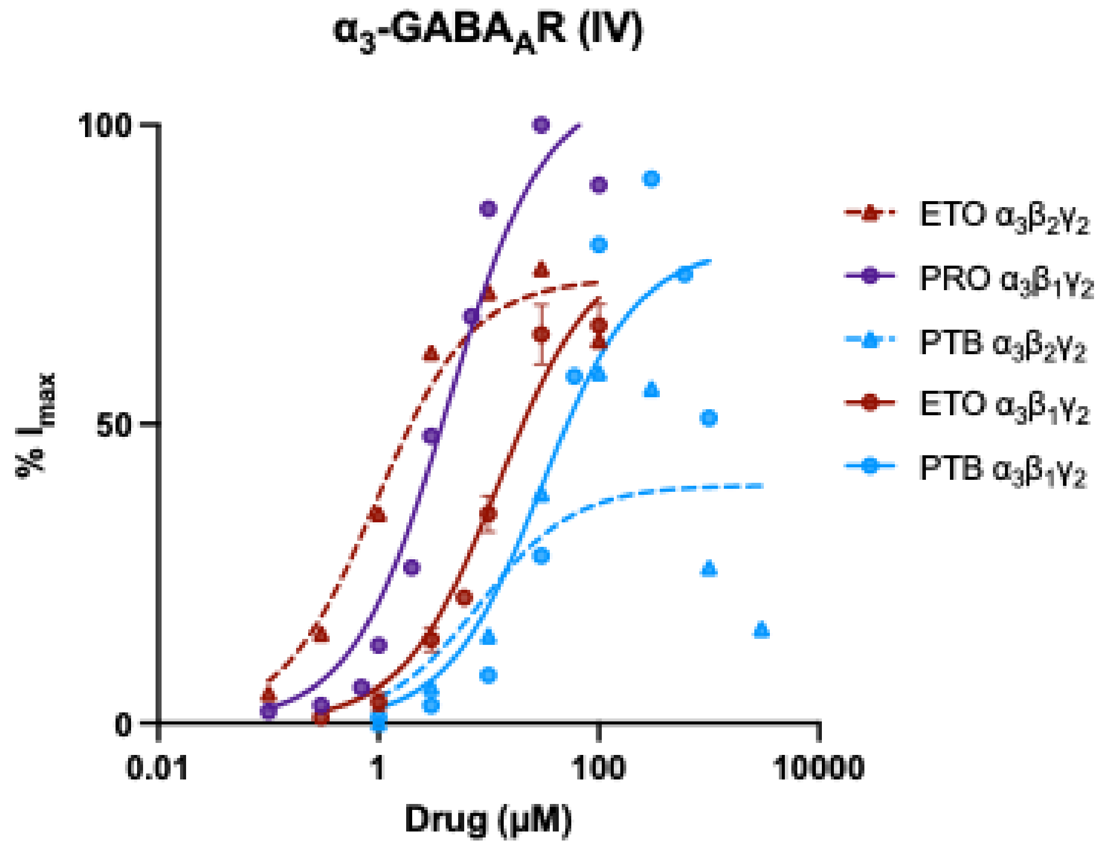

Figure S19

Drug	Subtype	EC_{50} μM (95% CI)	E_{max}	Goodness of Fit		n	
				df	ss	values	studies
ETO	α3β2γ2[30]	0.960 (0.502-1.77)	74.4%	5	178	7	1
	α3β1γ2[30,252]	12.2 (8.13-18.4)	79.8%	11	335	13	2
PRO	α3β1γ2[252]	4.28 (2.65-6.80)	106%	8	555	10	1
PTB	α3β2γ2[73]	8.31 (0.046-78.1)	39.8%	6	1670	8	1
	α3β1γ2[252]	30.6 (7.80-93.0)	79.6%	7	1716	9	1

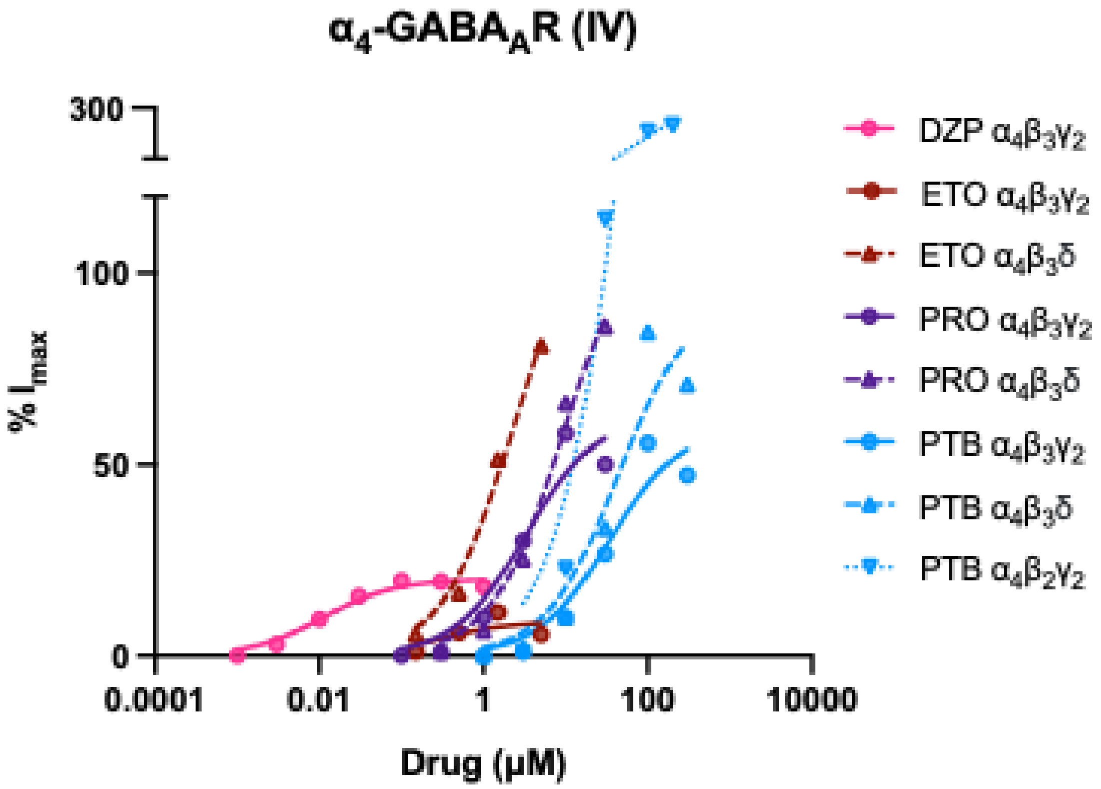

Figure S20

Drug	Subtype	EC_{50} μM (95% CI)	E_{max}	Goodness of Fit		n	
				df	ss	values	studies
DZP	α4β3γ2[253]	0.011 (0.006-0.019)	20.1%	5	11.1	7	1
	α4β2γ2^[74]	0% increase at 0.01μM DZP; 0.1% at 0.1μM; 0.1% at 1μM; 0.5% at 10μM; 8.2% at 100μM				5	1
ETO	α4β3γ2[254]	0.296	9.0%	2	27.7	4	1
	α4β3δ[254]	2.33 (0.715-16.1)	121%	2	45.9	4	1
PRO	α4β3γ2[254]	3.25 (0.965-10.8)	63.0%	4	202	6	1
	α4β3δ[254]	9.26 (4.20-23.1)	116%	3	79.4	5	1
	α4β1γ2[239]	42.8% increase at 10μM PRO				1	1
PTB	α4β3γ2[254]	33.7 (9.38-119)	60.0%	4	195	6	1
	α4β3δ[254]	42.1 (9.45-196)	93.2%	4	590	6	1
	α4β2γ2[74,255]	70.8 (31.0-210)	339%	4	1518	6	2
	α4β1γ2[239]	141.2% increase at 100μM PTB				1	1

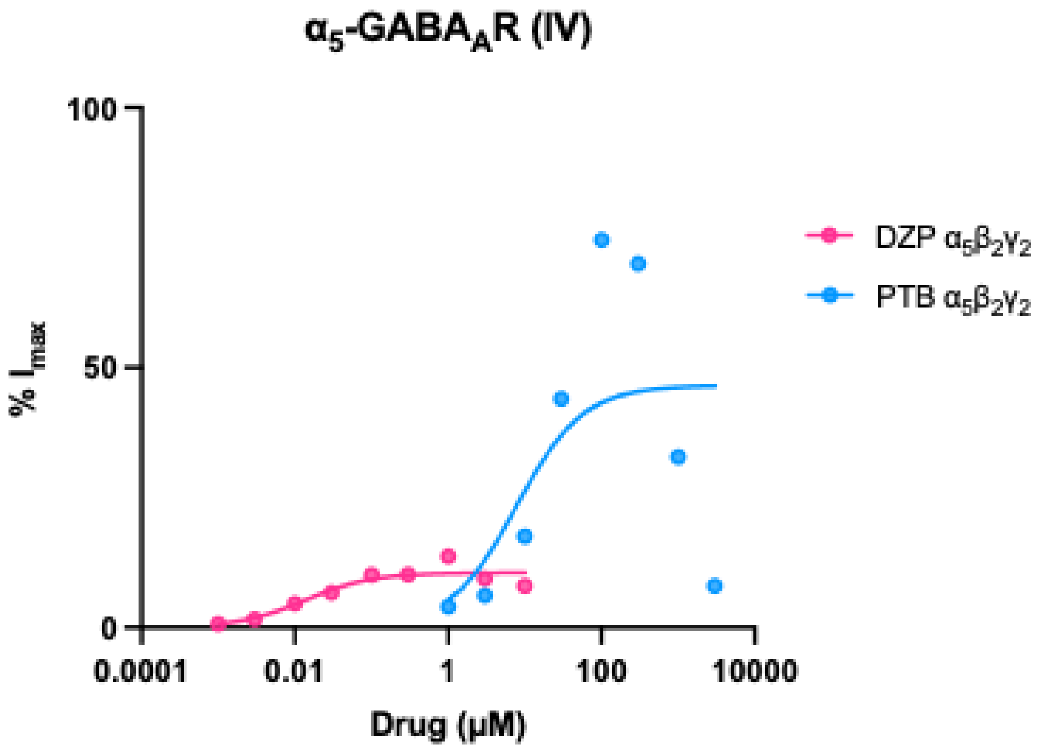

Figure S21

Drug	Subtype	EC_{50} µM (95% CI)	E_{max}	Goodness of Fit		n	
				df	ss	values	studies
DZP	α5β2γ2[141]	0.014 (0.004-0.040)	10.6%	7	19.6	9	1
PTB	α5β2γ2[73]	7.75	46.6%	6	3420	8	1

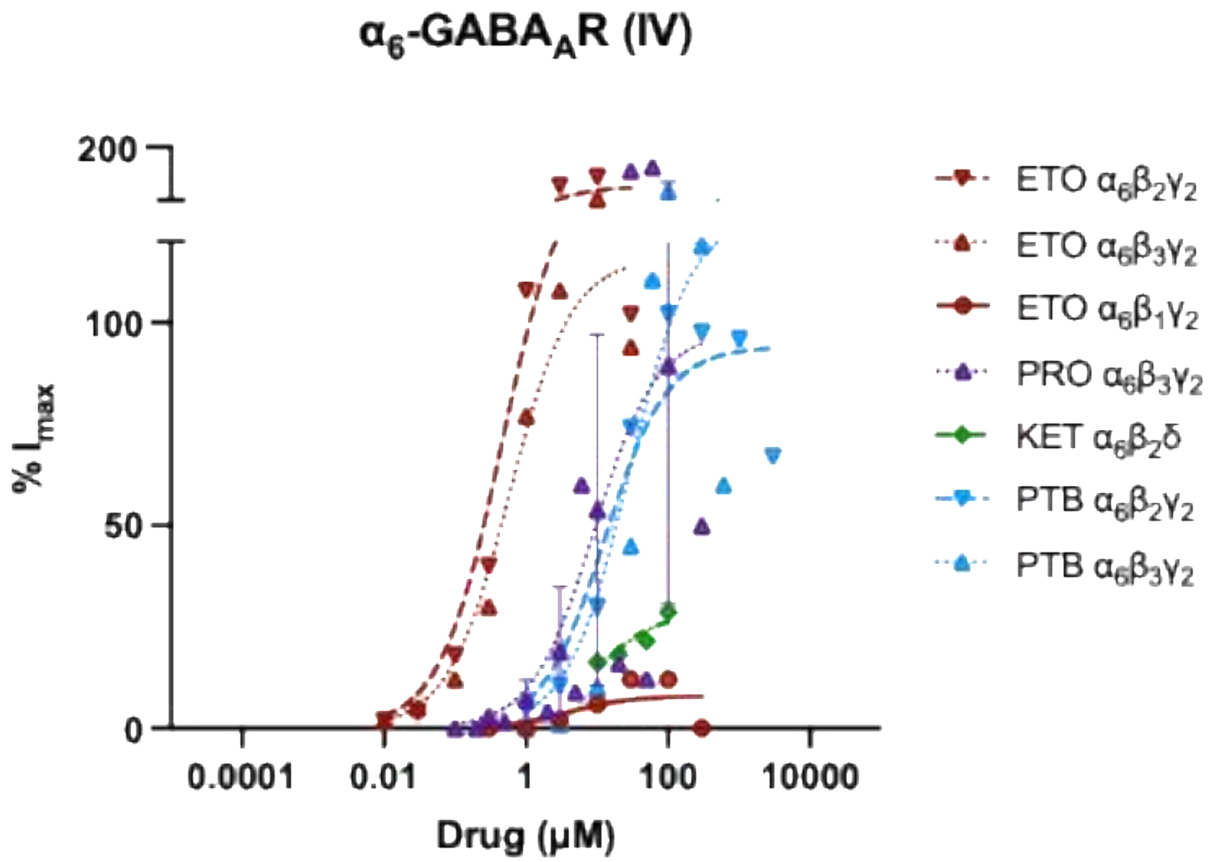

Figure S22

Drug	Subtype	EC_{50} μM (95% CI)	E_{max}	Goodness of Fit		n	
				df	ss	values	studies
ETO	α6β2γ2[30]	0.462 (0.150-1.26)	141%	6	2607	8	1
	α6β3γ2[256]	0.588 (0.272-1.21)	116%	5	791	8	1
	α6β1γ2[30]	4.02	8.0%	5	110	7	1
PRO	α6β3γ2[240,242,257]	9.09 (0.965-56.6)	97.6%	18	37355	20	3
	α6β1γ2[239]	24% increase at 10μM PRO				1	1
KET	α6β2δ[258]	9.67	28.8%	2	17.1	4	1
PTB	α6β2γ2[73]	13.0 (4.08-35.9)	94.1%	6	1377	8	1
	α6β3γ2[257,259]	28.8 (10.1-70.6)	127%	12	9362	14	2
DZP	α6β2γ2[147]	Not calculated – lack of effect				6	1

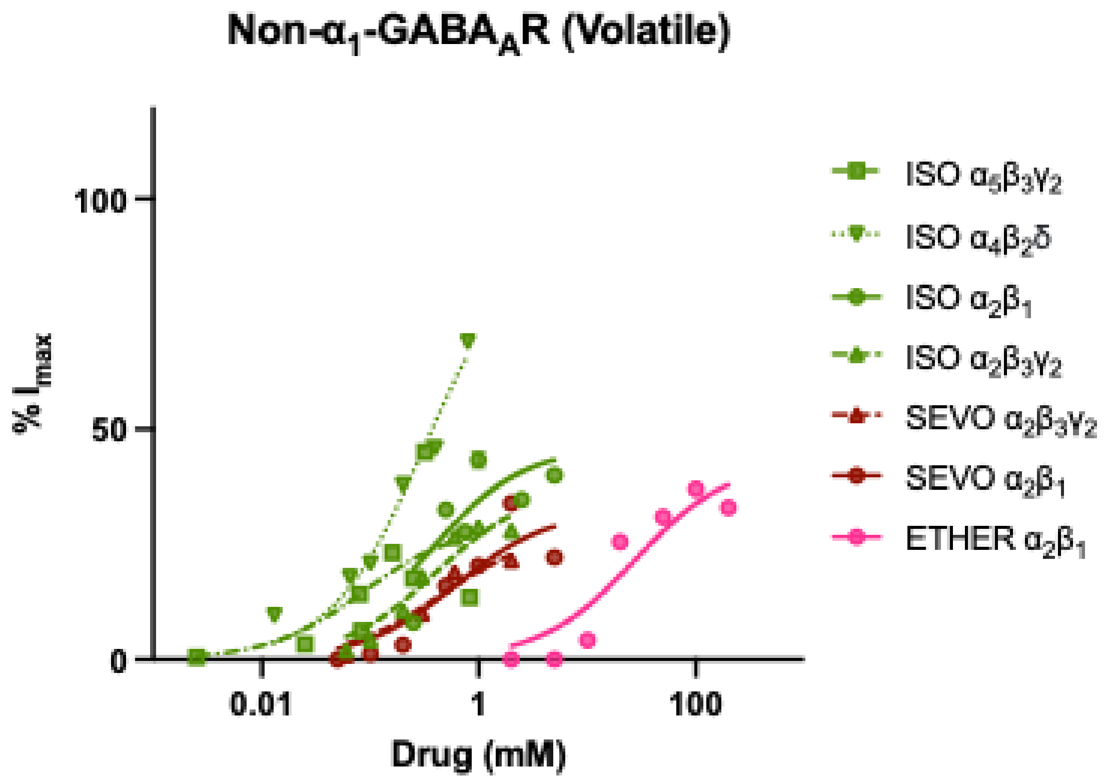

Figure S23

Drug	Subtype	EC_{50} mM (95% CI)	E_{max}	Goodness of Fit		n	
				df	ss	values	studies
ISO	α5β3γ2[249,260]	0.086	29.4%	6	763	8	2
	α4β2δ[197]	0.315 (0.131-0.884)	92.3%	4	85.9	6	1
	α2β1[206,261]	0.340 (0.006-1.31)	46.3%	5	382	7	2
	α2β3γ2[195]	0.397 (0.169-0.955)	37.6%	5	57.9	7	1
	α4β3[262]	15% increase at 0.06mM ISO; 71% at 0.29mM				2	1
	α4β3γ2[262]	9% increase at 0.06mM ISO; 32% at 0.29mM				2	1
	α4β3δ[262]	7% increase at 0.06mM ISO; 46% at 0.29mM				2	1
	α3β2γ2[263]	18.2% increase at 0.37mM ISO				1	1
SEVO	α2β3γ2[195]	0.435 (0.024-61.7)	27.7%	2	17.2	4	1
	α2β1[206]	0.624 (0.127-2.92)	32.6%	5	171	7	1
ETHER	α2β1[206]	27.3 (7.55-110)	43.1%	5	206	7	1
CHLOR	α2β1[264]	6% increase at 0.9mM CHLOR				1	1

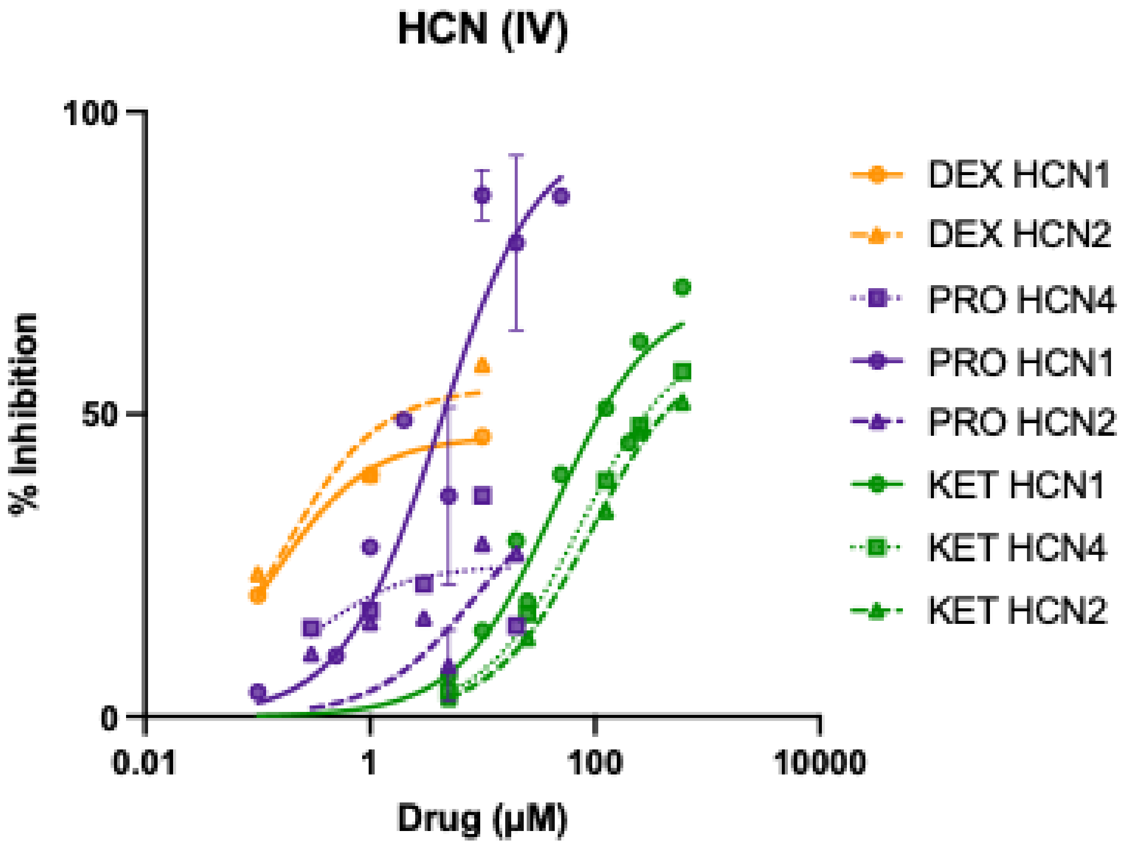

Figure S24

Drug	Subtype	IC_{50} μM (95% CI)	E_{max}	Goodness of Fit		n	
				df	ss	values	studies
DEX	HCN1[126]	0.135 (0.026-0.531)	47.5%	1	1.10	3	1
	HCN2[126]	0.167	54.5%	1	76.2	3	1
PRO	HCN4[26,265]	0.258	25.0%	3	249	5	2
	HCN1[25–28]	4.19 (0.748-8.98)	96.6%	11	4426	13	4
	HCN2[25,26,265,266]	8.16	38.5%	6	628	8	4
KET	HCN1[111,112]	44.4 (23.7-86.4)	69.7%	8	287	10	2
	HCN4[112]	74.2 (57.7-95.3)	63.2%	3	2.70	5	1
	HCN2[112]	92.0 (58.9-143)	61.2%	3	7.85	5	1
PTB	I_h[267]	Inhibition of I_h at 8μM PTB (value not specified)					1

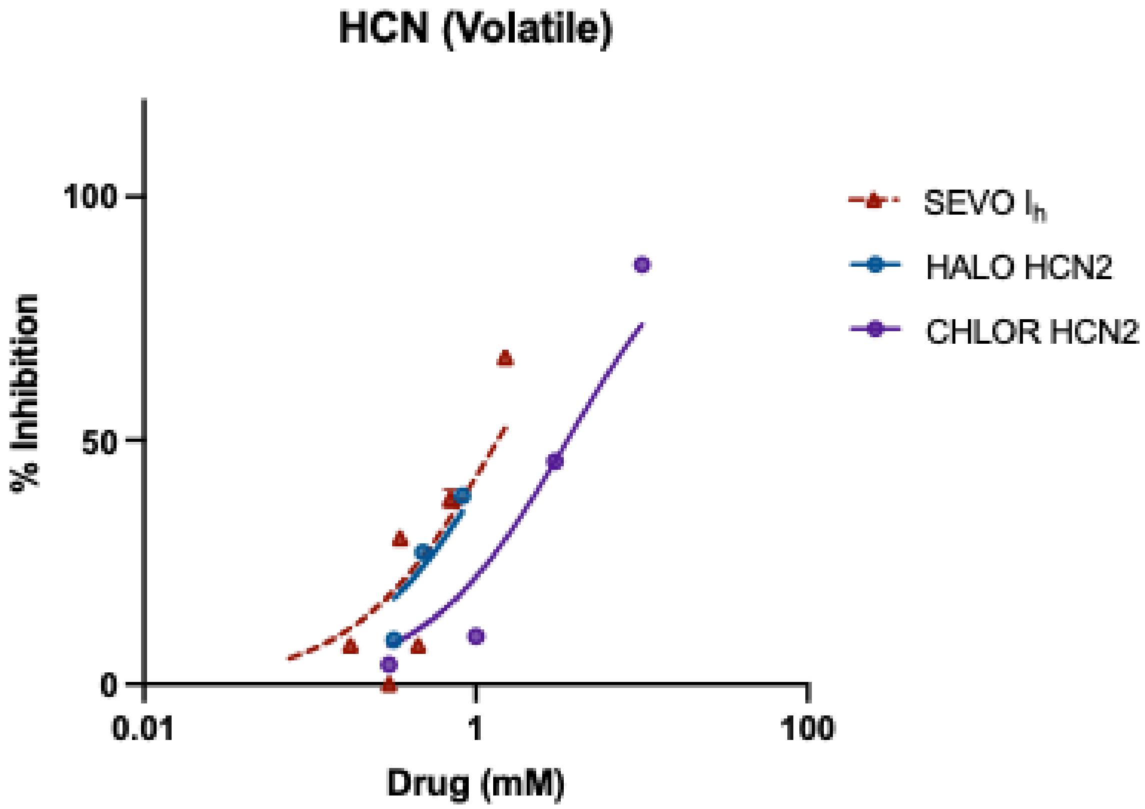

Figure S25

Drug	Subtype	IC_{50} mM (95% CI)	E_{max}	Goodness of Fit		n	
				df	ss	values	studies
SEVO	I_h[207,208]	1.35 (0.276-2.79)	100%	6	1143	8	2
HALO	HCN2[171]	1.51	100%	1	90.1	3	1
	HCN1[171]	7% inhibition at 0.83mM HALO				3	1
CHLOR	HCN2[220]	3.56 (0.167-16.1)	100%	2	312	4	1
ISO	I_h[203]	45% inhibition at 0.9mM ISO				1	1
XE	I_h[236]	33.4% inhibition at 1.9mM XE				1	1
	HCN2[236]	16.1% inhibition at 1.9mM XE				1	1

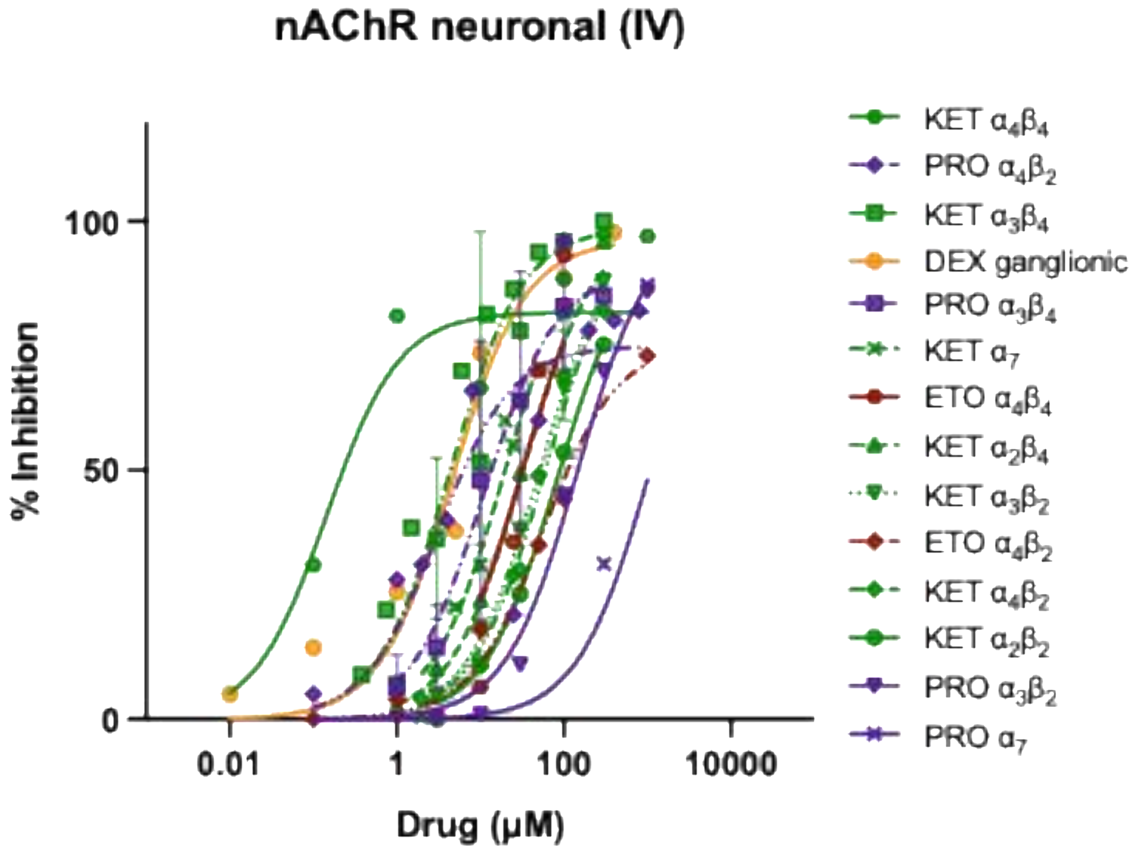

Figure S26A

Table over page

Drug	Subtype	IC_{50} μM (95% CI)	E_{max}	Goodness of Fit		n	
				df	ss	values	studies
KET	α4β4[105,101]	0.144	81.7%	6	3302	8	2
	α3β4[105,268]	4.28 (2.34-6.67)	98.9%	12	1547	14	2
	α7[269,270]	19.8 (11.9-24.8)	100%	8	276	10	2
	α2β4[105]	32.8 (18.1-40.3)	100%	4	84.9	6	1
	α3β2[105]	51.1 (36.8-57.3)	100%	3	11.4	5	1
	α4β2[105,269]	60.2 (34.5-74.6)	100%	7	300	9	2
	α2β2[105]	83.0 (56.0-108)	96.5%	3	12.9	5	1
PRO	α4β2[23,24]	3.13 (0.387-21.7)	75.0%	9	3097	11	2
	α3β4[271]	11.0 (2.62-34.3)	90.4%	9	3605	11	1
	α3β2[272]	147 (77.2-212)	100%	5	222	8	1
	α7[272]	1081	100%	6	15878	8	1
DEX	ganglionic[128]	4.62 (1.52-10.3)	96.3%	5	476	7	1
ETO	α4β4[101]	31.8	100%	2	757	4	1
	α4β2[273]	56.5 (26.4-105)	75.4%	4	69.5	6	1
PTB	α4β2[185]	78% inhibition at 50μM PTB; 89% at 100μM				2	1
	α4β4[185]	37% inhibition at 50μM PTB; 53% at 100μM				2	1

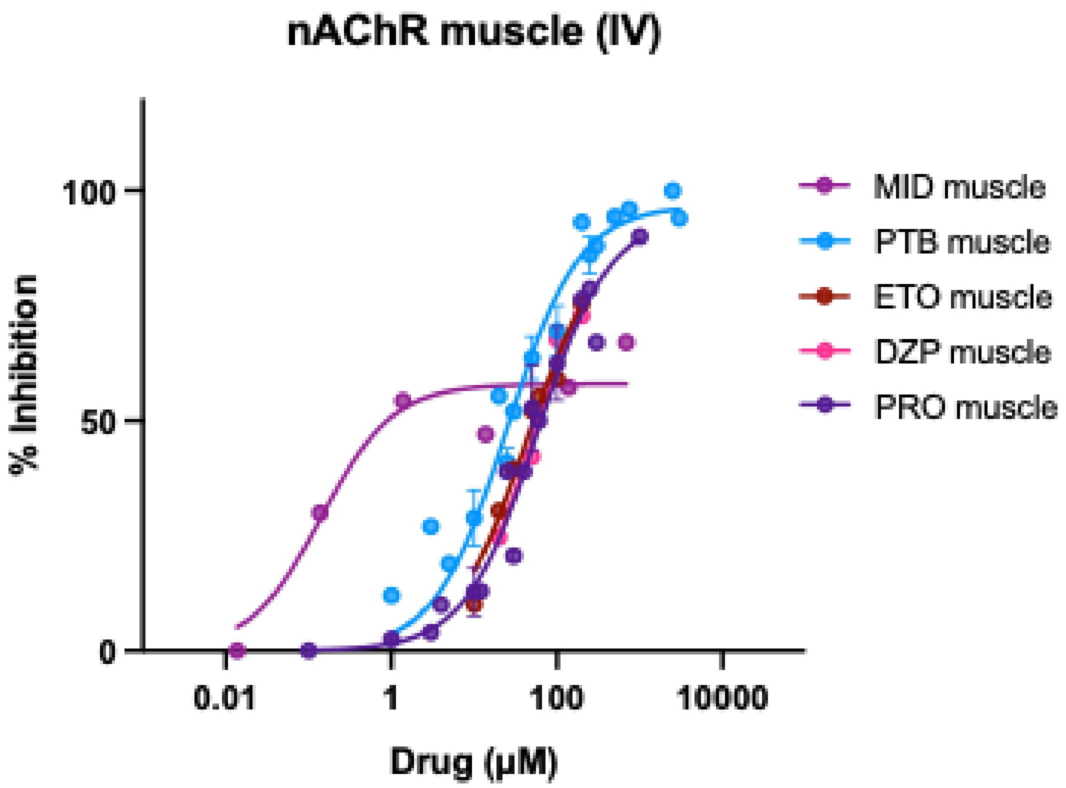

Figure S26B

Drug	Subtype	IC_{50} μM (95% CI)	E_{max}	Goodness of Fit		n	
				df	ss	values	studies
MID	muscle[138]	0.143 (0.034-0.580)	58.0%	4	219	6	1
PTB	muscle[69–72]	25.1 (18.7-33.1)	96.8%	23	1325	25	4
ETO	muscle[148]	42.0 (25.1-63.0)	90.4%	5	92.5	7	1
DZP	muscle[148]	57.0 (13.0-103)	97.1%	2	52.9	4	1
PRO	muscle[24,148,209,272]	57.8 (37.6-80.4)	94.6%	24	2185	26	4

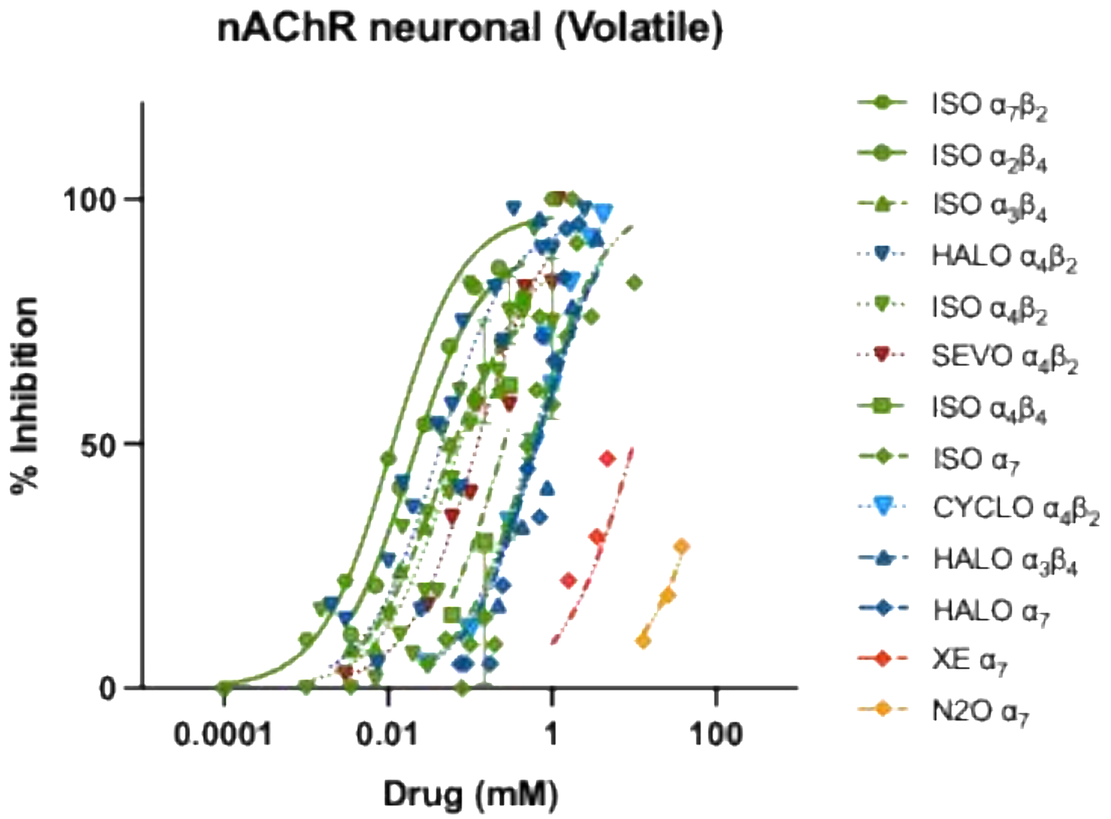

Figure S27A

Table over page

Drug	Subtype	IC_{50} mM (95% CI)	E_{max}	Goodness of Fit		n	
				df	ss	values	studies
ISO	α7β2[274]	0.011 (0.008-0.015)	97.3%	4	41.8	6	1
	α2β4[183]	0.019 (0.013-0.026)	90.4%	6	107	8	1
	α3β4[183]	0.039 (0.024-0.064)	80.9%	6	127	8	1
	α4β2[23,24,183–185,156]	0.060 (0.038-0.094)	89.2%	27	3458	29	6
	α4β4[184,185]	0.260	100%	1	129	3	2
	α7[275,276]	0.552 (0.362-0.722)	100%	19	2543	21	2
HALO	α4β2[24,155,156]	0.041 (0.023-0.066)	96.1%	15	2000	17	3
	α3β4[277]	0.630	100%	4	2383	6	1
	α7[155,277]	0.674 (0.304-1.19)	100%	10	4068	12	2
SEVO	α4β2[24,156]	0.123 (0.074-0.164)	100%	8	425	10	2
CYCLO	α4β2[223,169]	0.622 (0.262-0.741)	100%	7	501	9	2
XE	α7[229]	9.86	100%	1	304	3	1
	α4β2[184,185]	39% inhibition at 2mM XE				2	2
	α4β4[184,185]	12% inhibition at 2mM Xe				2	2
N2O	α7[229]	102.6	100%	1	7.53	3	1
	α2β2[185]	40% inhibition at 12.2mM N2O				1	1
	α2β2[185]	10% inhibition at 12.2mM N2O				1	1
	α3β2[185]	33% inhibition at 12.2mM N2O				1	1
	α3β4[185]	4% inhibition at 12.2mM N2O				1	1
	α4β2[184,185]	41% inhibition at 12.2mM N2O				2	2
	α4β4[184,185]	7% inhibition at 12.2mM N2O				2	2

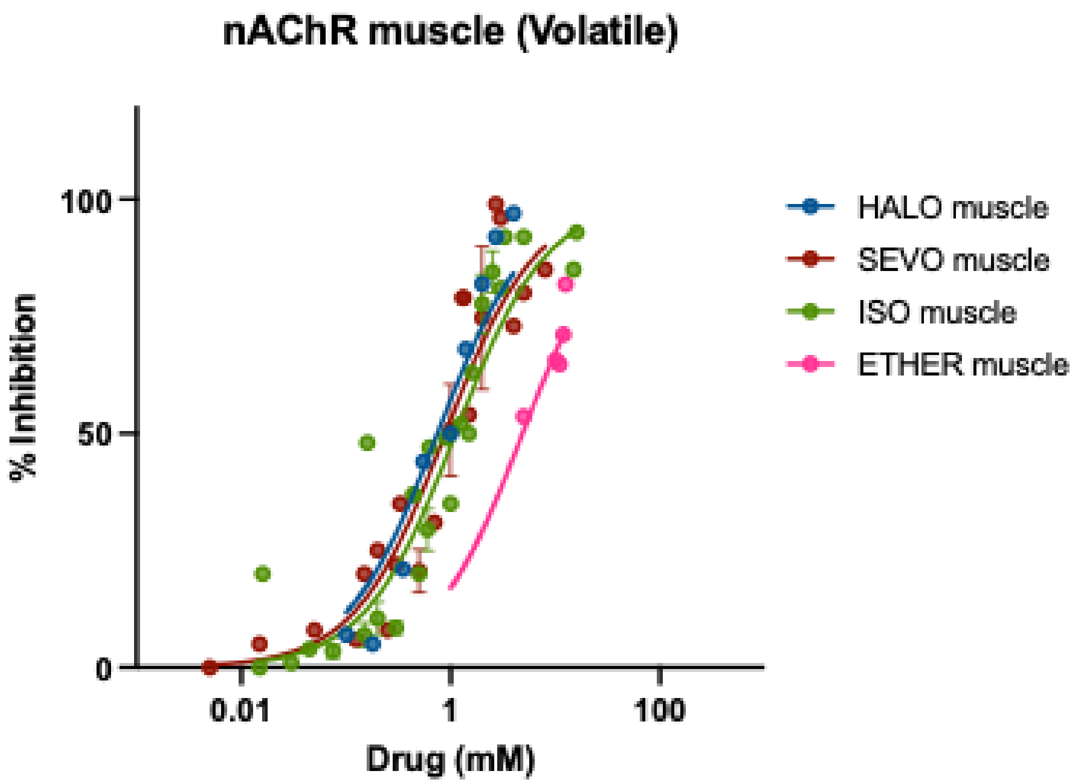

Figure S27B

Drug	Subtype	IC_{50} mM (95% CI)	E_{max}	Goodness of Fit		n	
				df	ss	values	studies
HALO	muscle[24]	0.748 (0.461-1.13)	100%	7	846	9	1
SEVO	muscle[24,278–280]	0.890 (0.573-1.19)	100%	26	5960	28	4
ISO	muscle[281,278,24,282,279,280]	1.11 (0.693-1.26)	100%	33	4689	35	6
ETHER	muscle[209]	4.87	100%	3	132	5	1

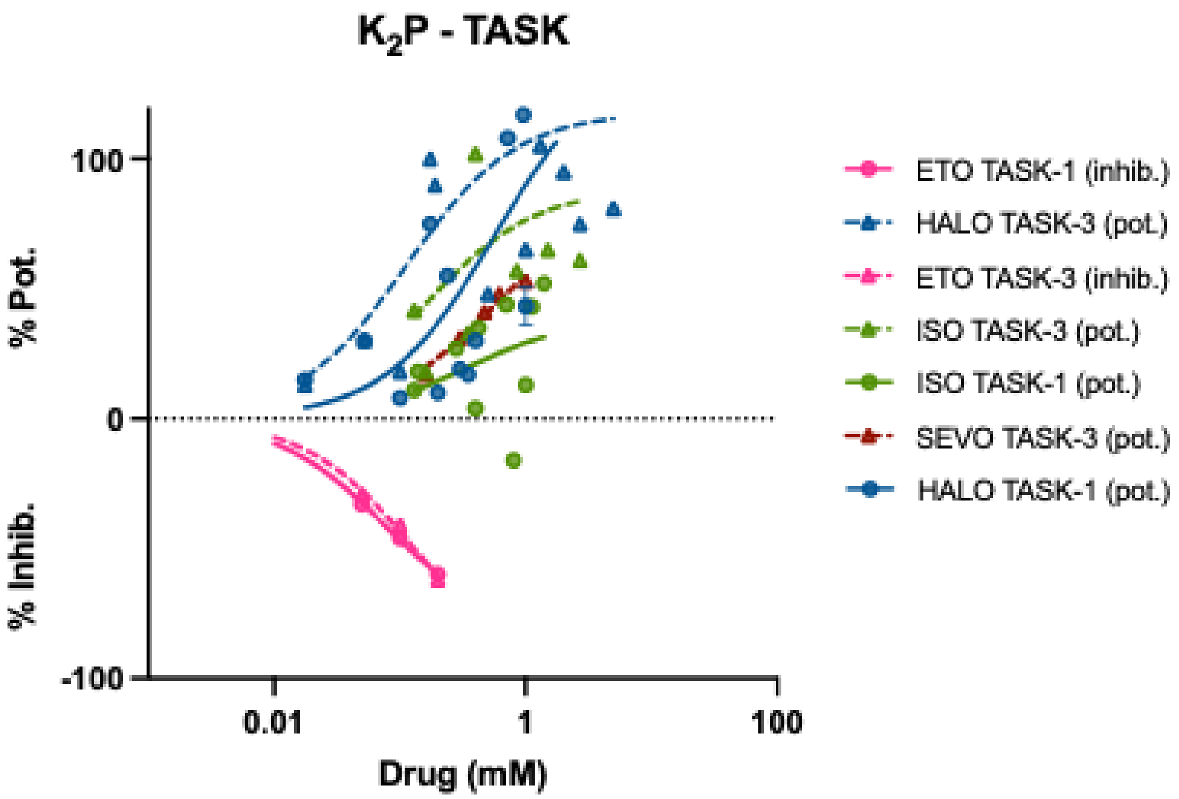

Figure S28A

7 dps outside axis limits

Drug	Subtype	EC_{50}/IC_{50} mM (95% CI)	E_{max}	Goodness of Fit		n	
				df	ss	values	studies
Potentiation							
HALO	TASK-3[157–159]	0.113 (0.003-0.834)	118%	11	27022	13	3
	TASK-1 [102,158,214,283,284]	0.563	141%	16	21564	18	5
ISO	TASK-3[159,285]	0.162	88.8%	6	8132	8	2
	TASK-1[102,284,285]	0.340	39.2%	8	3656	10	3
SEVO	TASK-3[159]	0.512 (0.251-1.11)	83.2%	3	19.4	5	1
	TASK-1[102]	45% potentiation at 1mM SEVO				1	1
Inhibition							
ETO	TASK-1[102]	0.077 (0.020-0.154)	82.8%	1	0.80	3	1
	TASK-3[102]	0.130	100%	1	9.57	3	1

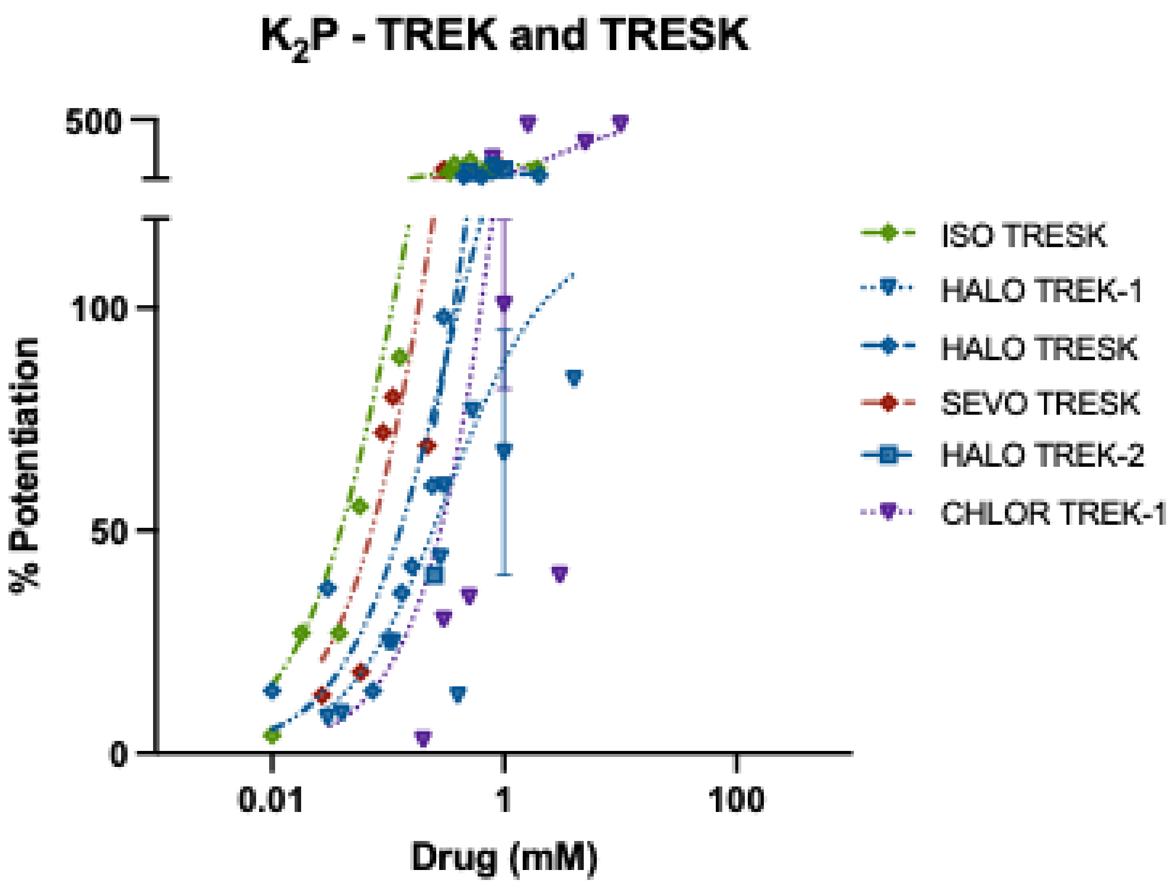

Figure 28B

Table over page

Drug	Subtype	EC_{50} mM (95% CI)	E_{max}	Goodness of Fit		n	
				df	ss	values	studies
ISO	TRESK[205]	0.139 (0.060-0.315)	228%	9	7945	11	1
	TREK-1[214]	30% potentiation at 0.3mM ISO; 60% at 1mM ISO				2	1
	TREK-2[286]	90% potentiation at 1mM ISO				1	1
HALO	TREK-1[214,224]	0.322 (0.010-2.41)	116%	10	19517	12	2
	TRESK[205]	0.338 (0.141-0.740)	183%	8	3261	10	1
	TREK-2[286]	1.11	402%	1	2495	3	1
SEVO	TRESK[205]	0.348 (0.085-2.16)	290%	5	4730	7	1
CHLOR	TREK-1 [104,214,218,219]	2.74	536%	9	161090	11	4
	TREK-2[286]	80% potentiation at 1mM CHLOR				1	1
	TRESK[287]	31.5% potentiation at 5mM CHLOR				1	1
ETO	TREK-1[104]	60.2% potentiation at 10μM ETO				1	1
ETHER	TREK-1[214]	80% potentiation at 0.6mM ETHER				1	1
CYCLO	TREK-1^[224]	0% potentiation at 0.09mM CYCLO; 14% at 0.46mM; 26% at 0.91mM; 113% at 3.65mM; 189% at 5.48mM; 277% at 7.3mM				6	1
XE	TREK-1^[224,234,235]	6% potentiation at 0.22mM XE; 10% at 0.86mM; 14% at 1.72mM; 26% at 2.58mM; 35.7% at 3.44mM				7	3
N2O	TREK-1^[224]	3% potentiation at 1.46mM N2O; 6% at 5.84mM; 10% at 11.7mM; 13% at 17.5mM; 26% at 23.4mM				5	1

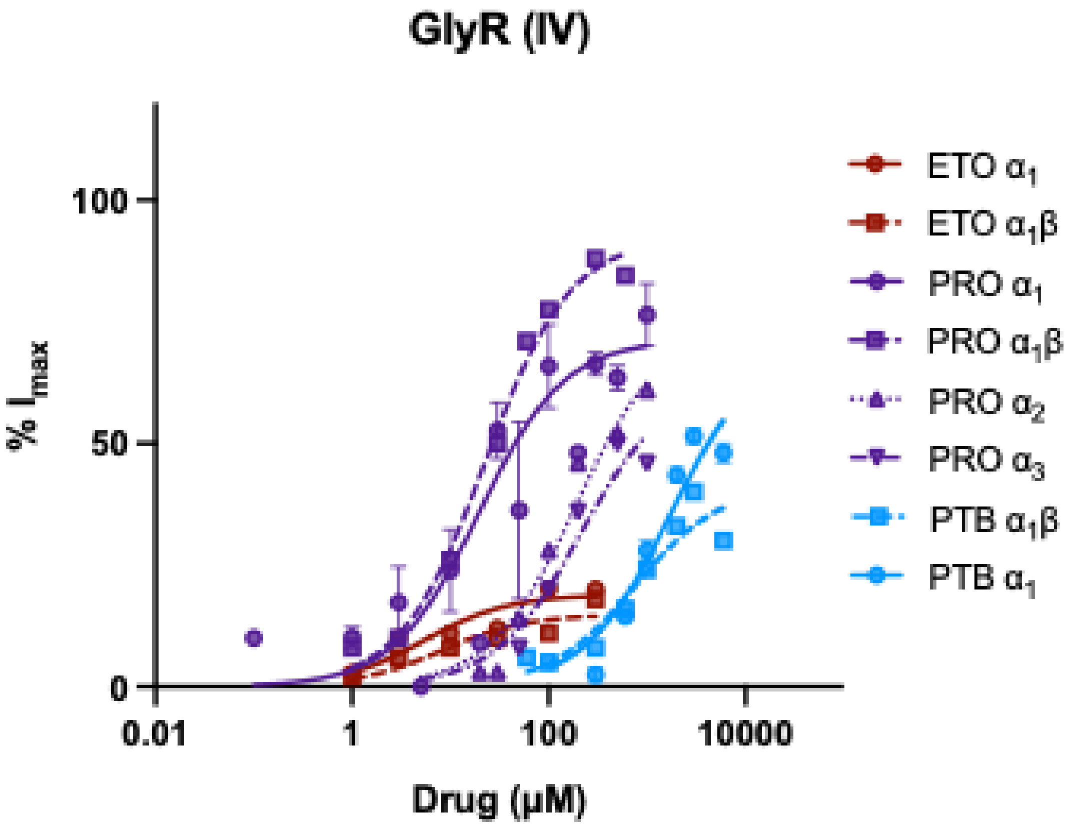

Figure S29

Drug	Subtype	EC_{50} μM (95% CI)	E_{max}	Goodness of Fit		n	
				df	ss	values	studies
ETO	α1[58]	5.37 (0.791-33.0)	19.0%	4	34.7	6	1
	α1β[58]	8.24 (0.79-87.2)	14.9%	4	27.0	6	1
PRO	α1[58,243,244,259,288,289]	19.5 (9.21-38.4)	71.4%	31	7294	33	6
	α1β[58]	22.6 (16.5-30.5)	92.4%	6	74.7	8	1
	α2[243,290]	187 (104-352)	74.1%	7	180	9	2
	α3[243]	209 (80.0-618)	62.6	6	258	8	1
	α2β[290]	21% increase at 30μM PRO				1	1
PTB	α1β[58]	756 (273-1922)	41.4%	6	131	8	1
	α1[58,259]	1704 (912-3315)	70.3%	12	579	14	2

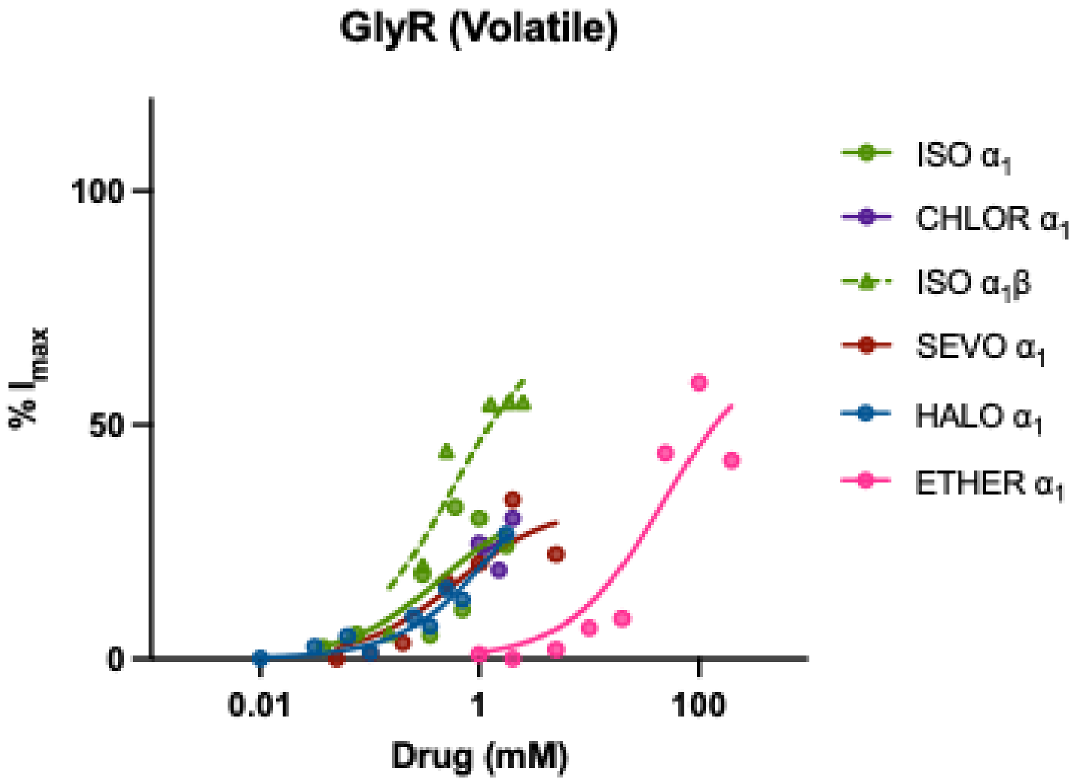

Figure S30

Drug	Subtype	EC_{50} mM (95% CI)	E_{max}	Goodness of Fit		n	
				df	ss	values	studies
ISO	α1[169,170,206,291,292]	0.445	34.3%	6	442	8	5
	α1β[199]	0.571 (0.154-2.57)	72.9%	4	268	6	1
CHLOR	α1[215–217]	0.480	32.9%	1	53.2	3	3
SEVO	α1[206]	0.615 (0.128-2.81)	32.7%	5	168	7	1
	α1β[199]	6.3% increase at 0.3mM SEVO				1	1
HALO	α1[168–170]	1.50 (0.529-8.56)	48.6%	7	39.6	9	3
	α1β[199]	22.3% increase at 0.18mM HALO				1	1
ETHER	α1[206]	47.3 (11.6-250)	66.9%	6	594	8	1
N2O	α1^[168,184]	1.7% increase at 12.2mM N2O; 3.8% at 22.5mM; 5.7% at 113mM; 25% at 225mM; 69.6% at 338mM				5	2
XE	α1^[168,184]	Not calculated – lack of effect				7	2

NB: HALO and ISO fits include EC_5-EC_{10} data; N2O and Xe fits include EC_5 data

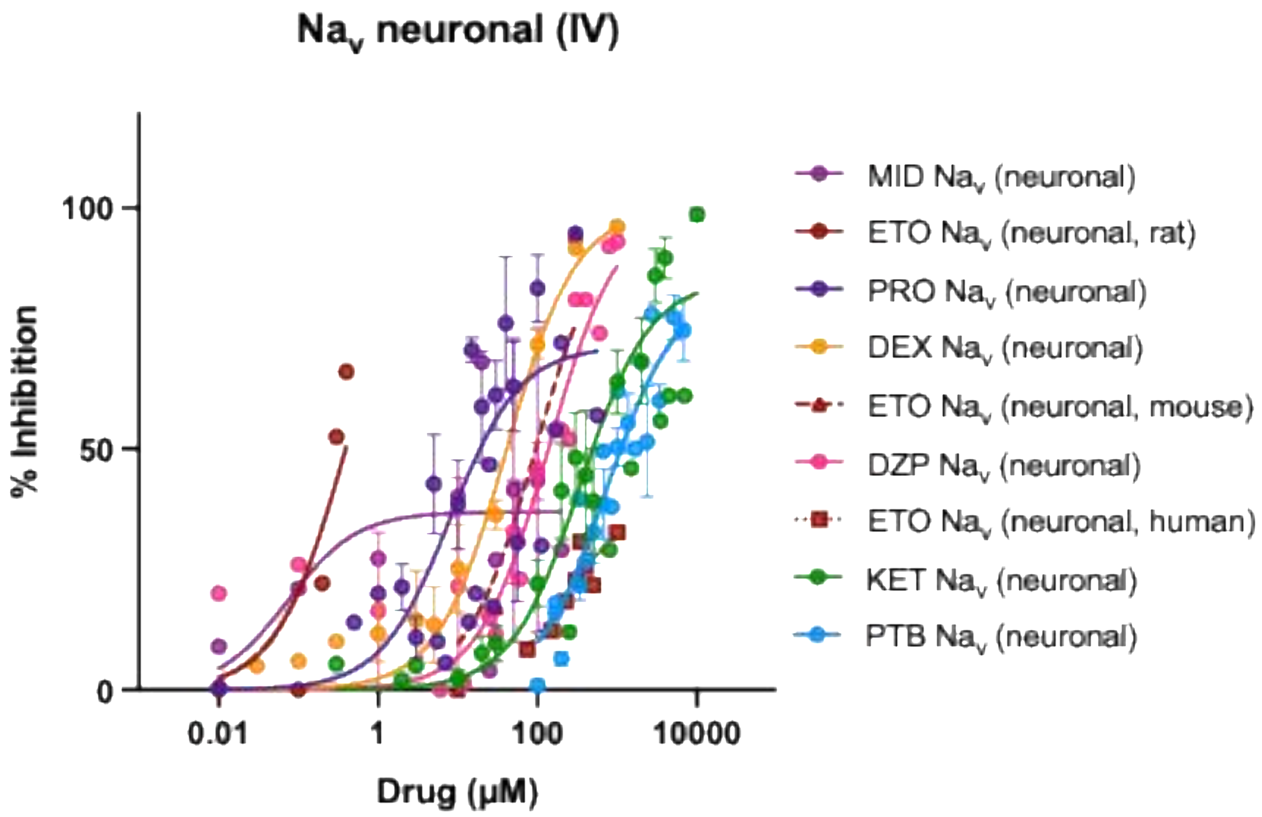

Figure S31A

Drug	Subtype	IC_{50} µM (95% CI)	E_{max}	Goodness of Fit		n	
				df	ss	values	studies
MID	neuronal[135–137]	0.074	37.0%	10	6392	12	3
ETO	neuronal, rat[103]	0.393	100%	3	880	5	1
	neuronal, mouse[46]	95.1	100%	2	505	4	1
	neuronal, human[293]	264 (82.3-754)	41.3%	6	90.3	8	1
PRO	neuronal[44–53]	8.08 (3.23-16.9)	71.3%	50	23009	52	10
DEX	neuronal[129,127,130,131,128,132]	41.3 (25.7-53.9)	100%	25	2580	27	6
DZP	neuronal[152–154,136]	137 (72.1-181)	100%	29	5936	31	4
KET	neuronal[44,115,46,116–121]	348 (206-571)	85.2%	71	22831	73	9
PTB	neuronal[83,44,84–90]	739 (529-1020)	83.3%	49	5309	51	9

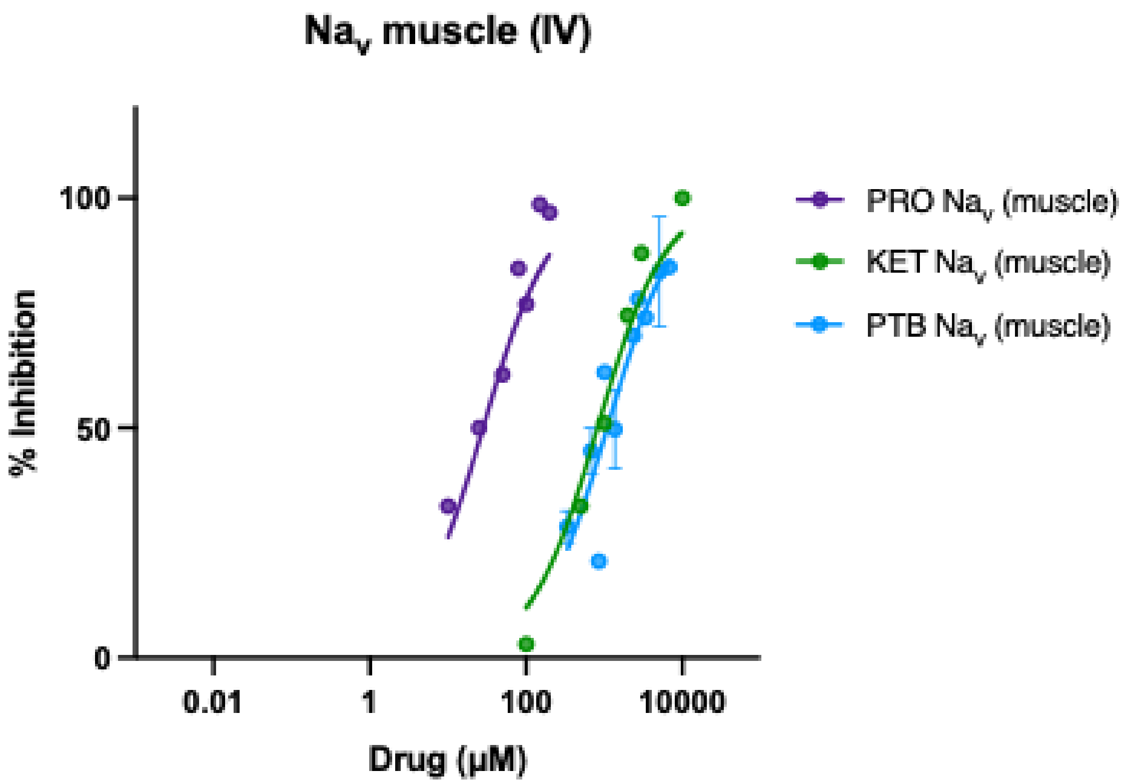

Figure 31B

Drug	Subtype	IC_{50} μM (95% CI)	E_{max}	Goodness of Fit		n	
				df	ss	values	studies
PRO	muscle[294]	28.2 (8.29-37.8)	100%	5	468	7	1
KET	muscle[295]	821 (458-1303)	100%	4	261	6	1
PTB	muscle[86,88]	1095	100%	13	1797	15	2

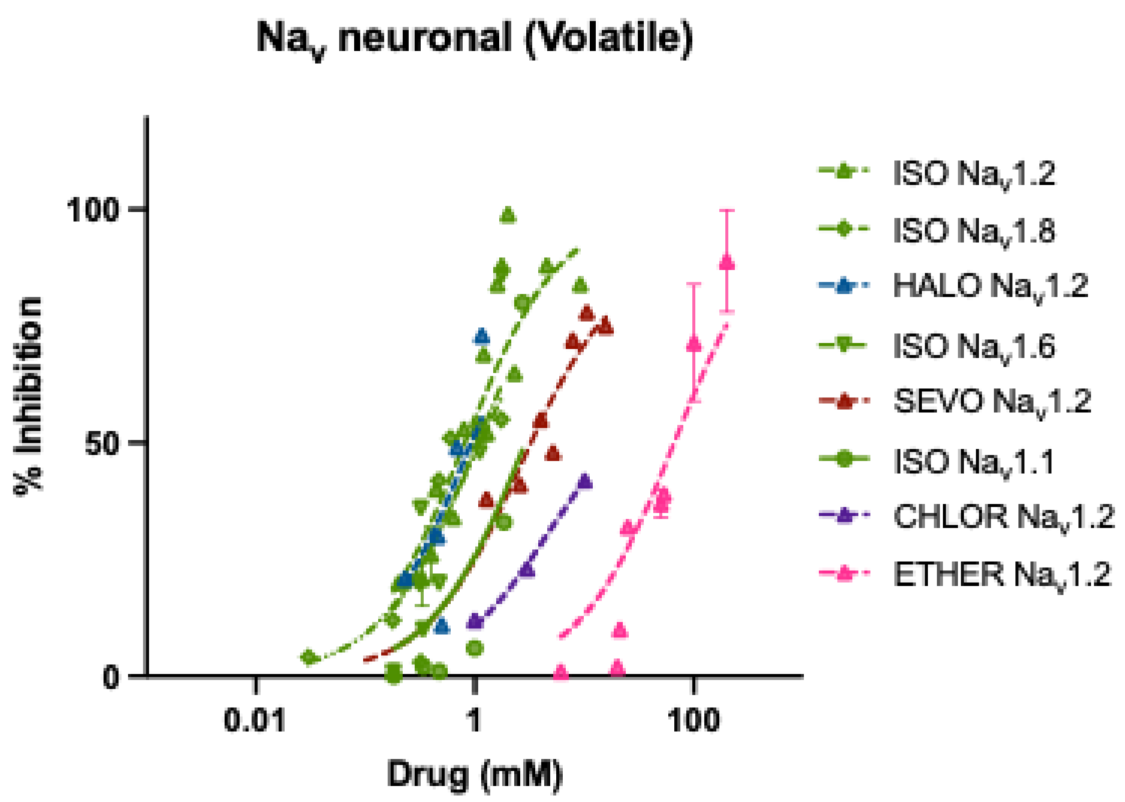

Figure S32A

Drug	Subtype	IC_{50} mM (95% CI)	E_{max}	Goodness of Fit		n	
				df	ss	values	studies
ISO	Nav1.2[165,200–202]	0.810 (0.542-1.07)	100%	17	2646	19	4
	Nav1.8[165,296]	0.893 (0.074-2.32)	90.0%	5	811	7	2
	Nav1.6[165,202]	1.05 (0.102-2.99)	100%	4	1229	6	2
	Nav1.1[202]	2.89 (0.012-17.3)	100%	4	1693	6	1
HALO	Nav1.2[50,165]	0.930	100%	3	940	5	2
SEVO	Nav1.2[200]	2.57 (0.782-4.91)	89.5%	5	289	7	1
	Nav1.7[297]	8% inhibition at 0.5mM SEVO; 15.8% at 1mM				2	1
	Nav1.8[297]	11% inhibition at 0.5mM SEVO; 16.8% at 1mM				2	1
CHLOR	Nav1.2[222]	4.68 (0.116-24.8)	61.3%	1	2.41	3	1
ETHER	Nav1.2[212,44,200]	65.9 (35.2-105)	100%	9	2045	11	3
CYCLO	Nav1.2[165]	20% inhibition at 3.29 mM CYCLO				1	1

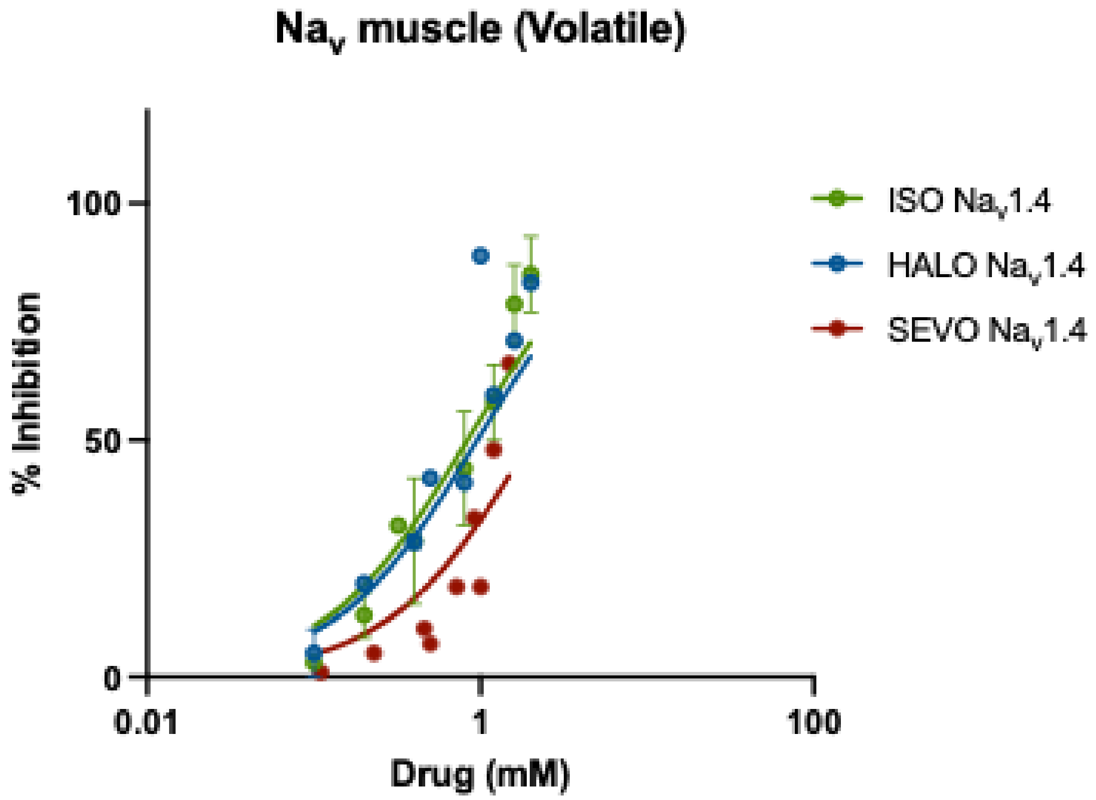

Figure S32B

Drug	Subtype	IC_{50} mM (95% CI)	E_{max}	Goodness of Fit		n	
				df	ss	values	studies
ISO	Nav1.4[165,201,298]	0.835 (0.448-1.10)	100%	12	2073	14	3
HALO	Nav1.4[298,299]	0.957 (0.226-1.24)	100%	9	1942	11	2
SEVO	Nav1.4[297,298]	2.01 (0.376-3.70)	100%	7	1215	9	2

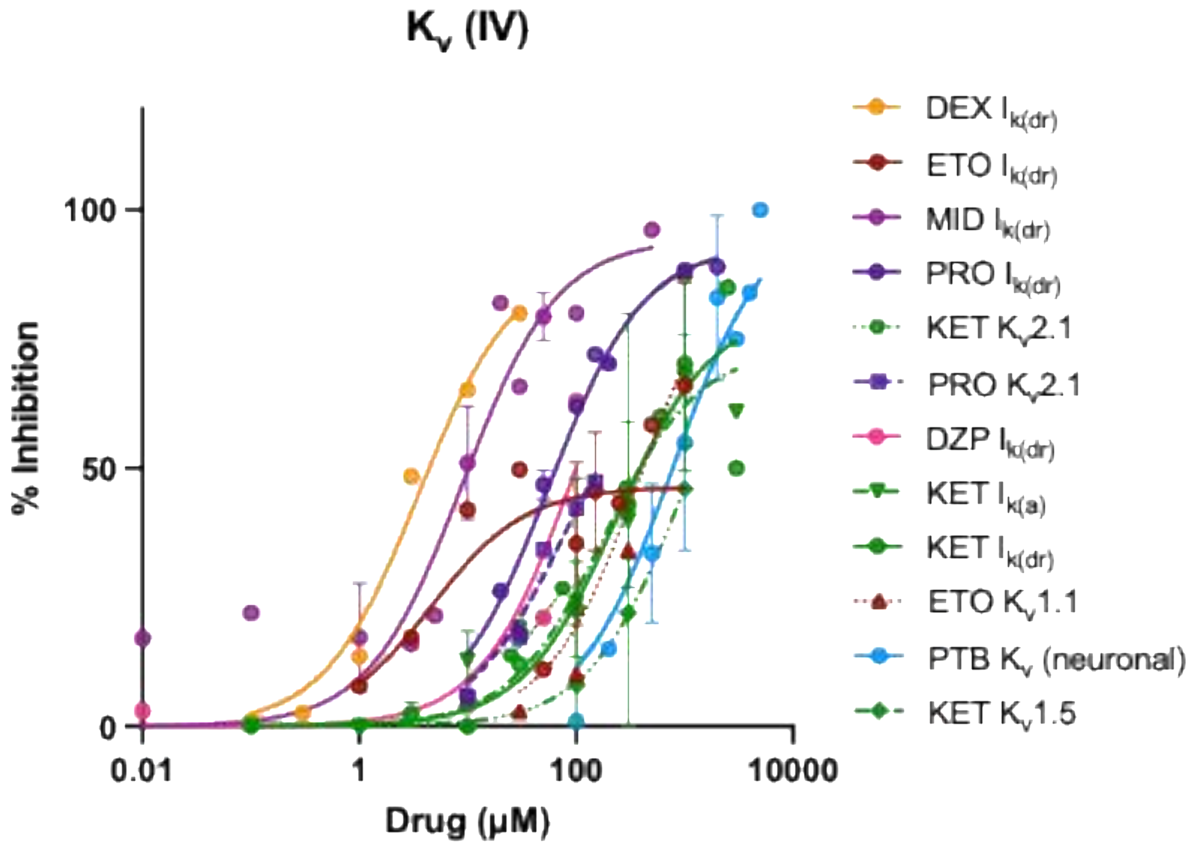

Figure S33

Drug	Subtype	IC_{50} μM (95% CI)	E_{max}	Goodness of Fit		n	
				df	ss	values	studies
DEX	$I_{k(dr)}$ [127]	3.54 (1.79-6.66)	90.0%	4	114	6	1
ETO	$I_{k(dr)}$ [62,98,99]	4.59 (0.150-152)	46.3%	10	2664	12	3
	K_v1.1[300]	416	100%	2	466	4	1
MID	$I_{k(dr)}$ [63,136,137]	9.03 (4.00-16.7)	94.4%	12	1952	14	3
PRO	$I_{k(dr)}$ [62–65]	56.5 (43.0-73.3)	93.2%	13	448	15	4
	K_v2.1[301]	76.1 (23.3-182)	73.5%	3	50.4	5	1
KET	K_v2.1[114]	71.7 (9.79-239)	52.1%	1	0.24	3	1
	$I_{k(a)}$ [118,302,303]	181 (5.57-1203)	73.3%	11	6874	13	3
	$I_{k(dr)}$ [62,63,118,302–304]	236 (64.8-647)	80.5%	20	8769	22	6
	K_v1.5[305]	947 (686-1212)	89.7%	3	1.39	5	1
DZP	$I_{k(dr)}$ [136]	99.8 (9.83-243)	100%	4	418	6	1
PTB	K_v (neuron)[44,63]	776 (338-1330)	100%	9	2406	11	2

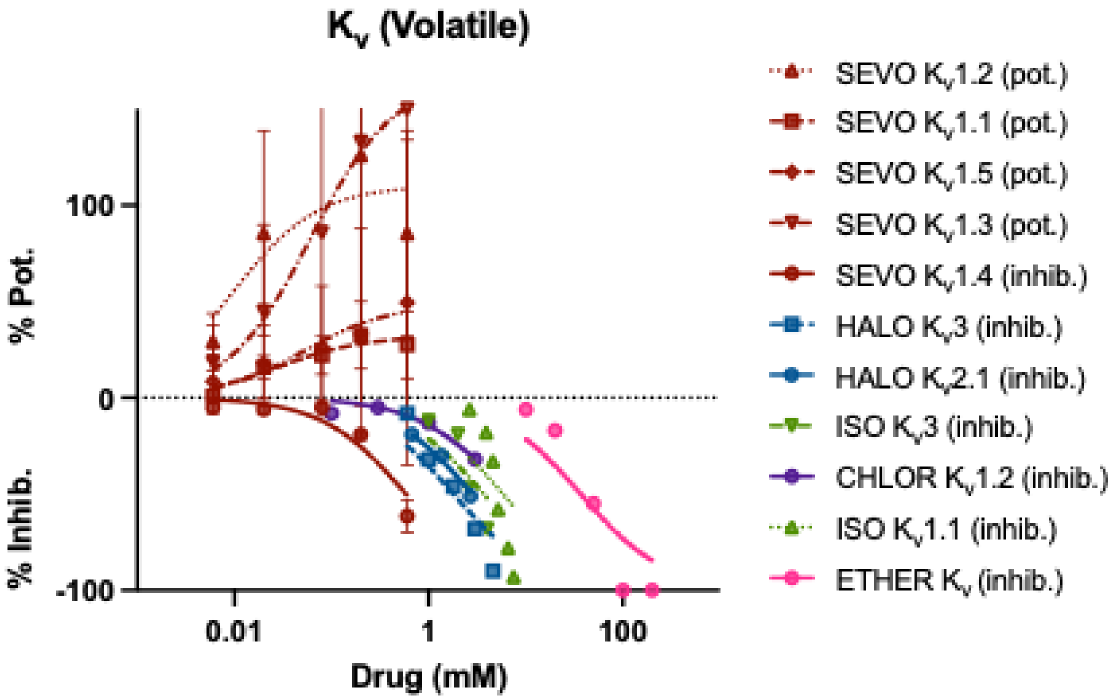

Figure S34

Drug	Subtype	EC_{50}/IC_{50} mM (95% CI)	E_{max}	Goodness of Fit		n	
				df	ss	values	studies
Potentiation							
SEVO	Kv1.2[204]	0.0097	110%	6	32096	8	1
	Kv1.1[204]	0.028	32.0%	8	1620	10	1
	Kv1.5[204]	0.052	48.5%	8	23569	10	1
	Kv1.3[204]	0.062	167%	8	109620	10	1
ISO	Kv1.3[204]	-17% to 42% potentiation between 0.006 to 0.9 mM ISO				12	1
Inhibition							
SEVO	Kv1.4[204]	0.586 (0.245-0.855)	100%	8	670	10	1
HALO	Kv3[177]	1.81 (0.193-4.62)	100%	3	678	5	1
	Kv2.1[114]	2.83	100%	1	10.0	3	1
ISO	Kv3[177]	3.70	100%	2	635	4	1
	Kv1.1[177]	5.84	100%	4	3337	6	1
CHLOR	Kv1.2[221]	4.63 (0.006-17.5)	80.8%	2	41.5	4	1
ETHER	K_v (neuron)[44]	36.9 (1.90-159)	100%	3	1540	5	1

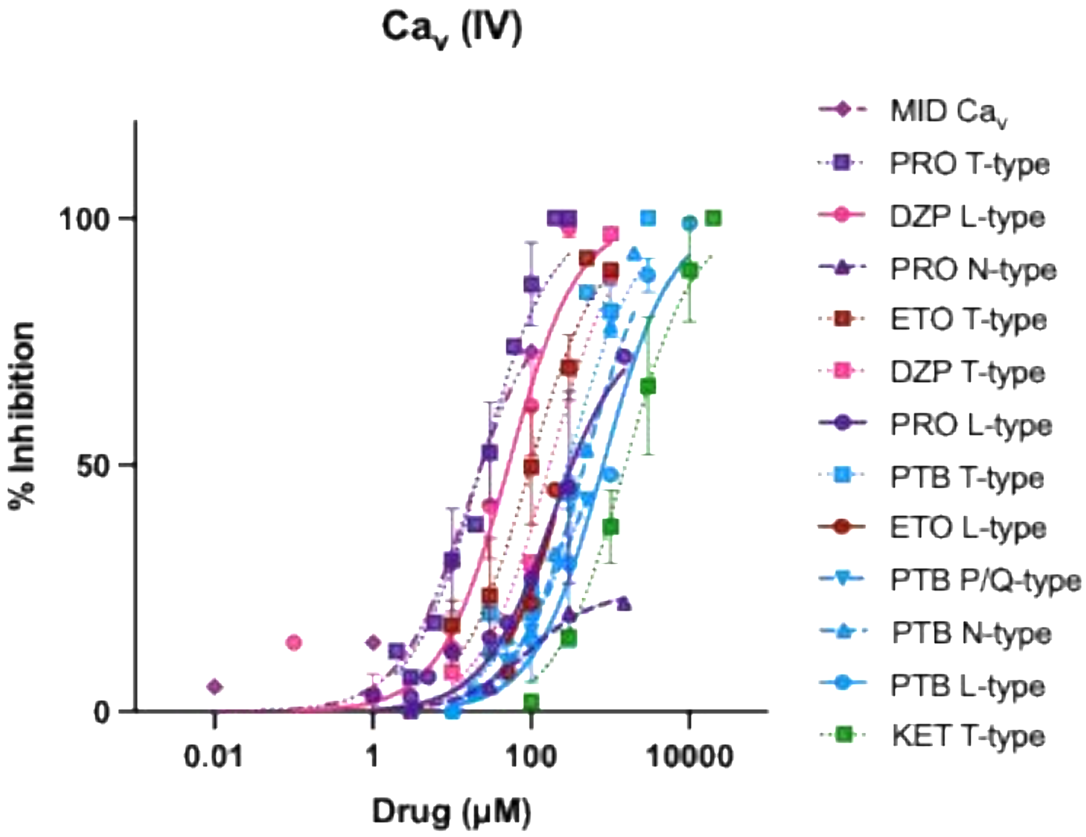

Figure S35

Drug	Subtype	IC_{50} μM (95% CI)	E_{max}	Goodness of Fit		n	
				df	ss	values	studies
MID	Ca_V[136]	16.0 (0.594-99.4)	84.2%	2	112	4	1
PRO	T-type[59–61]	23.7 (15.3-33.7)	100%	17	3243	19	3
	N-type[306]	91.6 (26.3-326)	24.3%	2	4.31	4	1
	L-type[306,307]	181 (39.7-568)	77.6%	8	73.4	10	2
	P-type[308]	2.8% inhibition at 2μM PRO				1	1
DZP	L-type[149–151]	52.0 (27.8-78.0)	100%	15	2418	17	3
	T-type[149]	171	100%	1	182	3	1
ETO	T-type[59–61]	93.9 (30.9-172)	97.2%	13	3909	15	3
	L-type[81]	304	100%	1	73.4	3	1
PTB	T-type[59–61,82]	291 (218-372)	100%	21	2654	23	4
	P/Q-type[308,309]	312 (110-444)	69.8%	3	30.5	5	2
	N-type[310]	471 (176-596)	100%	5	293	7	1
	L-type[151]	812 (345-1182)	100%	9	1192	11	1
KET	T-type[59,60]	1551 (815-2498)	100%	7	818	9	2

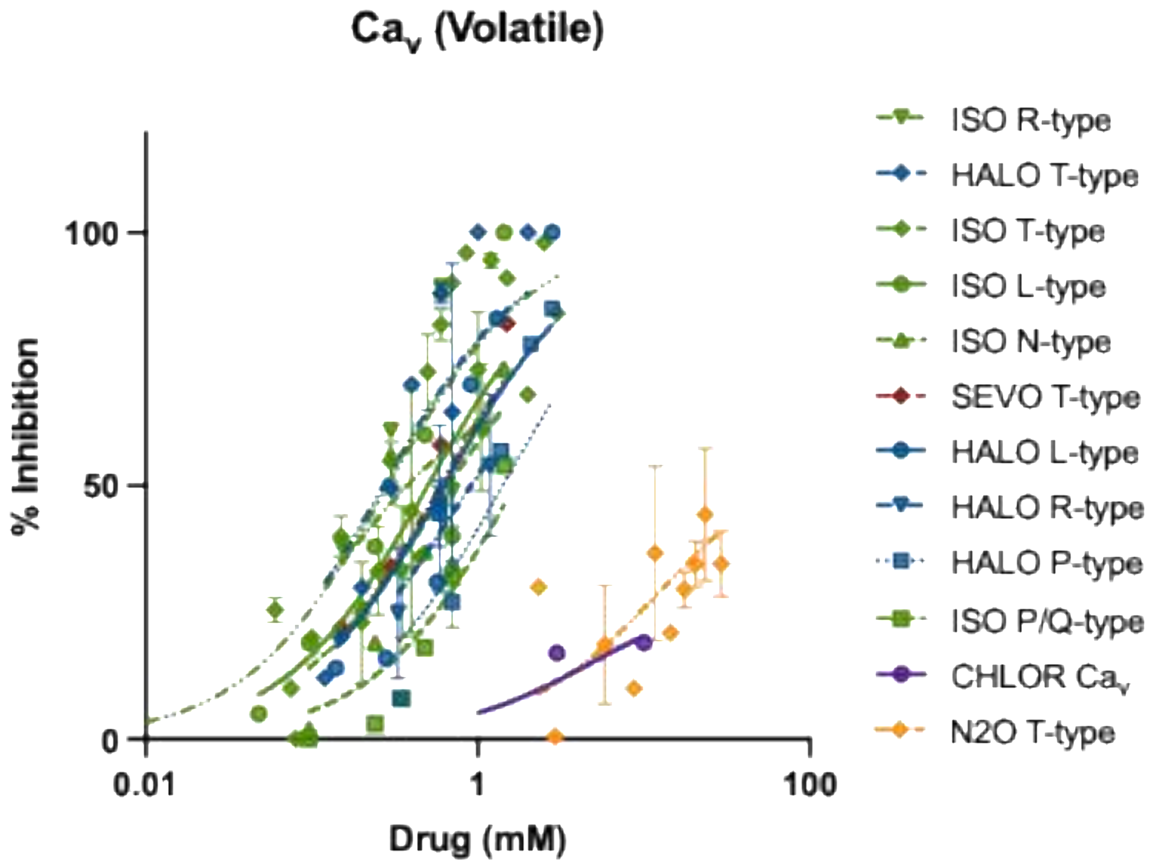

Figure S36

Drug	Subtype	IC_{50} mM (95% CI)	E_{max}	Goodness of Fit		n	
				df	ss	values	studies
ISO	R-type[186,187]	0.159	71.2%	7	3155	9	2
	T-type[59–61,161,311–313]	0.287 (0.198-0.368)	100%	38	10275	40	7
	L-type[186,314,315]	0.499 (0.041-1.18)	100%	5	1583	7	3
	N-type[314,186,316,317]	0.513 (0.004-1.40)	88.5%	8	3597	10	4
	P/Q-type[186,308,314,317]	1.68 (0.209-3.26)	100%	6	912	8	4
HALO	T-type[59,160,161]	0.270	100%	10	4067	12	3
	L-type[186,315,318]	0.626 (0.284-1.07)	100%	6	1230	8	3
	R-type[186]	0.664	86.6%	4	1353	6	1
	P/Q-type[186,308]	1.41 (0.109-2.23)	100%	5	1438	7	2
	N-type[186]	44.5% inhibition at 0.59mM HALO				2	1
SEVO	T-type[161]	0.593 (0.023-1.25)	100%	2	170	4	1
CHLOR	Ca$_v$[222]	4.88	30.2%	1	82.9	3	1
N2O	T-type[227,61,228]	13.0 (0.755-53.3)	59.5%	19	6310	21	3

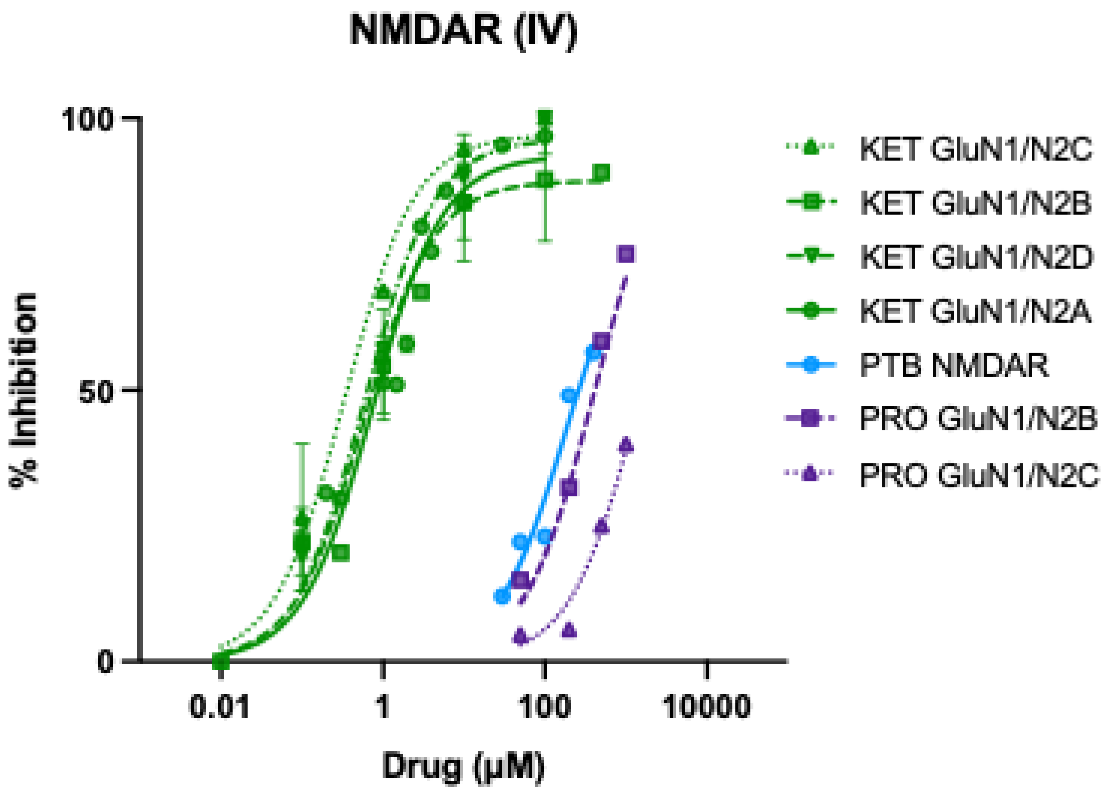

Figure S37

Drug	Subtype	IC_{50} μM (95% CI)	E_{max}	Goodness of Fit		n	
				df	ss	values	studies
KET	GluN1/N2C[106,107]	0.338 (0.132-0.757)	97.0%	5	465	7	2
	GluN1/N2B[107,319,189,106,320]	0.579 (0.219-1.30)	88.5%	19	6158	21	5
	GluN1/N2D[106,107]	0.595 (0.307-1.04)	96.2%	5	208	7	2
	GluN1/N2A[107,319,162,106,321–323,320]	0.743 (0.454-1.15)	93.1%	38	6630	40	8
PTB	NMDAR[81]	186 (49.6-380)	85.4%	3	86.6	5	1
PRO	GluN1/N2B[67]	416 (73.1-652)	100%	2	57.2	4	1
	GluN1/N2C[67]	1580 (121-2977)	100%	2	33.6	4	1

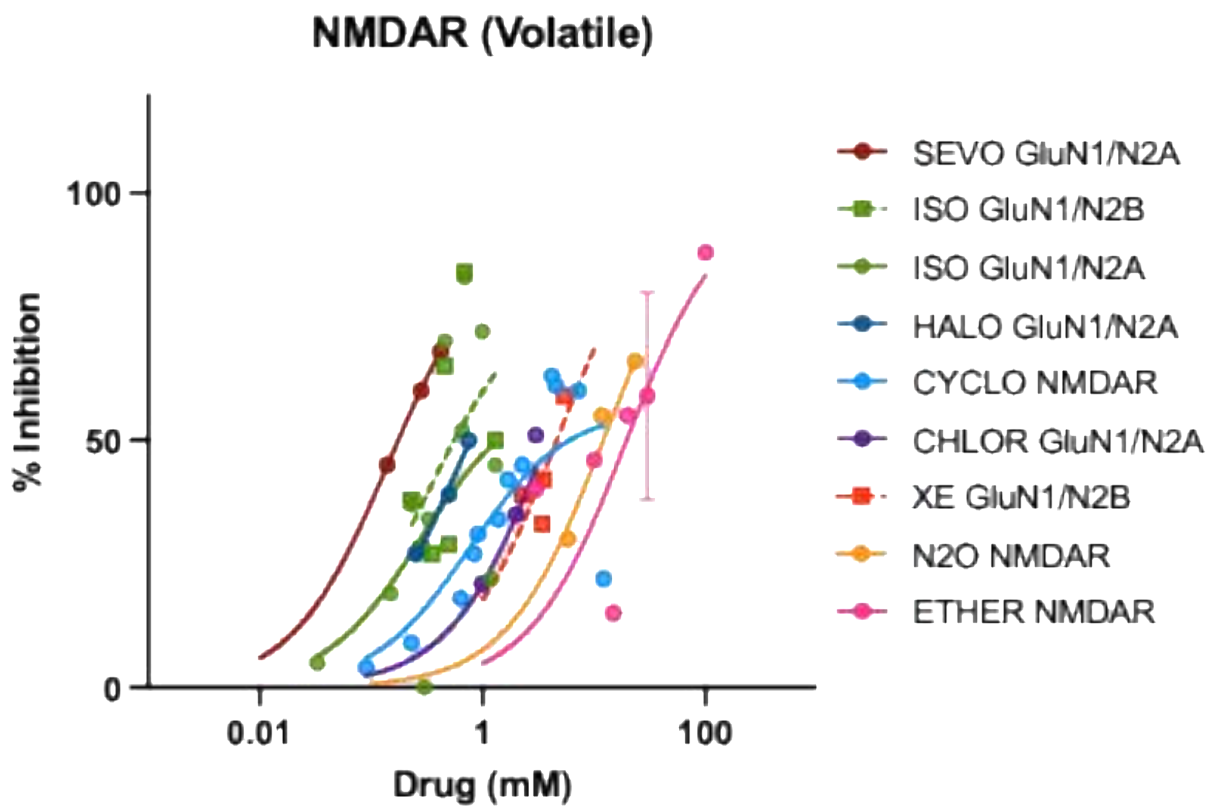

Figure S38

Table over page

Drug	Subtype	IC_{50} mM (95% CI)	E_{max}	Goodness of Fit		n	
				df	ss	values	studies
SEVO	GluN1/N2A[188]	0.144 (0.104-0.192)	91.2%	1	0.06	3	1
	GluN1/N2B[4,188]	43% inhibition at 0.14mM SEVO; 60% at 0.28mM; 14% at 0.34mM; 72% at 0.42mM; 22% at 0.48mM				5	2
ISO	GluN1/N2A[162,184,188–191]	0.282	60.3%	11	6896	13	6
	GluN1/N2B[4,188,189]	0.327	79.4%	5	2094	7	3
HALO	GluN1/N2A[162]	0.594	88.3%	1	3.08	3	1
	GluN1/N2B[4]	17% inhibition at 0.22mM HALO; 26% at 0.35mM HALO				2	1
CYCLO	GluN1/N2A[169,162,191]	0.764 (0.180-2.20)	56.4%	10	1549	12	3
CHLOR	GluN1/N2A[162]	3.77	100%	1	45.0	3	1
XE	GluN1/N2B[4,190,233]	4.64	100%	2	154	4	3
	GluN1/N2A^[162,184,190,191,233,324]	35-43% inhibition between 2mM and 3.5mM XE				6	6
	GluN1/N2D[233]	41.4% inhibition at 3.5mM XE				1	1
	GluN1/N2C[233]	41.3% inhibition at 3.5mM XE				1	1
N2O	NMDAR[226]	12.1	100%	1	41.2	3	1
	GluN1/N2A^[162,184,191]	30-37% inhibition between 12.2mM and 23.4mM N2O				3	3
	GluN1/N2B[189]	28% inhibition at 17.5mM N2O				1	1
ETHER	NMDAR[210,211]	19.4	99.4%	5	2593	7	2

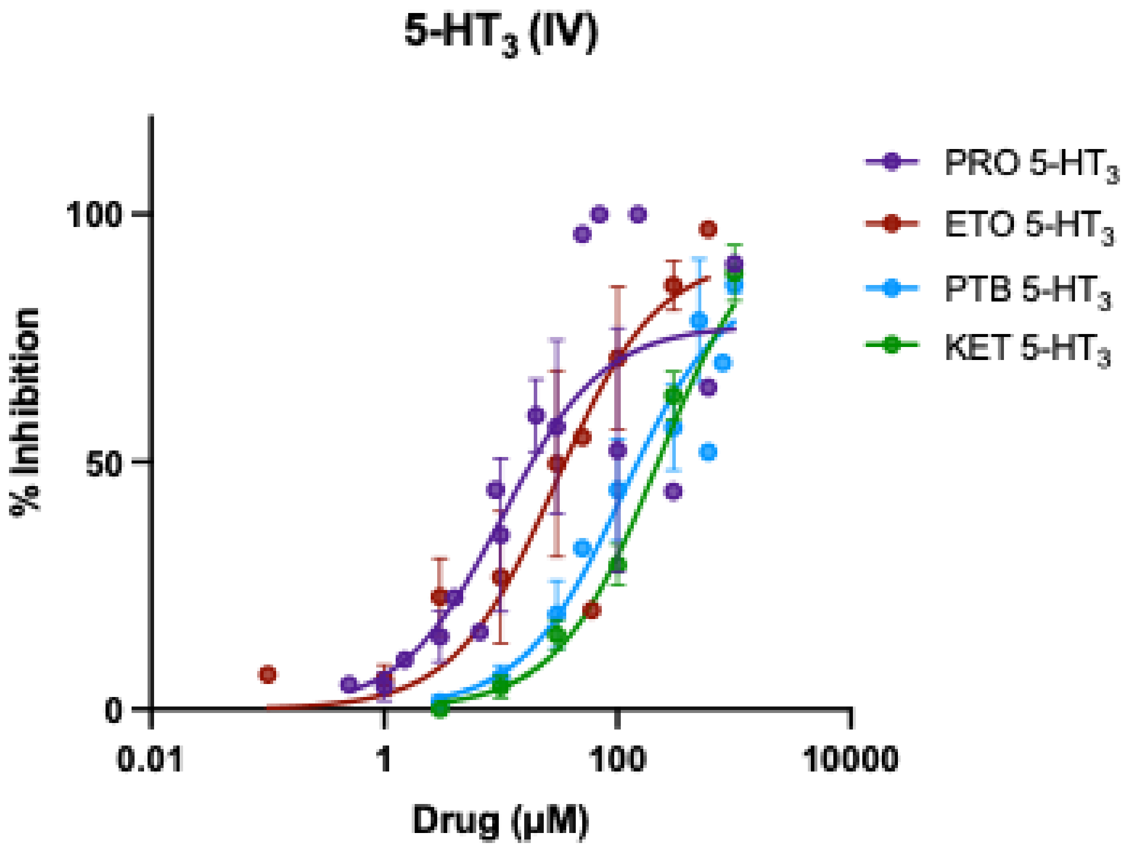

Figure S39A

Drug	Subtype	IC_{50} µM (95% CI)	E_{max}	Goodness of Fit		n	
				df	ss	values	studies
PRO	5-HT_{3A}[46,54–57]	10.2 (3.96-23.8)	77.6%	30	16320	32	5
ETO	5-HT_{3A}[46,56,94,100]	29.5 (6.99-70.3)	91.4%	20	8793	22	4
PTB	5-HT_{3A}[54,56,79,80]	111 (57.0-215)	87.0%	30	6195	32	4
KET	5-HT_3[46,105,325]	223 (129-254)	100%	14	797	16	3

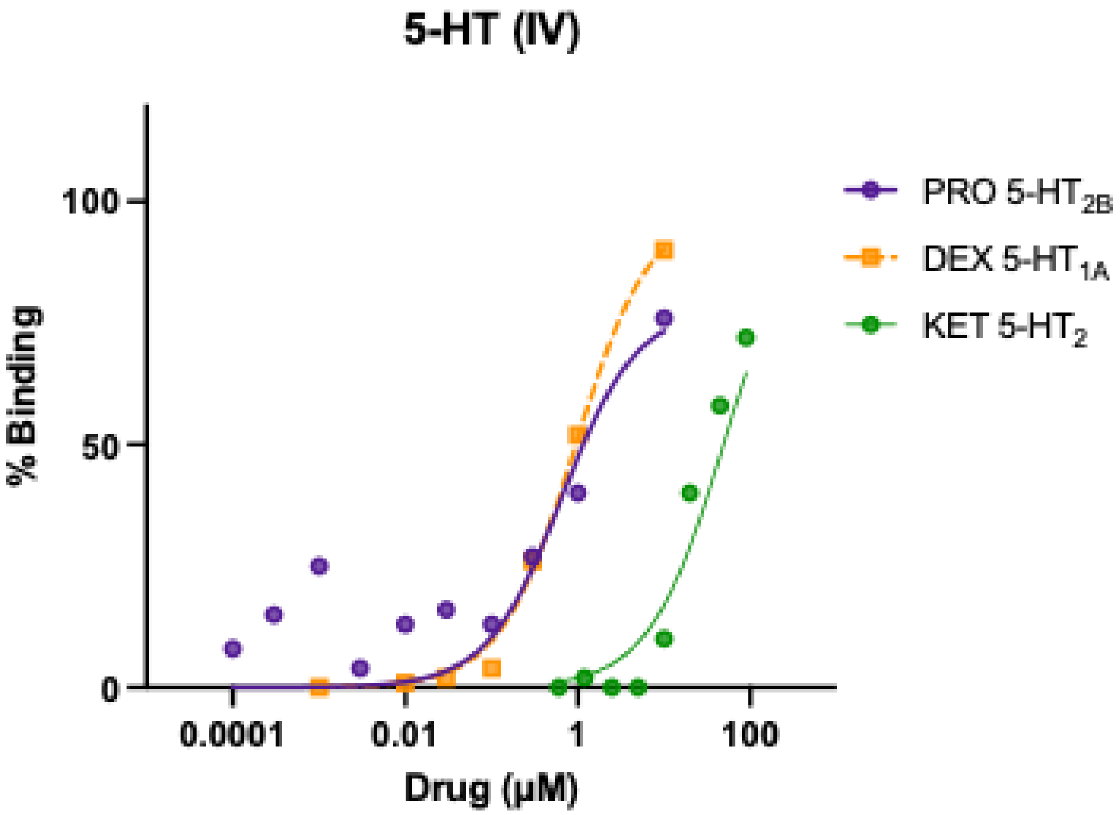

Figure S39B

Drug	Subtype	K_D µM (95% CI)	E_{max}	Goodness of Fit		n	
				df	ss	values	studies
PRO	$5\text{-}HT_{2B}$[326]	0.652 (0.065-3.63)	78.1%	8	1290	10	1
DEX	$5\text{-}HT_{1A}$[124]	0.928 (0.692-1.18)	98.7%	5	36.9	7	1
KET	$5\text{-}HT_2$[113]	48.9 (15.9-66.2)	100%	6	435	8	1

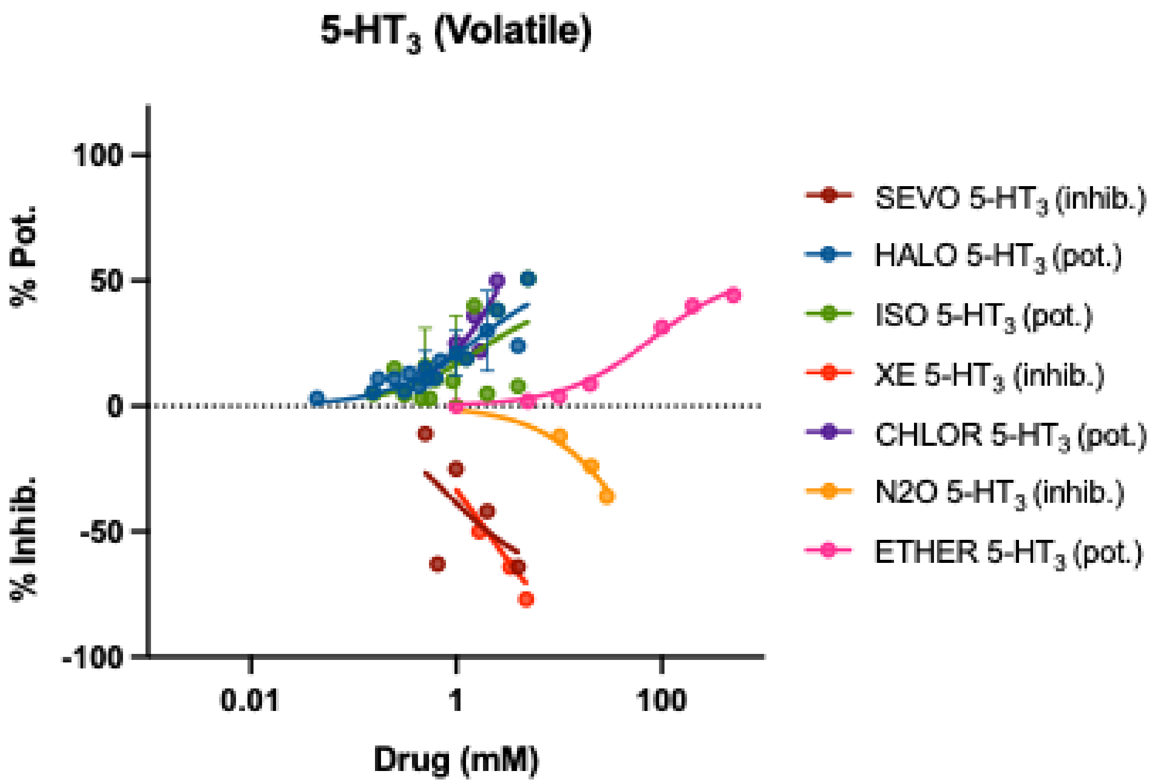

Figure S40

Drug	Subtype	EC_{50}/IC_{50} mM (95% CI)	E_{max}	Goodness of Fit		n	
				df	ss	values	studies
Potentiation							
HALO	5-HT3[172–176]	1.59 (0.536-5.60)	53.5%	18	1245	20	5
ISO	5-HT3[172–176]	1.69	45.0%	18	3073	20	5
CHLOR	5-HT3[175,176]	6.39	164%	3	211	5	2
ETHER	5-HT3[213]	80.0 (52.2-124)	53.4%	5	19.6	7	1
Inhibition							
SEVO	5-HT3[174,176]	0.819	70.2%	3	1525	5	2
XE	5-HT3[174]	1.99	100%	1	59.7	3	1
N2O	5-HT3[174]	59.2	100%	1	19.0	3	1

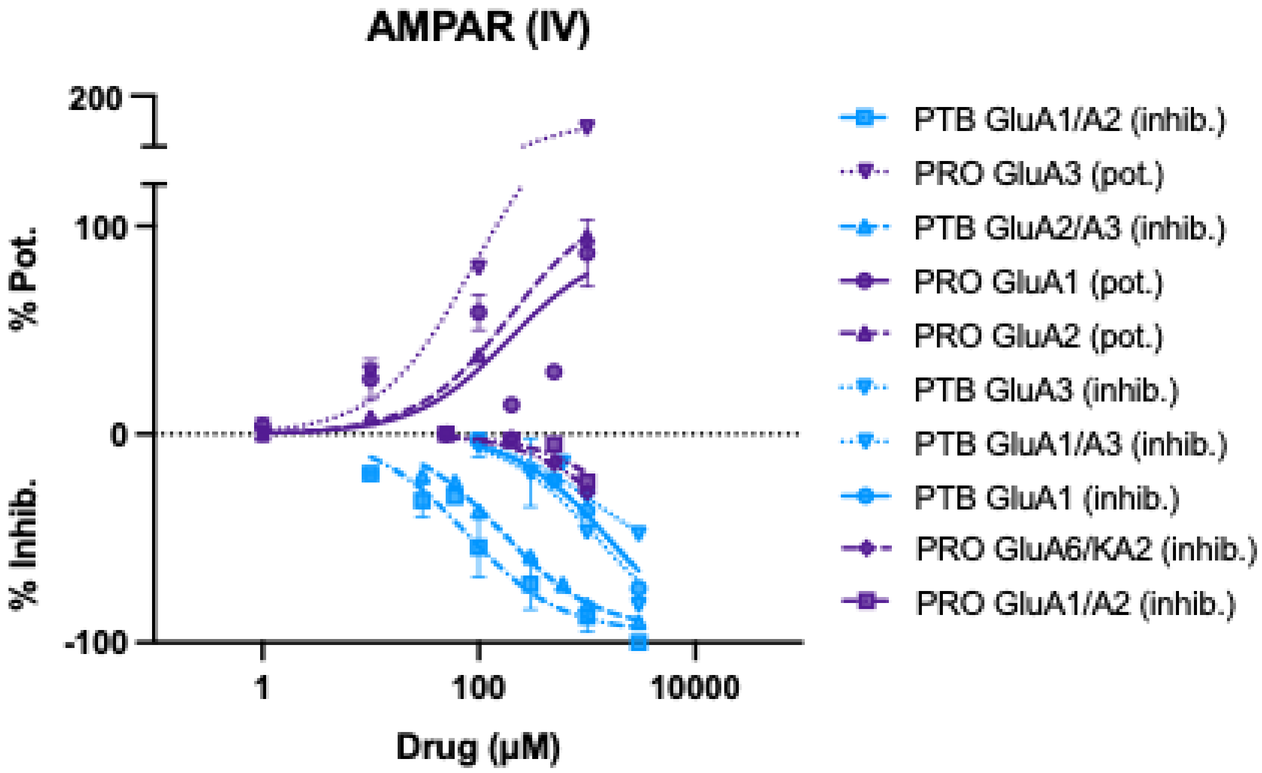

Figure S41

Drug	Subtype	EC_{50}/IC_{50} µM (95% CI)	E_{max}	Goodness of Fit		n	
				df	ss	values	studies
Inhibition							
PTB	GluA1/A2[77,78]	76.4 (35.9-139)	95.0%	10	1356	12	2
	GluA2/A3[77]	162 (122-216)	93.9%	5	45.3	7	1
	GluA3[77,166]	1150 (27.5-6883)	64.9%	5	729	7	2
	GluA1/A3[77]	1240 (106-3364)	100%	2	185	4	1
	GluA1[77]	1572 (286-3134)	100%	2	84.0	4	1
	GluA6[166]	45% inhibition at 100µM PTB; 67% at 300µM PTB				2	1
	GluA5[167]	17% inhibition at 100µM PTB				1	1
PRO	GluR6/KA2[67]	2872 (81.3-6984)	100%	2	28.7	4	1
	GluA1/A2[67]	4241	100%	2	49.7	4	1
Potentiation							
PRO	GluA3[66]	89.3 (12.7-486)	162%	2	223	4	1
	GluA1[66,67]	191.7	91.4%	10	7421	12	2
	GluA2[66]	194 (127-308)	113%	2	6.62	4	11

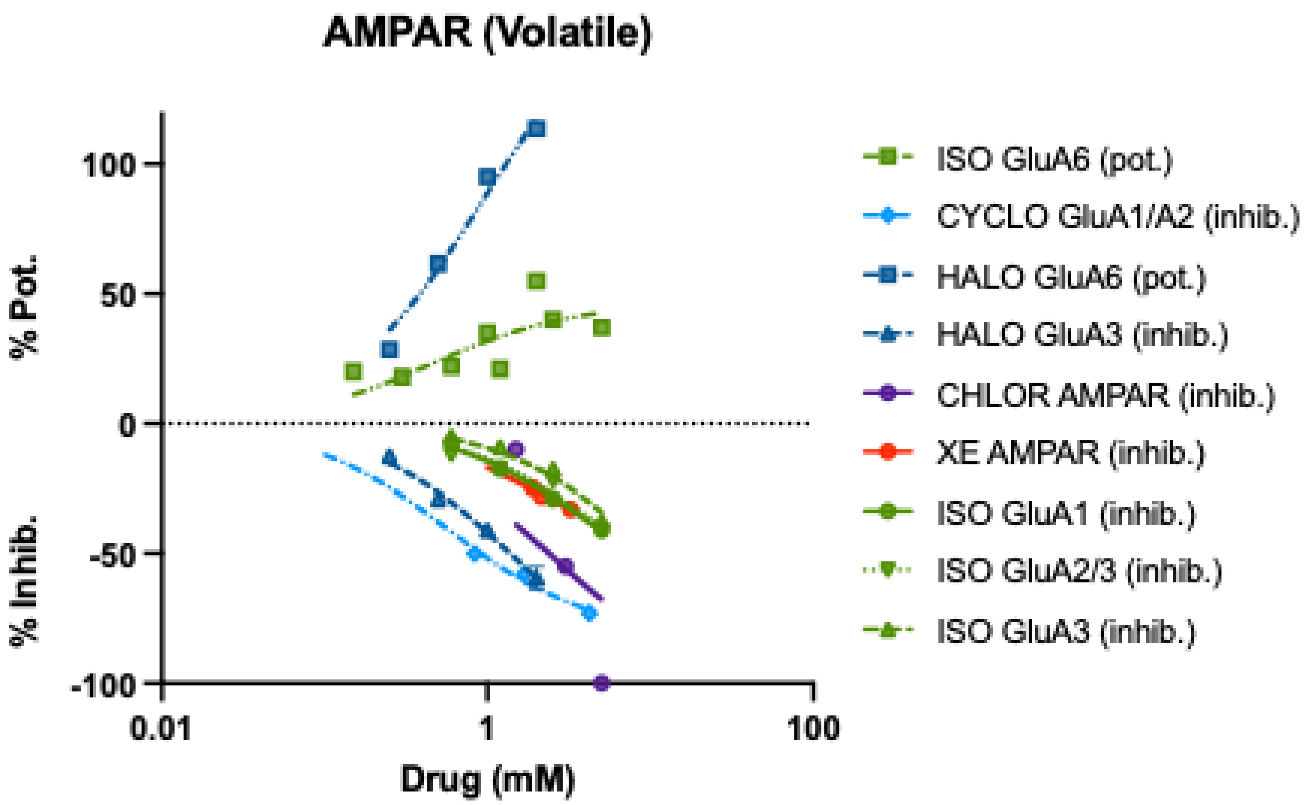

Figure S42

Table over page

Drug	Subtype	EC_{50}/IC_{50} mM (95% CI)	E_{max}	Goodness of Fit		n	
				df	ss	values	studies
Inhibition							
CYCLO	GluA1/A2[169]	0.578	81.6%	1	11.7	3	1
HALO	GluA3[166,167]	1.38 (0.495-1.84)	100%	3	55.6	5	2
CHLOR	AMPAR[210]	2.37	100%	1	1862	3	1
XE	AMPAR[231,232]	2.48	58.7%	1	1.02	3	2
	GluA3[327]	52% inhibition at 3.5mM XE				2	1
	GluA2[324]	49% inhibition at 3.5mM XE				1	1
	GluA4[327]	47% inhibition at 3.5mM XE				2	1
	GluA1[324,327]	43% inhibition at 3.5mM XE				2	1
	GluA1/A2[327]	43% inhibition at 3.5mM XE				1	1
ISO	GluA1[166]	4.32 (1.78-7.92)	77.1%	2	4.84	4	1
	GluA2/A3[166]	4.59 (0.106-13.6)	75.4%	2	38.2	4	1
	GluA3[166]	9.64 (0.997-17.4)	100%	2	25.8	4	1
	GluA1/A2[184]	5% inhibition at 0.15mM ISO				1	1
ETHER	AMPAR[210]	20% inhibition at 15mM ETHER; 57% at 20mM				2	1
N2O	GluA1/A2[184]	20% inhibition at 12.2mM N2O				1	1
Potentiation							
ISO	GluA6[166,167]	0.471 (0.019-2.22)	46.7%	7	657	9	2
	GluA5[167]	170% potentiation at 2mM ISO				1	1
HALO	GluA6[166,167]	0.939 (0.440-2.23)	172%	6	721	8	2
	GluA5[167]	300% potentiation at 2mM HALO				1	1

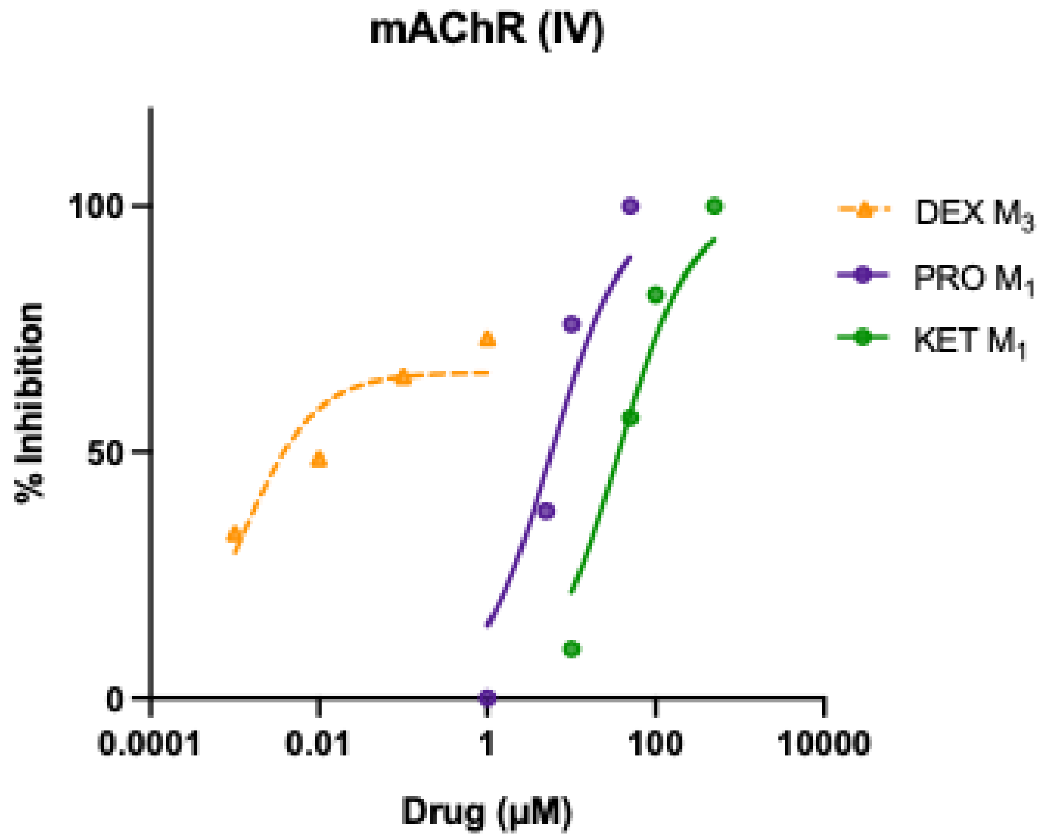

Figure S43

Drug	Subtype	IC_{50} µM (95% CI)	E_{max}	Goodness of Fit		n	
				df	ss	values	studies
DEX	M3[125]	0.001	66.1%	2	170	4	1
PRO	M1[43]	5.80 (0.015-38.7)	100%	2	555	4	1
KET	M1[110]	36.5 (4.55-125)	100%	2	256	4	1

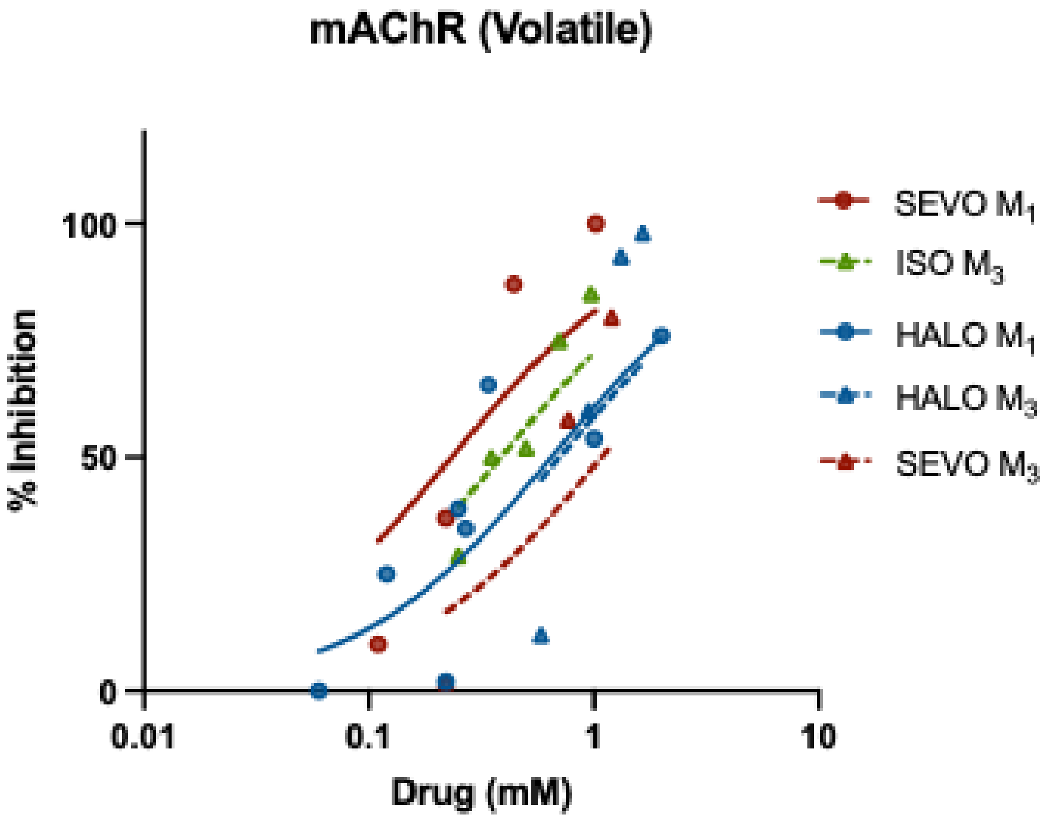

Figure S44

Drug	Subtype	IC_{50} mM (95% CI)	E_{max}	Goodness of Fit		n	
				df	ss	values	studies
SEVO	M1[192]	0.233	100%	2	1434	4	1
	M3[192]	1.08	100%	2	2902	4	1
ISO	M3[192,193]	0.384 (0.009-0.720)	100%	3	424	5	2
	M1[192]	6.30	100%	2	231	4	1
HALO	M1[163,164]	0.649 (0.081-1.67)	100%	7	2766	9	2
	M3[192]	0.704	100%	2	2657	4	1

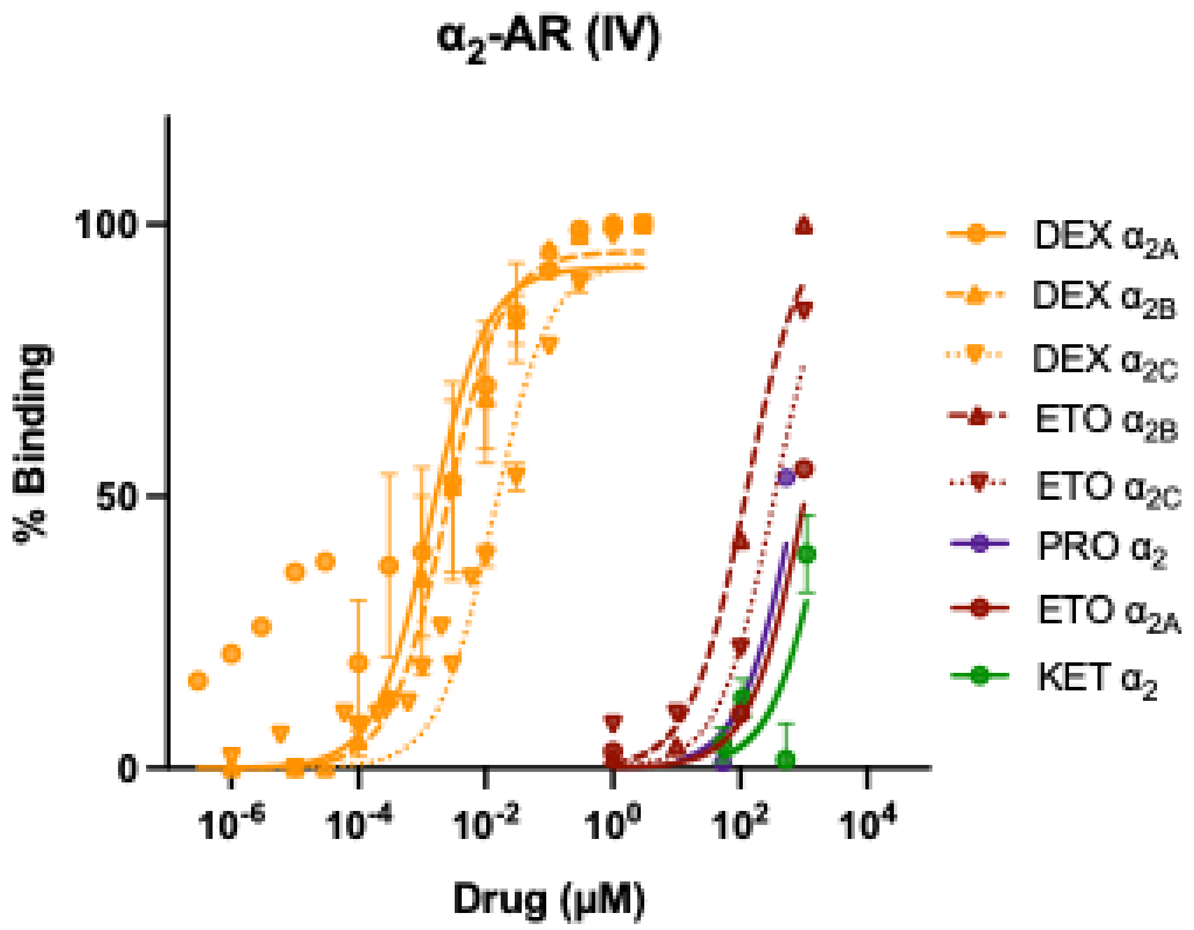

Figure S45

Drug	Subtype	K_D μM (95% CI)	E_{max}	Goodness of Fit		n	
				df	ss	values	studies
DEX	α_{2A}[122–124]	0.0014 (0.0004-0.004)	92.1%	28	12522	30	3
	α_{2B}[122,123]	0.0024 (0.001-0.004)	95.0%	18	1706	20	2
	α_{2C}[122,123]	0.013 (0.008-0.021)	93.1%	21	1383	23	2
ETO	α_{2B}[328]	121 (22.1-432)	100%	2	142	4	1
	α_{2C}[328]	348 (7.50-1372)	100%	2	208	4	1
	α_{2A}[328]	1062 (30.6-2799)	100%	2	88.0	4	1
PRO	α_2[68]	758	100%	2	774	4	1
KET	α_2[68]	2493 (221-4606)	100%	22	5961	24	1

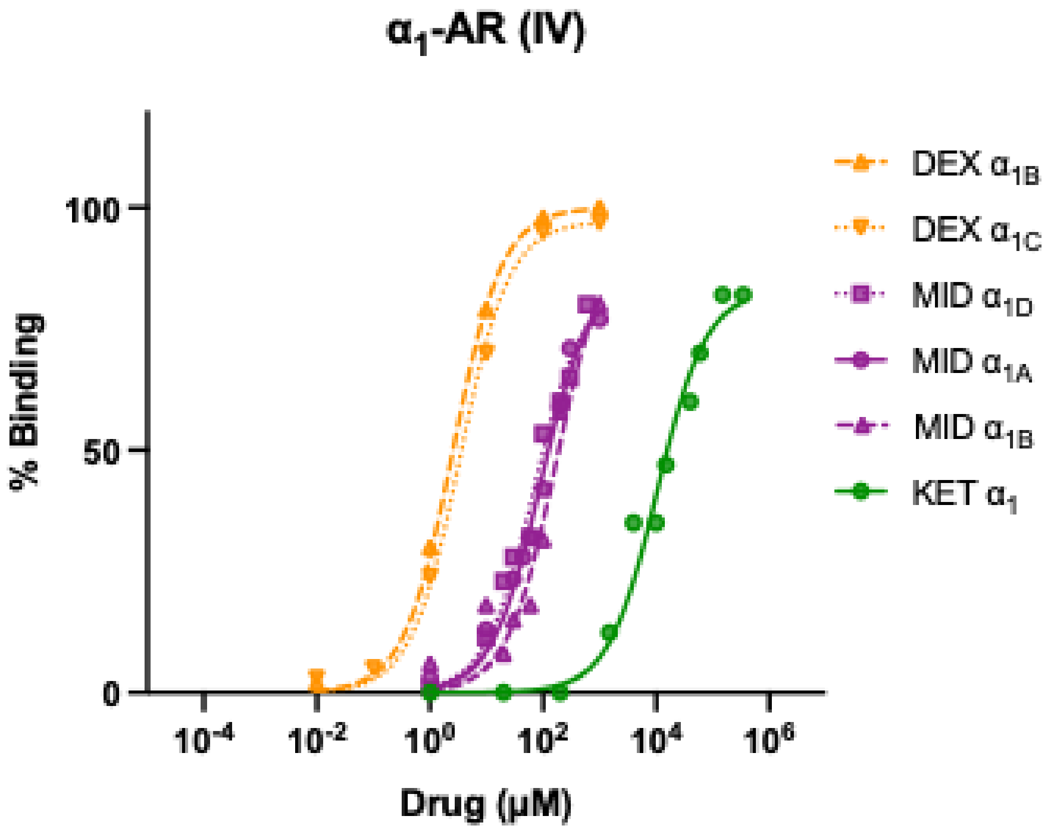

Figure S46

Drug	Subtype	K_D μM (95% CI)	E_{max}	Goodness of Fit		n	
				df	ss	values	studies
DEX	α1B[329]	2.57 (1.49-5.16)	100%	4	83.7	6	1
	α1C[329]	3.41 (2.46-4.70)	97.1%	4	23.4	6	1
MID	α1D[330]	70.8 (45.8-109)	84.7%	7	126	9	1
	α1A[330]	90.5 (67.6-120)	86.1%	7	71.6	9	1
	α1B[330]	172 (83.2-287)	95.5%	6	301	8	1
KET	α1[331]	10442 (6395-16516)	83.0%	9	242	11	1

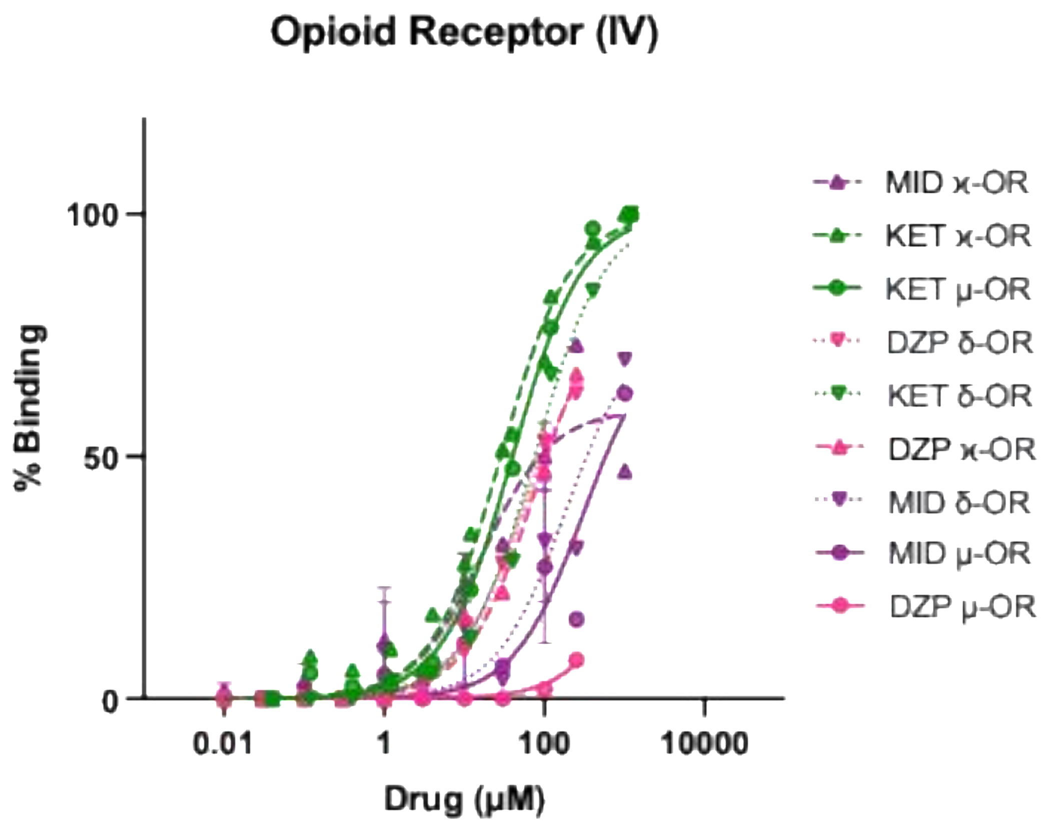

Figure S47

Drug	Subtype	K_D µM (95% CI)	E_{max}	Goodness of Fit		n	
				df	ss	values	studies
MID	κ-OR[139,140]	16.8 (6.01-42.8)	59.6%	14	1133	16	2
	δ-OR[139,140]	184 (30.0-613)	77.4%	14	1732	16	2
	µ-OR[139,140]	304 (33.8-315)	76.3%	14	1412	16	2
KET	κ-OR[108,109]	28.5 (22.7-34.0)	100%	18	344	20	2
	µ-OR[108]	39.8 (32.8-48.4)	100%	8	86.6	10	1
	δ-OR[108]	74.9 (59.8-93.1)	100%	8	111	10	1
DZP	δ-OR[140]	54.0 (44.3-66.0)	78.2%	8	13.8	10	1
	κ-OR[140]	78.1 (47.4-125)	86.6%	8	65.4	10	1
	µ-OR[140]	3140 (631-3788)	100%	8	2.58	10	1

References to data used in tables

1. Zhou J, Noviello CM, Teng J, Moore H, Lega B, Hibbs RE. Resolving native GABAA receptor structures from the human brain. *Nature*. 2025;638(8050):562-568.

2. Hapfelmeier G, Haseneder R, Kochs E, Beyerle M, Zieglgänsberger W. Coadministered nitrous oxide enhances the effect of isoflurane on GABAergic transmission by an increase in open-channel block. *The Journal of Pharmacology and Experimental Therapeutics*. 2001;298(1):201-208.

3. De Sousa SLM, Dickinson R, Lieb WR, Franks NP. Contrasting synaptic actions of the inhalational general anesthetics isoflurane and xenon. *Anesthesiology*. 2000;92(4):1055-1066.

4. Solt K, Eger EI, Raines DE. Differential modulation of human N-methyl-D-aspartate receptors by structurally diverse general anesthetics. *Anesthesia and Analgesia*. 2006;102(5):1407-1411.

5. Smith C, McEwan AI, Jhaveri R, et al. The interaction of fentanyl on the Cp50 of propofol for loss of consciousness and skin incision. *Anesthesiology*. 1994;81(4):820-828.

6. Andrews DT, Leslie K, Sessler DI, Bjorksten AR. The arterial blood propofol concentration preventing movement in 50% of healthy women after skin incision. *Anesthesia and Analgesia*. 1997;85(2):414-419.

7. Irwin MG, Hui TWC, Milne SE, Kenny GNC. Propofol effective concentration 50 and its relationship to bispectral index. *Anaesthesia*. 2002;57(3):242-248.

8. Milne SE, Troy A, Irwin MG, Kenny GNC. Relationship between bispectral index, auditory evoked potential index and effect-site EC50 for propofol at two clinical end-points. *British Journal of Anaesthesia*. 2003;90(2):127-131.

9. Dickinson R, Awaiz S, Whittington MA, Lieb WR, Franks NP. The effects of general anaesthetics on carbachol-evoked gamma oscillations in the rat hippocampus in vitro. *Neuropharmacology*. 2003;44(7):864-872.

10. Servin FS, Sear JW. Pharmacokinetics of intravenous anesthetics. In: Evers AS, Maze M, Kharasch ED, eds. *Anesthetic Pharmacology: Basic Principles and Clinical Practice*. Cambridge University Press; 2011:420-443.

11. Franks NP, Lieb WR. Molecular and cellular mechanisms of general anaesthesia. *Nature*. 1994;367(6464):607-614.

12. Mion G, Villevieille T. Ketamine pharmacology: an update (pharmacodynamics and molecular aspects, recent findings). *CNS Neuroscience & Therapeutics*. 2013;19(6):370-380.

13. Breimer DD. Clinical pharmacokinetics of hypnotics. *Clinical Pharmacokinetics*. 1977;2(2):93-109.

14. Bayliff CD, Schwartz ML, Hardy BG. Pharmacokinetics of high-dose pentobarbital in severe head trauma. *Clinical Pharmacology & Therapeutics*. 1985;38(4):457-461.

15. Ehrnebo M, Odar-Cederlöf I. Binding of amobarbital, pentobarbital and diphenylhydantoin to blood cells and plasma proteins in healthy volunteers and uraemic patients. *European Journal of Clinical Pharmacology*. 1975;8(6):445-453.

16. Franks NP, Lieb WR. Selective actions of volatile general anaesthetics at molecular and cellular levels. *British Journal of Anaesthesia*. 1993;71(1):65-76.

17. Nickalls RWD, Mapleson WW. Age-related iso-MAC charts for isoflurane, sevoflurane and desflurane in man. *British Journal of Anaesthesia*. 2003;91(2):170-174.

18. Ebert TJ, Hall JE, Barney JA, Uhrich TD, Colinco MD. The effects of increasing plasma concentrations of dexmedetomidine in humans. *Anesthesiology*. 2000;93(2):382-394.

19. Cook PJ, Flanagan R, James IM. Diazepam tolerance: effect of age, regular sedation, and alcohol. *British Medical Journal*. 1984;289(6441):351-353.

20. Bremer F, Reulbach U, Schwilden H, Schüttler J. Midazolam therapeutic drug monitoring in intensive care sedation. *Therapeutic Drug Monitoring*. 2004;26(6):643-649.

21. Weerink MAS, Struys MMRF, Hannivoort LN, Barends CRM, Absalom AR, Colin P. Clinical pharmacokinetics and pharmacodynamics of dexmedetomidine. *Clinical Pharmacokinetics*. 2017;56(8):893-913.

22. Dundee JW, Halliday NJ, Harper KW, Brogden RN. Midazolam. *Drugs*. 1984;28(6):519-543.

23. Flood P, Ramirez-Latorre J, Role L. Alpha4beta2 neuronal nicotinic acetylcholine receptors in the central nervous system are inhibited by isoflurane and propofol, but alpha7-type nicotinic acetylcholine receptors are unaffected. *Anesthesiology*. 1997;86(4):859-865.

24. Violet JM, Downie DL, Nakisa RC, Lieb WR, Franks NP. Differential sensitivities of mammalian neuronal and muscle nicotinic acetylcholine receptors to general anesthetics. *Anesthesiology*. 1997;86(4):866-874.

25. Chen X, Shu S, Bayliss DA. Suppression of Ih contributes to propofol-induced inhibition of mouse cortical pyramidal neurons. *Journal of Neurophysiology*. 2005;94(6):3872-3883.

26. Cacheaux LP, Topf N, Tibbs GR, et al. Impairment of hyperpolarization-activated, cyclic nucleotide-gated channel function by the intravenous general anesthetic propofol. *The Journal of Pharmacology and Experimental Therapeutics*. 2005;315(2):517-525.

27. Lyashchenko AK, Redd KJ, Yang J, Tibbs GR. Propofol inhibits HCN1 pacemaker channels by selective association with the closed states of the membrane embedded channel core. *The Journal of Physiology*. 2007;583(1):37-56.

28. Joyce RL, Beyer NP, Vasilopoulos G, et al. Alkylphenol inverse agonists of HCN1 gating: H-bond propensity, ring saturation and adduct geometry differentially determine efficacy and potency. *Biochemical Pharmacology*. 2019;163:493-508.

29. Hill-Venning C, Peters JA, Callachan H, et al. The anaesthetic action and modulation of GABAA receptor activity by the novel water-soluble aminosteroid Org 20599. *Neuropharmacology*. 1996;35(9-10):1209-1222.

30. Hill-Yenning C, Belelli D, Peters JA, Lambert JJ. Subunit-dependent interaction of the general anaesthetic etomidate with the gamma-aminobutyric acid type A receptor. *British Journal of Pharmacology*. 1997;120(5):749-756.

31. Lam DW, Reynolds JN. Modulatory and direct effects of propofol on recombinant GABAA receptors expressed in Xenopus oocytes: influence of α- and γ2-subunits. *Brain Research*. 1998;784(1-2):179-187.

32. Trapani G, Latrofa A, Franco M, et al. Propofol analogues. Synthesis, relationships between structure and affinity at GABAA receptor in rat brain, and differential electrophysiological profile at recombinant human GABAA receptors. *Journal of Medicinal Chemistry*. 1998;41(11):1846-1854.

33. Sanna E, Motzo C, Usala M, et al. Characterization of the electrophysiological and pharmacological effects of 4-iodo-2,6-diisopropylphenol, a propofol analogue devoid of sedative-anaesthetic properties. *British Journal of Pharmacology*. 1999;126(6):1444-1444.

34. Krasowski MD, Jenkins A, Flood P, Kung AY, Hopfinger AJ, Harrison NL. General anesthetic potencies of a series of propofol analogs correlate with potency for potentiation of gamma-aminobutyric acid (GABA) current at the GABAA receptor but not with lipid solubility. *Journal of Pharmacology and Experimental Therapeutics*. 2001;297(1).

35. Krasowski MD, Nishikawa K, Nikolaeva N, Lin A, Harrison NL. Methionine 286 in transmembrane domain 3 of the GABAA receptor β subunit controls a binding cavity for propofol and other alkylphenol general anesthetics. *Neuropharmacology*. 2001;41(8):952-964.

36. Lingamaneni R, Krasowski MD, Jenkins A, et al. Anesthetic properties of 4-iodopropofol: implications for mechanisms of anesthesia. *Anesthesiology*. 2001;94(6):1050-1057.

37. Chang C sheng S, Olcese R, Olsen RW. A single M1 residue in the β2 subunit alters channel gating of GABAA receptor in anesthetic modulation and direct activation. *The Journal of Biological Chemistry*. 2003;278(44):42821-42828.

38. Richardson JE, Garcia PS, O'Toole KK, Derry JMC, Bell SV, Jenkins A. A conserved tyrosine in the β2 subunit M4 segment is a determinant of gamma-aminobutyric acid type A receptor sensitivity to propofol. *Anesthesiology*. 2007;107(3):412-418.

39. Watt EE, Betts BA, Kotey FO, et al. Menthol shares general anesthetic activity and sites of action on the GABAA receptor with the intravenous agent, propofol. *European Journal of Pharmacology*. 2008;590(1):120-126.

40. Hall AC, Griffith TN, Tsikolia M, et al. Cyclohexanol analogues are positive modulators of GABAA receptor currents and act as general anaesthetics in vivo. *European Journal of Pharmacology*. 2011;667(1-3):175-181.

41. Paola Mascia M, Fabbri D, Antonietta Dettori M, Ledda G, Delogu G, Biggio G. Hydroxylated biphenyl derivatives are positive modulators of human GABAA receptors. *European Journal of Pharmacology*. 2012;693(1-3):45-50.

42. Shin DJ, Germann AL, Steinbach JH, Akk G. The actions of drug combinations on the GABAA receptor manifest as curvilinear isoboles of additivity. *Molecular Pharmacology*. 2017;92(5):556-563.

43. Nagase Y, Kaibara M, Uezono Y, Izumi F, Sumikawa K, Taniyama K. Propofol inhibits muscarinic acetylcholine receptor-mediated signal transduction in Xenopus oocytes expressing the rat M1 receptor. *Japanese Journal of Pharmacology*. 1999;79(3):319-325.

44. McGivern J, Scholfield CN. General anaesthetics and field currents in unclamped, unmyelinated axons of rat olfactory cortex. *British Journal of Pharmacology*. 1990;101(1):217-223.

45. Frenkel C, Urban BW. Human brain sodium channels as one of the molecular target sites for the new intravenous anaesthetic propofol (2,6-diisopropylphenol). *European Journal of Pharmacology: Molecular Pharmacology*. 1991;208(1):75-79.

46. Barann M, Göthert M, Fink K, Bönisch H. Inhibition by anaesthetics of 14C-guanidinium flux through the voltage-gated sodium channel and the cation channel of the 5-HT3 receptor of N1E-115 neuroblastoma cells. *Naunyn-Schmiedeberg's Archives of Pharmacology*. 1993;347(2):125-132.

47. Minami K, Yanagihara N, Segawa K, Tsutsui M, Shigematsu A, Izumi F. Inhibitory effects of propofol on catecholamine secretion and uptake in cultured bovine adrenal medullary cells. *Naunyn-Schmiedeberg's Archives of Pharmacology*. 1996;353(5):572-578.

48. Ratnakumari L, Hemmings HC. Inhibition by propofol of [3H]-batrachotoxinin-A 20-alpha-benzoate binding to voltage-dependent sodium channels in rat cortical synaptosomes. *British Journal of Pharmacology*. 1996;119(7):1498-1498.

49. Ratnakumari L, Hammings HC. Effects of propofol on sodium channel-dependent sodium influx and glutamate release in rat cerebrocortical synaptosomes. *Anesthesiology*. 1997;86(2):428-439.

50. Rehberg B, Duch DS. Suppression of central nervous system sodium channels by propofol. *Anesthesiology*. 1999;91(2):512-520.

51. Ouyang W, Wang G, Hemmings HC. Isoflurane and propofol inhibit voltage-gated sodium channels in isolated rat neurohypophysial nerve terminals. *Molecular Pharmacology*. 2003;64(2):373-381.

52. Jones PJ, Wang Y, Smith MD, et al. Hydroxyamide analogs of propofol exhibit state-dependent block of sodium channels in hippocampal neurons: implications for anticonvulsant activity. *The Journal of Pharmacology and Experimental Therapeutics*. 2007;320(2):828-836.

53. Shi QQ, Sun X, Fang H. A mechanism study on propofol's action on middle latency auditory evoked potential by neurons in ventral partition of medial geniculate body in rats. *European Review for Medical and Pharmacological Sciences*. 2014;18(13):1859-1868.

54. Barann M, Dilger JP, Bönisch H, Göthert M, Dybek A, Urban BW. Inhibition of 5-HT3 receptors by propofol: equilibrium and kinetic measurements. *Neuropharmacology*. 2000;39(6):1064-1074.

55. Patten D, Foxon GR, Martin KF, Halliwell RF. An electrophysiological study of the effects of propofol on native neuronal ligand-gated ion channels. *Clinical and Experimental Pharmacology and Physiology*. 2001;28(5-6):451-458.

56. Rüsch D, Braun HA, Wulf H, Schuster A, Raines DE. Inhibition of human 5-HT3A and 5-HT3AB receptors by etomidate, propofol and pentobarbital. *European Journal of Pharmacology*. 2007;573(1-3):60-60.

57. Barann M, Linden I, Witten S, Urban BW. Molecular actions of propofol on human 5-HT3A receptors: enhancement as well as inhibition by closely related phenol derivatives. *Anesthesia and Analgesia*. 2008;106(3):846-857.

58. Pistis M, Belelli D, Peters JA, Lambert JJ. The interaction of general anaesthetics with recombinant GABAA and glycine receptors expressed in Xenopus laevis oocytes: a comparative study. *British Journal of Pharmacology*. 1997;122(8):1707-1719.

59. Todorovic SM, Lingle CJ. Pharmacological properties of T-type Ca2+ current in adult rat sensory neurons: effects of anticonvulsant and anesthetic agents. *Journal of Neurophysiology*. 1998;79(1):240-252.

60. Todorovic SM, Perez-Reyes E, Lingle CJ. Anticonvulsants but not general anesthetics have differential blocking effects on different T-type current variants. *Molecular Pharmacology*. 2000;58(1):98-108.

61. Joksovic PM, Brimelow BC, Murbartián J, Perez-Reyes E, Todorovic SM. Contrasting anesthetic sensitivities of T-type Ca2+ channels of reticular thalamic neurons and recombinant Cav3.3 channels. *British Journal of Pharmacology*. 2005;144(1):59-59.

62. Friederich P, Urban BW. The inhibition of human neuronal K+ currents by general anesthetic agents is altered by extracellular K+. *Molecular Brain Research*. 1998;60(2):301-304.

63. Friederich P, Urban BW. Interaction of intravenous anesthetics with human neuronal potassium currents in relation to clinical concentrations. *Anesthesiology*. 1999;91(6):1853-1853.

64. Friederich P, Benzenberg D, Urban BW. Ketamine and propofol differentially inhibit human neuronal K(+) channels. *European Journal of Anaesthesiology*. 2001;18(3):177-183.

65. Song CY, Xi HJ, Yang L, et al. Propofol inhibited the delayed rectifier potassium current (I(k)) via activation of protein kinase C epsilon in rat parietal cortical neurons. *European Journal of Pharmacology*. 2011;653(1-3):16-20.

66. Krampfl K, Cordes AL, Schlesinger F, Wolfes H, Bufler J. Effects of propofol on recombinant AMPA receptor channels. *European Journal of Pharmacology*. 2005;511(1):1-7.

67. Yamakura T, Sakimura K, Shimoji K, Mishina M. Effects of propofol on various AMPA-, kainate- and NMDA-selective glutamate receptor channels expressed in Xenopus oocytes. *Neuroscience Letters*. 1995;188(3):187-190.

68. Scholz J, Tonner PH, Krause T, et al. [Interactions of intravenous anesthetics with cerebral alpha-2-adrenoceptors]. *Anästhesiologie Intensivmedizin Notfallmedizin Schmerztherapie*. 1999;34(10):642-647.

69. Yost CS, Dodson BA. Inhibition of the nicotinic acetylcholine receptor by barbiturates and by procaine: do they act at different sites? *Cellular and Molecular Neurobiology*. 1993;13(2).

70. Gage PW, McKinnon D. Effects of pentobarbitone on acetylcholine-activated channels in mammalian muscle. *British Journal of Pharmacology*. 1985;85(1):229-235.

71. Dilger JP, Boguslavsky R, Barann M, Katz T, Vidal AM. Mechanisms of barbiturate inhibition of acetylcholine receptor channels. *The Journal of General Physiology*. 1997;109(3):401-414.

72. Klaus K, Friedrich S, Reinhardt D, Bufler J. Pentobarbital has curare-like effects on adult-type nicotinic acetylcholine receptor channel currents. *Anesthesia and Analgesia*. 2000;90(4):970-974.

73. Thompson SA, Whiting PJ, Wafford KA. Barbiturate interactions at the human GABAA receptor: dependence on receptor subunit combination. *British Journal of Pharmacology*. 1996;117(3):521-527.

74. Whittemore ER, Yang W, Drewe JA, Woodward RM. Pharmacology of the human gamma-aminobutyric acidA receptor alpha 4 subunit expressed in Xenopus laevis oocytes. *Molecular Pharmacology*. 1996;50(5):1364-1375.

75. Fukami S, Uchida I, Takenoshita M, Mashimo T, Yoshiya I. The effects of a point mutation of the β2 subunit of GABAA receptor on direct and modulatory actions of general anesthetics. *European Journal of Pharmacology*. 1999;368(2-3):269-276.

76. Carlson BX, Engblom AC, Kristiansen U, Schousboe A, Olsen RW. A single glycine residue at the entrance to the first membrane-spanning domain of the γ-aminobutyric acid type A receptor β2 subunit affects allosteric sensitivity to GABA and anesthetics. *Molecular Pharmacology*. 2000;57(3):474-484.

77. Taverna FA, Cameron BR, Hampson DL, Wang LY, MacDonald JF. Sensitivity of AMPA receptors to pentobarbital. *European Journal of Pharmacology*. 1994;267(3):R3-R5.

78. Yamakura T, Sakimura K, Mishina M, Shimoji K. The sensitivity of AMPA-selective glutamate receptor channels to pentobarbital is determined by a single amino acid residue of the α2 subunit. *FEBS Letters*. 1995;374(3):412-414.

79. Barann M, Göthert M, Bönisch H, Dybek A, Urban BW. 5-HT3 receptors in outside-out patches of N1E-115 neuroblastoma cells: basic properties and effects of pentobarbital. *Neuropharmacology*. 1997;36(4-5):655-664.

80. Barann M, Meder W, Dorner Z, et al. Recombinant human 5-HT3A receptors in outside-out patches of HEK 293 cells: basic properties and barbiturate effects. *Naunyn-Schmiedeberg's archives of pharmacology*. 2000;362(3):255-265.

81. Charlesworth P, Richards CD. Anaesthetic modulation of nicotinic ion channel kinetics in bovine chromaffin cells. *British Journal of Pharmacology*. 1995;114(4):909-917.

82. Nakashima YM, Todorovic SM, Pereverzev A, Hescheler J, Schneider T, Lingle CJ. Properties of Ba2+ currents arising from human α1E and α1Eβ3 constructs expressed in HEK293 cells: physiology, pharmacology, and comparison to native T-type Ba2+ currents. *Neuropharmacology*. 1998;37(8):957-972.

83. Frenkel C, Duch DS, Recio-Pinto E, Urban BW. Pentobarbital suppresses human brain sodium channels. *Molecular Brain Research*. 1989;6(2-3):211-216.

84. McGivern J, Scholfield CN. Phorbol ester and lignocaine or pentobarbitone interactions at presynaptic axons. *Neuroreport*. 1992;3(2):139-142.

85. Frenkel C, Duch DS, Urban BW. Effects of i.v. anaesthetics on human brain sodium channels. *British Journal of Anaesthesia*. 1993;71(1):15-24.

86. Rehberg B, Bennett E, Xiao YH, Levinson SR, Duch DS. Voltage- and frequency-dependent pentobarbital suppression of brain and muscle sodium channels expressed in a mammalian cell line. *Molecular Pharmacology*. 1995;48(1):89-97.

87. Rehberg B, Urban BW, Duch DS. The membrane lipid cholesterol modulates anesthetic actions on a human brain ion channel. *Anesthesiology*. 1995;82(3):749-758.

88. Rehberg B, Wittmann M, Urban BW. Sodium channels (from rat, mouse, and man) in neuroblastoma cells and different expression systems have similar sensitivities to pentobarbital. *Neuroscience Letters*. 1999;264(1-3):81-84.

89. Wartenberg HC, Urban BW, Duch DS. Distinct molecular sites of anaesthetic action: pentobarbital block of human brain sodium channels is alleviated by removal of fast inactivation. *British Journal of Anaesthesia*. 1999;82(1):74-80.

90. Wartenberg HC, Wartenberg JP, Urban BW. Human cardiac sodium channels are affected by pentobarbital. *European Journal of Anaesthesiology*. 2001;18(5):306-313.

91. Sanna E, Murgia A, Casula A, Biggio G. Differential subunit dependence of the actions of the general anesthetics alphaxalone and etomidate at gamma-aminobutyric acid type A receptors expressed in Xenopus laevis oocytes. *Molecular Pharmacology*. 1997;51(3):484-490.

92. Rüsch D, Zhong H, Forman SA. Gating allosterism at a single class of etomidate sites on α1β2γ2L GABAA receptors accounts for both direct activation and acronist modulation. *Journal of Biological Chemistry*. 2004;279(20):20982-20992.

93. Mascia MP, Asproni B, Busonero F, et al. Ethyl 2-(4-bromophenyl)-1-(2,4-dichlorophenyl)-1H-4-imidazolecarboxylate is a novel positive modulator of GABAA receptors. *European Journal of Pharmacology*. 2005;516(3):204-211.

94. Husain SS, Nirthanan S, Ruesch D, et al. Synthesis of trifluoromethylaryl diazirine and benzophenone derivatives of etomidate that are potent general anesthetics and effective photolabels for probing sites on ligand-gated ion channels. *Journal of Medicinal Chemistry*. 2006;49(16):4818-4825.

95. Bright DP, Adham SD, Lemaire LCJM, et al. Identification of anesthetic binding sites on human serum albumin using a novel etomidate photolabel. *The Journal of Biological Chemistry*. 2007;282(16):12038-12047.

96. Forman SA, Desai R, Ruesch D. Gamma-amino butyric acid type A receptor mutations at β2N265 alter etomidate efficacy while preserving basal and agonist-dependent activity. *Anesthesiology*. 2009;111(4):774-774.

97. Guitchounts G, Stewart DS, Forman SA. Two etomidate sites in α1β2γ2 γ-aminobutyric acid type A receptors contribute equally and noncooperatively to modulation of channel gating. *Anesthesiology*. 2012;116(6):1235-1244.

98. Friederich P, Urban BW. [Etomidate inhibits neuronal potassium channels in humans]. *Der Anaesthesist*. 1997;46(5):434-436.

99. Tan H yu, Sun L na, Wang X liang, Ye T hu. Effect of etomidate on voltage-dependent potassium currents in rat isolated hippocampal pyramidal neurons. *Chinese Medical Journal*. 2010;123(6):702-706.

100. Desai R, Miller KW, Raines DE. The pyrrole etomidate analog carboetomidate potently inhibits human 5-HT3A receptor function: comparisons with etomidate and potential implications for emetogenesis. *Anesthesia and Analgesia*. 2013;116(3):573-579.

101. Flood P, Krasowski MD. Intravenous anesthetics differentially modulate ligand-gated ion channels. *Anesthesiology*. 2000;92(5):1418-1425.

102. Putzke C, Hanley PJ, Schlichthör G, et al. Differential effects of volatile and intravenous anesthetics on the activity of human TASK-1. *American Journal of Physiology - Cell Physiology*. 2007;293(4).

103. Zhang Y, He J ce, Liu X kui, Zhang Y, Wang Y, Yu T. Assessment of the effect of etomidate on voltage-gated sodium channels and action potentials in rat primary sensory cortex pyramidal neurons. *European Journal of Pharmacology*. 2014;736:55-62.

104. Zhang M, Yin HJ, Wang WP, Li J, Wang XL. Over-expressed human TREK-1 inhibits CHO cell proliferation via inhibiting PKA and p38 MAPK pathways and subsequently inducing G1 arrest. *Acta Pharmacologica Sinica*. 2016;37(9):1190-1198.

105. Yamakura T, Chavez-Noriega LE, Harris RA. Subunit-dependent inhibition of human neuronal nicotinic acetylcholine receptors and other ligand-gated ion channels by dissociative anesthetics ketamine and dizocilpine. *Anesthesiology*. 2000;92(4):1144-1153.

106. Kotermanski SE, Johnson JW. Mg2+ imparts NMDA receptor subtype selectivity to the Alzheimer's drug memantine. *Journal of Neuroscience*. 2009;29(9):2774-2779.

107. Yamakura T, Mori H, Masaki H, Shimoji K, Mishina M. Different sensitivities of NMDA receptor channel subtypes to non-competitive antagonists. *Neuroreport*. 1993;4(6):687-690.

108. Hirota K, Okawa H, Appadu BL, Grandy DK, Devi LA, Lambert DG. Stereoselective interaction of ketamine with recombinant mu, kappa, and delta opioid receptors expressed in Chinese hamster ovary cells. *Anesthesiology*. 1999;90(1):174-182.

109. Nemeth CL, Paine TA, Rittiner JE, et al. Role of kappa-opioid receptors in the effects of salvinorin A and ketamine on attention in rats. *Psychopharmacology*. 2010;210(2):263-263.

110. Durieux ME, Nietgen GW. Synergistic inhibition of muscarinic signaling by ketamine stereoisomers and the preservative benzethonium chloride. *Anesthesiology*. 1997;86(6):1326-1333.

111. Chen X, Shu S, Bayliss DA. HCN1 channel subunits are a molecular substrate for hypnotic actions of ketamine. *Journal of Neuroscience*. 2009;29(3):600-609.

112. Xing J, Zhang C, Jiang W, et al. The inhibitory effects of ketamine on human hyperpolarization-activated cyclic nucleotide-gated channels and action potential in rabbit sinoatrial node. *Pharmacology*. 2017;99(5-6):226-235.

113. Kapur S, Seeman P. NMDA receptor antagonists ketamine and PCP have direct effects on the dopamine D2 and serotonin 5-HT2 receptors-implications for models of schizophrenia. *Molecular Psychiatry*. 2002;7(8):837-844.

114. Kulkarni RS, Zorn LJ, Anantharam V, Bayley H, Treistman SN. Inhibitory effects of ketamine and halothane on recombinant potassium channels from mammalian brain. *Anesthesiology*. 1996;84(4):900-909.

115. Frenkel C, Urban BW. Molecular actions of racemic ketamine on human CNS sodium channels. *British Journal of Anaesthesia*. 1992;69:292-297.

116. Zhou ZS, Zhao ZQ. Ketamine blockage of both tetrodotoxin (TTX)-sensitive and TTX-resistant sodium channels of rat dorsal root ganglion neurons. *Brain Research Bulletin*. 2000;52(5):427-433.

117. Reckziegel G, Friederich P, Urban BW. Ketamine effects on human neuronal Na+ channels. *European Journal of Anaesthesiology*. 2002;19(9):634-640.

118. Schnoebel R, Wolff M, Peters SC, et al. Ketamine impairs excitability in superficial dorsal horn neurones by blocking sodium and voltage-gated potassium currents. *British Journal of Pharmacology*. 2005;146(6):826-833.

119. Tanahashi S, Iida H, Oda A, Osawa Y, Uchida M, Dohi S. Effects of ifenprodil on voltage-gated tetrodotoxin-resistant Na+ channels in rat sensory neurons. *European Journal of Anaesthesiology*. 2007;24(9):782-788.

120. Huang MH, Lin KH, Chen SJ, Shen AY, Wu FT, Wu SN. Effects of ketamine and its metabolites on ion currents in differentiated hippocampal H19-7 neuronal cells and in HEK293T cells transfected with α-hslo subunit. *Neurotoxicology*. 2012;33(5):1058-1066.

121. Yin J, Fu B, Wang Y, Yu T. Effects of ketamine on voltage-gated sodium channels in the barrel cortex and the ventral posteromedial nucleus slices of rats. *Neuroreport*. 2019;30(17):1197-1204.

122. Jansson CC, Marjamäki A, Luomala K, Savola JM, Scheinin M, Åkerman KEO. Coupling of human α2-adrenoceptor subtypes to regulation of cAMP production in transfected S115 cells. *European Journal of Pharmacology: Molecular Pharmacology*. 1994;266(2):165-174.

123. Svensson SPS, Bailey TJ, Porter AC, Richman JG, Regan JW. Heterologous expression of the cloned guinea pig α2A, α2B, and α2C adrenoceptor subtypes: radioligand binding and functional coupling to a cAMP-responsive reporter gene. *Biochemical Pharmacology*. 1996;51(3):291-300.

124. Newman-Tancredi A, Nicolas JP, Audinot V, et al. Actions of α2 adrenoceptor ligands at α2A and 5-HT1A receptors: the antagonist, atipamezole, and the agonist, dexmedetomidine, are highly selective for α2A adrenoceptors. *Naunyn-Schmiedeberg's Archives of Pharmacology*. 1998;358(2):197-206.

125. Takizuka A, Minami K, Uezono Y, et al. Dexmedetomidine inhibits muscarinic type 3 receptors expressed in Xenopus oocytes and muscarine-induced intracellular Ca2+ elevation in cultured rat dorsal root ganglia cells. *Naunyn-Schmiedeberg's Archives of Pharmacology*. 2007;375(5):293-301.

126. Yang YC, Meng QT, Pan X, Xia ZY, Chen XD. Dexmedetomidine produced analgesic effect via inhibition of HCN currents. *European Journal of Pharmacology*. 2014;740:560-564.

127. Chen BS, Peng H, Wu SN. Dexmedetomidine, an α2-adrenergic agonist, inhibits neuronal delayed-rectifier potassium current and sodium current. *British Journal of Anaesthesia*. 2009;103(2):244-254.

128. Yang L, Tang J, Dong J, Zheng J. Alpha2-adrenoceptor-independent inhibition of acetylcholine receptor channel and sodium channel by dexmedetomidine in rat superior cervical ganglion neurons. *Neuroscience*. 2015;289:9-18.

129. Oda A, Iida H, Tanahashi S, Osawa Y, Yamaguchi S, Dohi S. Effects of α2-adrenoceptor agonists on tetrodotoxin-resistant Na+ channels in rat dorsal root ganglion neurons. *European Journal of Anaesthesiology*. 2007;24(11):934-941.

130. Maruta T, Nemoto T, Satoh S, et al. Dexmedetomidine and clonidine inhibit the function of Nav1.7 independent of α2-adrenoceptor in adrenal chromaffin cells. *Journal of Anesthesia*. 2011;25(4):549-557.

131. Gu XY, Liu BL, Zang KK, et al. Dexmedetomidine inhibits tetrodotoxin-resistant Nav1.8 sodium channel activity through Gi/o-dependent pathway in rat dorsal root ganglion neurons. *Molecular Brain*. 2015;8(1):1-11.

132. Im ST, Jo YY, Han G, Jo HJ, Kim YH, Park CK. Dexmedetomidine inhibits voltage-gated sodium channels via α2-adrenoceptors in trigeminal ganglion neurons. *Mediators of Inflammation*. 2018;2018.

133. Eom W, Lee JM, Park J, Choi K, Jung SJ, Kim HS. The effects of midazolam and sevoflurane on the GABA(A) receptors with alternatively spliced variants of the γ2 subunit. *Korean Journal of Anesthesiology*. 2011;60(2):109-118.

134. Hong DM, Kim CS, Eom W, et al. Interactions of midazolam and propofol on α1β2γ2L and α1β2γ2S gamma aminobutyric acid type A receptors expressed in human embryonic kidney cells. *Anesthesia and Analgesia*. 2011;112(5):1096-1102.

135. Frenkel C, Urban BW. [Interactions of intravenous anesthetics with human CNS ion channels. Electrophysiologic studies with a new type of voltage clamp technique]. *Der Anaesthesist*. 1994;43(4):229-234.

136. Ishizawa Y, Furuya K, Yamagishi S, Dohi S. Non-GABAergic effects of midazolam, diazepam and flumazenil on voltage-dependent ion currents in NG108-15 cells. *Neuroreport*. 1997;8(11):2635-2638.

137. So EC, Wu KC, Kao FC, Wu SN. Effects of midazolam on ion currents and membrane potential in differentiated motor neuron-like NSC-34 and NG108-15 cells. *European Journal of Pharmacology*. 2014;724(1):152-160.

138. Hertle I, Scheller M, Bufler J, et al. Interaction of midazolam with the nicotinic acetylcholine receptor of mouse myotubes. *Anesthesia and Analgesia*. 1997;85(1):174-181.

139. Rattan AK, McDonald JS, Tejwani GA. Differential effects of intrathecal midazolam on morphine-induced antinociception in the rat: role of spinal opioid receptors. *Anesthesia and Analgesia*. 1991;73(2):124-131.

140. Cox RF, Collins MA. The effects of benzodiazepines on human opioid receptor binding and function. *Anesthesia and Analgesia*. 2001;93(2):354-358.

141. Sigel E, Baur R, Trube G, Möhler H, Malherbe P. The effect of subunit composition of rat brain GABAA receptors on channel function. *Neuron*. 1990;5(5):703-711.

142. Angelotti T, Uhler M, Macdonald R. Assembly of GABAA receptor subunits: analysis of transient single-cell expression utilizing a fluorescent substrate/marker gene technique. *The Journal of Neuroscience*. 1993;13(4):1418-1428.

143. Angelotti TP, Uhler MD, Macdonald RL. Enhancement of recombinant gamma-aminobutyric acid type A receptor currents by chronic activation of cAMP-dependent protein kinase. *Molecular Pharmacology*. 1993;44(6):1202-1210.

144. Buhr A, Baur R, Malherbe P, Sigel E. Point mutations of the alpha 1 beta 2 gamma 2 gamma-aminobutyric acid(A) receptor affecting modulation of the channel by ligands of the benzodiazepine binding site. *Molecular Pharmacology*. 1996;49(6):1080-1084.

145. Belelli D, Lambert JJ, Peters JA, Gee KW, Lan NC. Modulation of human recombinant GABAA receptors by pregnanediols. *Neuropharmacology*. 1996;35(9-10):1223-1231.

146. Sigel E, Baur R, Netzer R, Rundfeldt C. The antiepileptic drug AWD 131–138 stimulates different recombinant isoforms of the GABAA receptor through the benzodiazepine binding site. *Neuroscience Letters*. 1998;245(2):85-88.

147. Sigel E, Baur R. Electrophysiological evidence for the coexistence of alpha1 and alpha6 subunits in a single functional GABA(A) receptor. *Journal of Neurochemistry*. 2000;74(6):2590-2596.

148. Wachtel RE, Wegrzynowicz ES. Kinetics of nicotinic acetylcholine ion channels in the presence of intravenous anaesthetics and induction agents. *British Journal of Pharmacology*. 1992;106(3):623-627.

149. Watabe S, Yoshii M, Ogata N, Tsunoo A, Narahashi T. Differential inhibition of transient and long-lasting calcium channel currents by benzodiazepines in neuroblastoma cells. *Brain Research*. 1993;606(2):244-250.

150. Xiang K, Earl DE, Davis KM, Giovannucci DR, Greenfield LJ, Tietz EI. Chronic benzodiazepine administration potentiates high voltage-activated calcium currents in hippocampal CA1 neurons. *The Journal of Pharmacology and Experimental Therapeutics*. 2008;327(3):872-883.

151. Earl DE, Tietz EI. Inhibition of recombinant L-type voltage-gated calcium channels by positive allosteric modulators of GABAA receptors. *The Journal of Pharmacology and Experimental Therapeutics*. 2011;337(1):301-311.

152. Willow M, Kuenzel EA, Catterall WA. Inhibition of voltage-sensitive sodium channels in neuroblastoma cells and synaptosomes by the anticonvulsant drugs diphenylhydantoin and carbamazepine. *Molecular Pharmacology*. 1984;25(2):228-234.

153. Wakamori M, Kaneda M, Oyama Y, Akaike N. Effects of chlordiazepoxide, chlorpromazine, diazepam, diphenylhydantoin, flunitrazepam and haloperidol on the voltage-dependent sodium current of isolated mammalian brain neurons. *Brain Research*. 1989;494(2):374-378.

154. Backus KH, Pflimlin P, Trube G. Action of diazepam on the voltage-dependent Na+ current. Comparison with the effects of phenytoin, carbamazepine, lidocaine and flumazenil. *Brain Research*. 1991;548(1-2):41-49.

155. Mori T, Zhao X, Zuo Y, et al. Modulation of neuronal nicotinic acetylcholine receptors by halothane in rat cortical neurons. *Molecular Pharmacology*. 2001;59(4):732-743.

156. Yamashita M, Mori T, Nagata K, Yeh JZ, Narahashi T. Isoflurane modulation of neuronal nicotinic acetylcholine receptors expressed in human embryonic kidney cells. *Anesthesiology*. 2005;102(1):76-84.

157. Meadows HJ, Randall AD. Functional characterisation of human TASK-3, an acid-sensitive two-pore domain potassium channel. *Neuropharmacology*. 2001;40(4):551-559.

158. Andres-Enguix I, Caley A, Yustos R, et al. Determinants of the anesthetic sensitivity of two-pore domain acid-sensitive potassium channels: molecular cloning of an anesthetic-activated potassium channel from Lymnaea stagnalis. *Journal of Biological Chemistry*. 2007;282(29):20977-20990.

159. Luethy A, Boghosian JD, Srikantha R, Cotten JF. Halogenated ether, alcohol, and alkane anesthetics activate TASK-3 tandem pore potassium channels likely through a common mechanism. *Molecular Pharmacology*. 2017;91(6):620-629.

160. McDowell TS, Pancrazio JJ, Barrett PQ, Lynch C. Volatile anesthetic sensitivity of T-type calcium currents in various cell types. *Anesthesia and Analgesia*. 1999;88(1):168-173.

161. Joksovic PM, Bayliss DA, Todorovic SM. Different kinetic properties of two T-type Ca2+ currents of rat reticular thalamic neurones and their modulation by enflurane. *The Journal of Physiology*. 2005;566(1):125-142.

162. Ogata J, Shiraishi M, Namba T, Smothers CT, Woodward JJ, Harris RA. Effects of anesthetics on mutant N-methyl-D-aspartate receptors expressed in Xenopus oocytes. *The Journal of Pharmacology and Experimental Therapeutics*. 2006;318(1):434-443.

163. Durieux ME. Halothane inhibits signaling through M1 muscarinic receptors expressed in Xenopus oocytes. *Anesthesiology*. 1995;82(1):174-182.

164. Minami K, Vanderah TW, Minami M, Harris RA. Inhibitory effects of anesthetics and ethanol on muscarinic receptors expressed in Xenopus oocytes. *European Journal of Pharmacology*. 1997;339(2-3):237-244.

165. Shiraishi M, Harris RA. Effects of alcohols and anesthetics on recombinant voltage-gated Na+ channels. *The Journal of Pharmacology and Experimental Therapeutics*. 2004;309(3):987-994.

166. Dildy-Mayfield JE, Eger EI, Harris RA. Anesthetics produce subunit-selective actions on glutamate receptors. *The Journal of Pharmacology and Experimental Therapeutics*. 1996;276(3):1058-1065.

167. Minami K, Wick MJ, Stern-Bach Y, et al. Sites of volatile anesthetic action on kainate (Glutamate receptor 6) receptors. *The Journal of Biological Chemistry*. 1998;273(14):8248-8255.

168. Daniels S, Roberts RJ. Post-synaptic inhibitory mechanisms of anaesthesia; glycine receptors. *Toxicology Letters*. 1998;100-101:71-76.

169. Hara K, Eger EI, Laster MJ, Harris RA. Nonhalogenated alkanes cyclopropane and butane affect neurotransmitter-gated ion channel and G-protein-coupled receptors: differential actions on GABAA and glycine receptors. *Anesthesiology*. 2002;97(6):1512-1520.

170. Jenkins A, Lobo IA, Gong D, et al. General anesthetics have additive actions on three ligand-gated ion channels. *Anesthesia and Analgesia*. 2008;107(2):486-486.

171. Chen X, Sirois JE, Lei Q, Talley EM, Lynch C, Bayliss DA. HCN subunit-specific and cAMP-modulated effects of anesthetics on neuronal pacemaker currents. *Journal of Neuroscience*. 2005;25(24):5803-5814.

172. Machu TK, Adron Harris R. Alcohols and anesthetics enhance the function of 5-hydroxytryptamine3 receptors expressed in Xenopus laevis oocytes. *The Journal of Pharmacology and Experimental Therapeutics*. 1994;271(2).

173. Jenkins A, Franks NP, Lieb WR. Actions of general anaesthetics on 5-HT3 receptors in N1E-115 neuroblastoma cells. *British Journal of Pharmacology*. 1996;117(7):1507-1515.

174. Suzuki T, Koyama H, Sugimoto M, Uchida I, Mashimo T. The diverse actions of volatile and gaseous anesthetics on human-cloned 5-hydroxytryptamine3 receptors expressed in Xenopus oocytes. *Anesthesiology*. 2002;96(3):699-704.

175. Lopreato GF, Banerjee P, Mihic SJ. Amino acids in transmembrane domain two influence anesthetic enhancement of serotonin-3A receptor function. *Molecular Brain Research*. 2003;118(1-2):45-51.

176. Stevens R, Rüsch D, Solt K, Raines DE, Davies PA. Modulation of human 5-hydroxytryptamine type 3AB receptors by volatile anesthetics and n-alcohols. *The Journal of Pharmacology and Experimental Therapeutics*. 2005;314(1):338-345.

177. Friederich P, Benzenberg D, Trellakis S, Urban BW. Interaction of volatile anesthetics with human Kv channels in relation to clinical concentrations. *Anesthesiology*. 2001;95(4):954-958.

178. Scheller M, Forman SA. The γ subunit determines whether anesthetic-induced leftward shift is altered by a mutation at α1S270 in α1β2γ2L GABAA receptors. *Anesthesiology*. 2001;95(1):123-131.

179. Jenkins A, Andreasen A, Trudell JR, Harrison NL. Tryptophan scanning mutagenesis in TM4 of the GABAA receptor α1 subunit: implications for modulation by inhaled anesthetics and ion channel structure. *Neuropharmacology*. 2002;43(4):669-678.

180. Nishikawa K, Jenkins A, Paraskevakis I, Harrison NL. Volatile anesthetic actions on the GABAA receptors: contrasting effects of α1(S270) and β2(N265) point mutations. *Neuropharmacology*. 2002;42(3):337-345.

181. Kash TL, Jenkins A, Harrison NL. Molecular volume determines the activity of the halogenated alkane bromoform at wild-type and mutant GABAA receptors. *Brain Research*. 2003;960(1-2):36-41.

182. Kelly EW, Solt K, Raines DE. Volatile aromatic anesthetics variably impact human gamma-aminobutyric acid type A receptor function. *Anesthesia and Analgesia*. 2007;105(5):1287-1292.

183. Cardoso RA, Yamakura T, Brozowski SJ, Chavez-Noriega LE, Harris RA. Human neuronal nicotinic acetylcholine receptors expressed in Xenopus oocytes predict efficacy of halogenated compounds that disobey the Meyer-Overton rule. *Anesthesiology*. 1999;91(5):1370-1377.

184. Yamakura T, Harris RA. Effects of gaseous anesthetics nitrous oxide and xenon on ligand-gated ion channels: comparison with isoflurane and ethanol. *Anesthesiology*. 2000;93(4):1095-1101.

185. Yamakura T, Borghese C, Harris RA. A transmembrane site determines sensitivity of neuronal nicotinic acetylcholine receptors to general anesthetics. *The Journal of Biological Chemistry*. 2000;275(52):40879-40886.

186. Kamatchi GL, Chan CK, Snutch T, Durieux ME, Lynch C. Volatile anesthetic inhibition of neuronal Ca channel currents expressed in Xenopus oocytes. *Brain Research*. 1999;831(1-2):85-96.

187. Joksovic PM, Weiergräber M, Lee WY, Struck H, Schneider T, Todorovic SM. Isoflurane-sensitive presynaptic R-type calcium channels contribute to inhibitory synaptic transmission in the rat thalamus. *Journal of Neuroscience*. 2009;29(5):1434-1445.

188. Hollmann MW, Liu HT, Hoenemann CW, Liu WH, Durieux ME. Modulation of NMDA receptor function by ketamine and magnesium. Part II: interactions with volatile anesthetics. *Anesthesia and Analgesia*. 2001;92(5):1182-1191.

189. Yamakura T, Askalany AR, Petrenko AB, Kohno T, Baba H, Sakimura K. The NR3B subunit does not alter the anesthetic sensitivities of recombinant N-methyl-D-aspartate receptors. *Anesthesia and Analgesia*. 2005;100(6):1687-1692.

190. Dickinson R, Peterson BK, Banks P, et al. Competitive inhibition at the glycine site of the N-methyl-D-aspartate receptor by the anesthetics xenon and isoflurane: evidence from molecular modeling and electrophysiology. *Anesthesiology*. 2007;107(5):756-767.

191. Armstrong SP, Banks PJ, McKitrick TJW, et al. Identification of two mutations (F758W and F758Y) in the N-methyl-D-aspartate receptor glycine-binding site that selectively prevent competitive inhibition by xenon without affecting glycine binding. *Anesthesiology*. 2012;117(1):38-47.

192. Nietgen GW, Hönemann CW, Chan CK, Kamatchi GL, Durieux ME. Volatile anaesthetics have differential effects on recombinant M1 and M3 muscarinic acetylcholine receptor function. *British Journal of Anaesthesia*. 1998;81(4):569-577.

193. Do SH, Kamatchi GL, Durieux ME. The effects of isoflurane on native and chimeric muscarinic acetylcholine receptors: the role of protein kinase C. *Anesthesia and Analgesia*. 2001;93(2):375-381.

194. Topf N, Jenkins A, Baron N, Harrison NL. Effects of isoflurane on gamma-aminobutyric acid type A receptors activated by full and partial agonists. *Anesthesiology*. 2003;98(2):306-311.

195. Nishikawa K, Harrison NL. The actions of sevoflurane and desflurane on the gamma-aminobutyric acid receptor type A: effects of TM2 mutations in the alpha and beta subunits. *Anesthesiology*. 2003;99(3):678-684.

196. Hall AC, Rowan KC, Stevens RJN, Kelley JC, Harrison NL. The effects of isoflurane on desensitized wild-type and α1(S270H) γ-aminobutyric acid type A receptors. *Anesthesia and Analgesia*. 2004;98(5):1297-1304.

197. Jia F, Yue M, Chandra D, Homanics GE, Goldstein PA, Harrison NL. Isoflurane is a potent modulator of extrasynaptic GABA(A) receptors in the thalamus. *The Journal of Pharmacology and Experimental Therapeutics*. 2008;324(3):1127-1135.

198. Yang L, Sonner JM. The anesthetic-like effects of diverse compounds on wild-type and mutant gamma-aminobutyric acid type A and glycine receptors. *Anesthesia and Analgesia*. 2008;106(3):838-845.

199. Downie DL, Hall AC, Lieb WR, Franks NP. Effects of inhalational general anaesthetics on native glycine receptors in rat medullary neurones and recombinant glycine receptors in Xenopus oocytes. *British Journal of Pharmacology*. 1996;118(3):493-502.

200. Rehberg B, Xiao YH, Duch DS. Central nervous system sodium channels are significantly suppressed at clinical concentrations of volatile anesthetics. *Anesthesiology*. 1996;84(5):1223-1233.

201. Ouyang W, Hemmings HC. Isoform-selective effects of isoflurane on voltage-gated Na+ channels. *Anesthesiology*. 2007;107(1):91-98.

202. Zhou C, Johnson KW, Herold KF, Hemmings HC. Differential inhibition of neuronal sodium channel subtypes by the general anesthetic isoflurane. *The Journal of Pharmacology and Experimental Therapeutics*. 2019;369(2):200-211.

203. Chen X, Shu S, Kennedy DP, Willcox SC, Bayliss DA. Subunit-specific effects of isoflurane on neuronal Ih in HCN1 knockout mice. *Journal of Neurophysiology*. 2009;101(1):129-140.

204. Lioudyno MI, Birch AM, Tanaka BS, et al. Shaker-related potassium channels in the central medial nucleus of the thalamus are important molecular targets for arousal suppression by volatile general anesthetics. *Journal of Neuroscience*. 2013;33(41):16310-16322.

205. Liu C, Au JD, Zou HL, Cotten JF, Yost CS. Potent activation of the human tandem pore domain K channel TRESK with clinical concentrations of volatile anesthetics. *Anesthesia and Analgesia*. 2004;99(6):1715-1722.

206. Krasowski MD, Harrison NL. The actions of ether, alcohol and alkane general anaesthetics on GABAA and glycine receptors and the effects of TM2 and TM3 mutations. *British Journal of Pharmacology*. 2000;129(4):731-743.

207. Sugasawa Y, Fukuda M, Ando N, et al. Modulation of hyperpolarization-activated cation current *I*h by volatile anesthetic sevoflurane in the mouse striatum during postnatal development. *Neuroscience Research*. 2018;132:8-16.

208. Schwerin S, Kopp C, Pircher E, et al. Attenuation of native hyperpolarization-activated, cyclic nucleotide-gated channel function by the volatile anesthetic sevoflurane in mouse thalamocortical relay neurons. *Frontiers in Cellular Neuroscience*. 2021;14:606687-606687.

209. Dilger JP, Vidal AM, Mody HI, Liu Y. Evidence for direct actions of general anesthetics on an ion channel protein. A new look at a unified mechanism of action. *Anesthesiology*. 1994;81(2):431-442.

210. Carlà V, Moroni F. General anaesthetics inhibit the responses induced by glutamate receptor agonists in the mouse cortex. *Neuroscience Letters*. 1992;146(1):21-24.

211. Daniell LC. Effect of volatile general anesthetics and n-alcohols on glutamate-stimulated increases in calcium ion flux in hippocampal membrane vesicles. *Pharmacology*. 1995;50(3):154-161.

212. Harris RA, Bruno P. Effects of ethanol and other intoxicant-anesthetics on voltage-dependent sodium channels of brain synaptosomes. *The Journal of Pharmacology and Experimental Therapeutics*. 1985;232(2).

213. Zhou Q, Lovinger DM. Pharmacologic characteristics of potentiation of 5-HT3 receptors by alcohols and diethyl ether in NCB-20 neuroblastoma cells. *The Journal of Pharmacology and Experimental Therapeutics*. 1996;278(2):732-740.

214. Patel AJ, Honoré E, Lesage F, Fink M, Romey G, Lazdunski M. Inhalational anesthetics activate two-pore-domain background K+ channels. *Nature Neuroscience*. 1999;2(5):422-426.

215. Beckstead MJ, Phelan R, Mihic SJ. Antagonism of inhalant and volatile anesthetic enhancement of glycine receptor function. *The Journal of Biological Chemistry*. 2001;276(27):24959-24964.

216. Roberts MT, Phelan R, Erlichman BS, et al. Occupancy of a single anesthetic binding pocket is sufficient to enhance glycine receptor function. *The Journal of Biological Chemistry*. 2006;281(6):3305-3311.

217. Lobo IA, Trudell JR, Harris RA. Accessibility to residues in transmembrane segment four of the glycine receptor. *Neuropharmacology*. 2006;50(2):174-181.

218. Patel AJ, Honoré E, Maingret F, et al. A mammalian two pore domain mechano-gated S-like K+ channel. *The EMBO Journal*. 1998;17(15):4283-4290.

219. Pavel MA, Petersen EN, Wang H, Lerner RA, Hansen SB. Studies on the mechanism of general anesthesia. *Proceedings of the National Academy of Sciences*. 2020;117(24):13757-13766.

220. Zhou Y, Wu HJ, Zhang YH, Sun HY, Wong TM, Li GR. Ionic mechanisms underlying cardiac toxicity of the organochloride solvent trichloromethane. *Toxicology*. 2011;290(2-3):295-304.

221. Liang Q, Anderson WD, Jones ST, et al. Positive allosteric modulation of Kv channels by sevoflurane: insights into the structural basis of inhaled anesthetic action. *PLoS One*. 2015;10(11):e0143363.

222. Harris RA, Bruno P. Membrane disordering by anesthetic drugs: relationship to synaptosomal sodium and calcium fluxes. *Journal of Neurochemistry*. 1985;44(4):1274-1281.

223. Raines DE, Claycomb RJ, Forman SA. Nonhalogenated anesthetic alkanes and perhalogenated nonimmobilizing alkanes inhibit α4β2 neuronal nicotinic acetylcholine receptors. *Anesthesia and Analgesia*. 2002;95(3):573-577.

224. Gruss M, Bushell TJ, Bright DP, Lieb WR, Mathie A, Franks NP. Two-pore-domain K+ channels are a novel target for the anesthetic gases xenon, nitrous oxide, and cyclopropane. *Molecular Pharmacology*. 2004;65(2):443-452.

225. Raines DE, Claycomb RJ, Forman SA. Modulation of GABA(A) receptor function by nonhalogenated alkane anesthetics: the effects on agonist enhancement, direct activation, and inhibition. *Anesthesia and Analgesia*. 2003;96(1):112-118.

226. Jevtović-Todorović V, Todorović SM, Mennerick S, et al. Nitrous oxide (laughing gas) is an NMDA antagonist, neuroprotectant and neurotoxin. *Nature Medicine*. 1998;4(4):460-463.

227. Todorovic SM, Jevtovic-Todorovic V, Mennerick S, Perez-Reyes E, Zorumski CF. Cav3.2 channel is a molecular substrate for inhibition of T-type calcium currents in rat sensory neurons by nitrous oxide. *Molecular pharmacology*. 2001;60(3):603-610.

228. Bartels P, Behnke K, Michels G, et al. Structural and biophysical determinants of single Cav3.1 and Cav3.2 T-type calcium channel inhibition by N2O. *Cell Calcium*. 2009;46(4):293-302.

229. Suzuki T, Ueta K, Sugimoto M, Uchida I, Mashimo T. Nitrous oxide and xenon inhibit the human (alpha 7)5 nicotinic acetylcholine receptor expressed in Xenopus oocyte. *Anesthesia and Analgesia*. 2003;96(2):443-448.

230. Hapfelmeier G, Zieglgänsberger W, Haseneder R, Schneck H, Kochs E. Nitrous oxide and xenon increase the efficacy of GABA at recombinant mammalian GABA(A) receptors. *Anesthesia and Analgesia*. 2000;91(6):1542-1549.

231. Kratzer S, Mattusch C, Kochs E, Eder M, Haseneder R, Rammes G. Xenon attenuates hippocampal long-term potentiation by diminishing synaptic and extrasynaptic N-methyl-D-aspartate receptor currents. *Anesthesiology*. 2012;116(3):673-682.

232. Yamamoto T, Honda H, Baba H, Kohno T. Effect of xenon on excitatory and inhibitory transmission in rat spinal ventral horn neurons. *Anesthesiology*. 2012;116(5):1025-1034.

233. Weigt HU, Adolph O, Georgieff M, Georgieff EM, Föhr KJ. Evidence that xenon does not produce open channel blockade of the NMDA receptor. *Journal of Neurophysiology*. 2008;99(4):1983-1987.

234. Caley AJ, Gruss M, Franks NP. The effects of hypoxia on the modulation of human TREK-1 potassium channels. *The Journal of Physiology*. 2005;562(Pt 1):205-205.

235. Harris K, Armstrong SP, Campos-Pires R, Kiru L, Franks NP, Dickinson R. Neuroprotection against traumatic brain injury by xenon, but not argon, is mediated by inhibition at the N-methyl-D-aspartate receptor glycine site. *Anesthesiology*. 2013;119(5):1137-1148.

236. Mattusch C, Kratzer S, Buerge M, et al. Impact of hyperpolarization-activated, cyclic nucleotide-gated cation channel type 2 for the xenon-mediated anesthetic effect: evidence from in vitro and in vivo experiments. *Anesthesiology*. 2015;122(5):1047-1059.

237. Thompson SA, Bonnert TP, Whiting PJ, Wafford KA. Functional characteristics of recombinant human GABAA receptors containing the ϵ-subunit. *Toxicology Letters*. 1998;100-101:233-238.

238. Sanna E, Mascia MP, Klein RL, Whiting PJ, Biggio G, Harris RA. Actions of the general anesthetic propofol on recombinant human GABAA receptors: influence of receptor subunits. *The Journal of Pharmacology and Experimental Therapeutics*. 1995;274(1):353-360.

239. Wafford KA, Thompson SA, Thomas D, Sikela J, Wilcox AS, Whiting PJ. Functional characterization of human gamma-aminobutyric acidA receptors containing the alpha 4 subunit. *Molecular Pharmacology*. 1996;50:670-678.

240. Krasowski MD, O'Shea SM, Rick CEM, et al. α subunit isoform influences GABAA receptor modulation by propofol. *Neuropharmacology*. 1997;36(7):941-949.

241. Davies PA, Hoffmann EB, Carlisle HJ, Tyndale RF, Hales TG. The influence of an endogenous β3 subunit on recombinant GABAA receptor assembly and pharmacology in WSS-1 cells and transiently transfected HEK293 cells. *Neuropharmacology*. 2000;39(4):611-620.

242. Feng HJ, Macdonald RL. Multiple actions of propofol on αβγ and αβδ GABAA receptors. *Molecular Pharmacology*. 2004;66(6):1517-1524.

243. Germann AL, Shin DJ, Manion BD, et al. Activation and modulation of recombinant glycine and GABAA receptors by 4-halogenated analogues of propofol. *British Journal of Pharmacology*. 2016;173(21):3110-3120.

244. Moraga-Cid G, Yevenes GE, Schmalzing G, Peoples RW, Aguayo LG. A single phenylalanine residue in the main intracellular loop of α1 γ-aminobutyric acid type A and glycine receptors influences their sensitivity to propofol. *Anesthesiology*. 2011;115(3):464-473.

245. Hollands EC, Dale TJ, Baxter AW, et al. Population patch-clamp electrophysiology analysis of recombinant GABAA α1β3γ2 channels expressed in HEK-293 cells. *Journal of Biomolecular Screening*. 2009;14(7):769-780.

246. Aspinwall LS, Bermudez I, King LA, Wafford KA. The interactions of hexachlorocyclohexane isomers with human γ-Aminobutyric AcidA receptors expressed in Xenopus oocytes. *The Journal of Pharmacology and Experimental Therapeutics*. 1997;282(3):1557-1564.

247. Ziemba AM, Forman SA. Correction for inhibition leads to an allosteric co-agonist model for pentobarbital modulation and activation of α1β3γ2L GABAA receptors. *PLOS ONE*. 2016;11(4):e0154031.

248. Lees G, Edwards MD. Modulation of recombination human γ-aminobutyric acid-A receptors by isoflurane: influence of the delta subunit. *Anesthesiology*. 1998;88(1):206-217.

249. Caraiscos VB, Newell JG, You-Ten KE, et al. Selective enhancement of tonic GABAergic inhibition in murine hippocampal neurons by low concentrations of the volatile anesthetic isoflurane. *The Journal of Neuroscience*. 2004;24(39):8454-8458.

250. Krasowski MD, Koltchine VV, Rick CE, Ye Q, Finn SE, Harrison NL. Propofol and other intravenous anesthetics have sites of action on the γ-aminobutyric acid type A receptor distinct from that for isoflurane. *Molecular Pharmacology*. 1998;53(3):530-538.

251. Jensen ML, Timmermann DB, Johansen TH, Schousboe A, Varming T, Ahring PK. The beta subunit determines the ion selectivity of the GABAA receptor. *The Journal of Biological Chemistry*. 2002;277(44):41438-41447.

252. Belelli D, Callachan H, Hill-Venning C, Peters JA, Lambert JJ. Interaction of positive allosteric modulators with human and Drosophila recombinant GABA receptors expressed in Xenopus laevis oocytes. *British Journal of Pharmacology*. 1996;118(3):563-576.

253. You H, Kozuska JL, Paulsen IM, Dunn SMJ. Benzodiazepine modulation of the rat GABAA receptor α4β3γ2L subtype expressed in Xenopus oocytes. *Neuropharmacology*. 2010;59(6):527-533.

254. Brown N, Kerby J, Bonnert TP, Whiting PJ, Wafford KA. Pharmacological characterization of a novel cell line expressing human α4β3δ GABAA receptors. *British Journal of Pharmacology*. 2002;136(7):965-974.

255. Rahman M, Zhu D, Lindblad C, et al. GABA-site antagonism and pentobarbital actions do not depend on the alpha-subunit type in the recombinant rat GABA receptor. *Acta Physiologica*. 2006;187(4):479-488.

256. Belelli D, Lambert JJ, Peters JA, Wafford K, Whiting PJ. The interaction of the general anesthetic etomidate with the γ-aminobutyric acid type A receptor is influenced by a single amino acid. *Proceedings of the National Academy of Sciences*. 1997;94(20):11031-11036.

257. Pistis M, Belelli D, McGurk K, Peters JA, Lambert JJ. Complementary regulation of anaesthetic activation of human (α6β3γ2L) and Drosophila (RDL) GABA receptors by a single amino acid residue. *The Journal of physiology*. 1999;515 (Pt 1)(Pt 1):3-18.

258. Hevers W, Hadley SH, Lüddens H, Amin J. Ketamine, but not phencyclidine, selectively modulates cerebellar GABAA receptors containing α6 and δ subunits. *Journal of Neuroscience*. 2008;28(20):5383-5393.

259. Belelli D, Pistis M, Peters JA, Lambert JJ. The interaction of general anaesthetics and neurosteroids with GABAA and glycine receptors. *Neurochemistry International*. 1999;34(5):447-452.

260. Lor C, Perouansky M, Pearce RA. Isoflurane potentiation of GABAA receptors Is reduced but not eliminated by the β3(N265M) mutation. *International Journal of Molecular Sciences*. 2020;21(24):9534-9534.

261. Koltchine VV, Finn SE, Jenkins A, Nikolaeva N, Lin A, Harrison NL. Agonist gating and isoflurane potentiation in the human γ-aminobutyric acid type A receptor determined by the volume of a second transmembrane domain residue. *Molecular Pharmacology*. 1999;56(5):1087-1093.

262. Borghese CM, Stórustovu S í, Ebert B, et al. The δ subunit of γ-aminobutyric acid type A receptors does not confer sensitivity to low concentrations of ethanol. *The Journal of Pharmacology and Experimental Therapeutics*. 2006;316(3):1360-1368.

263. Schofield CM, Harrison NL. Transmembrane residues define the action of isoflurane at the GABAA receptor alpha-3 subunit. *Brain Research*. 2005;1032(1-2):30-35.

264. Jenkins A, Greenblatt EP, Faulkner HJ, et al. Evidence for a common binding cavity for three general anesthetics within the GABAA receptor. *The Journal of Neuroscience*. 2001;21(6).

265. Shimizu M, Mi X, Toyoda F, et al. Propofol, an anesthetic agent, inhibits HCN channels through the allosteric modulation of the cAMP-dependent gating mechanism. *Biomolecules*. 2022;12(4):570-570.

266. Ying SW, Abbas SY, Harrison NL, Goldstein PA. Propofol block of Ih contributes to the suppression of neuronal excitability and rhythmic burst firing in thalamocortical neurons. *European Journal of Neuroscience*. 2006;23(2):465-480.

267. Wan X, Mathers DA, Puil E. Pentobarbital modulates intrinsic and GABA-receptor conductances in thalamocortical inhibition. *Neuroscience*. 2003;121(4):947-958.

268. Moaddel R, Abdrakhmanova G, Kozak J, et al. Sub-anesthetic concentrations of (R,S)-ketamine metabolites inhibit acetylcholine-evoked currents in α7 nicotinic acetylcholine receptors. *European Journal of Pharmacology*. 2013;698(1-3):228-234.

269. Coates KM, Flood P. Ketamine and its preservative, benzethonium chloride, both inhibit human recombinant α7 and α4β2 neuronal nicotinic acetylcholine receptors in Xenopus oocytes. *British Journal of Pharmacology*. 2001;134(4):871-871.

270. Ho KK, Flood P. Single amino acid residue in the extracellular portion of transmembrane segment 2 in the nicotinic alpha7 acetylcholine receptor modulates sensitivity to ketamine. *Anesthesiology*. 2004;100(3):657-662.

271. Furuya R, Oka K, Watanabe I, Kamiya Y, Itoh H, Andoh T. The effects of ketamine and propofol on neuronal nicotinic acetylcholine receptors and P2x purinoceptors in PC12 cells. *Anesthesia and Analgesia*. 1999;88(1):174-180.

272. Fagerlund MJ, Krupp J, Dabrowski MA. Propofol and AZD3043 inhibit adult muscle and neuronal nicotinic acetylcholine receptors expressed in Xenopus oocytes. *Pharmaceuticals*. 2016;9(1):8-8.

273. Pierce DW, Pejo E, Raines DE, Forman SA. Carboetomidate inhibits alpha4/beta2 neuronal nicotinic acetylcholine receptors at concentrations affecting animals. *Anesthesia and Analgesia*. 2012;115(1):70-70.

274. Mowrey DD, Liu Q, Bondarenko V, et al. Insights into distinct modulation of α7 and α7β2 nicotinic acetylcholine receptors by the volatile anesthetic. *Journal of Biological Chemistry*. 2013;288(50):35793-35800.

275. Flood P, Coates KM. Sensitivity of the α7 nicotinic acetylcholine receptor to isoflurane may depend on receptor inactivation. *Anesthesia and Analgesia*. 2002;95(1):83-87.

276. Jackson SN, Singhal SK, Woods AS, et al. Volatile anesthetics and endogenous cannabinoid anandamide have additive and independent inhibitory effects on α7-nicotinic acetylcholine receptor-mediated responses in Xenopus oocytes. *European Journal of Pharmacology*. 2008;582(1-3):42-51.

277. Downie DL, Vicente-Agullo F, Campos-Caro A, Bushell TJ, Lieb WR, Franks NP. Determinants of the anesthetic sensitivity of neuronal nicotinic acetylcholine receptors. *Journal of Biological Chemistry*. 2002;277(12):10367-10373.

278. Scheller M, Bufler J, Schneck H, Kochs E, Franke C. Isoflurane and sevoflurane interact with the nicotinic acetylcholine receptor channels in micromolar concentrations. *Anesthesiology*. 1997;86(1):118-127.

279. Paul M, Fokt RM, Kindler CH, Dipp NCJ, Yost CS. Characterization of the interactions between volatile anesthetics and neuromuscular blockers at the muscle nicotinic acetylcholine receptor. *Anesthesia and Analgesia*. 2002;95(2):362-367.

280. Liu L, Li W, Wei K, et al. Synergistic effect of sevoflurane and isoflurane on inhibition of the adult-type muscle nicotinic acetylcholine receptor by rocuronium. *Journal of Anesthesia*. 2013;27(3):351-358.

281. Bufler J, Pichlmeier R, Schneck HJ, Hussman H, Franke C. Block of nicotinic acetylcholine-activated channels of cultured mouse myotubes by isoflurane. *Neuroscience Letters*. 1994;168(1-2):135-138.

282. Wenningmann I, Barann M, Vidal AM, Dilger JP. The effects of isoflurane on acetylcholine receptor channels: 3. Effects of conservative polar-to-nonpolar mutations within the channel pore. *Molecular Pharmacology*. 2001;60(3):584-594.

283. Lopes CMB, Gallagher PG, Buck ME, Butler MH, Goldstein SAN. Proton block and voltage gating are potassium-dependent in the cardiac leak channel Kcnk3. *Journal of Biological Chemistry*. 2000;275(22):16969-16978.

284. Pandit JJ, Huskens N, O'Donohoe PB, Turner PJ, Buckler KJ. Competitive interactions between halothane and isoflurane at the carotid body and TASK channels. *Anesthesiology*. 2020;133(5):1046-1059.

285. Berg AP, Talley EM, Manger JP, Bayliss DA. Motoneurons express heteromeric TWIK-related acid-sensitive K+ (TASK) channels containing TASK-1 (KCNK3) and TASK-3 (KCNK9) subunits. *The Journal of Neuroscience*. 2004;24(30):6693-6702.

286. Lesage F, Terrenoire C, Romey G, Lazdunski M. Human TREK2, a 2P domain mechano-sensitive K+ channel with multiple regulations by polyunsaturated fatty acids, lysophospholipids, and Gs, Gi, and Gq protein-coupled receptors. *Journal of Biological Chemistry*. 2000;275(37):28398-28405.

287. Callejo G, Giblin JP, Gasull X. Modulation of TRESK background K+ channel by membrane stretch. *PLOS ONE*. 2013;8(5):e64471-e64471.

288. O'Shea SM, Becker L, Weiher H, Betz H, Laube B. Propofol restores the function of "hyperekplexic" mutant glycine receptors in Xenopus oocytes and mice. *The Journal of Neuroscience*. 2004;24(9):2322-2327.

289. Ahrens J, Leuwer M, Stachura S, et al. A transmembrane residue influences the interaction of propofol with the strychnine-sensitive glycine α1 and α1β receptor. *Anesthesia and Analgesia*. 2008;107(6):1875-1883.

290. Muñoz B, Mariqueo T, Murath P, et al. Modulatory actions of the glycine receptor β subunit on the positive allosteric modulation of ethanol in α2 containing receptors. *Frontiers in Molecular Neuroscience*. 2021;14:763868.

291. Yang L, Sonner JM. The anesthetic-like effects of diverse compounds on wild-type and mutant γ-aminobutyric acid type A and glycine receptors. *Anesthesia and Analgesia*. 2008;106(3):838-845.

292. McCracken ML, Gorini G, McCracken LM, Dayne Mayfield R, Harris RA, Trudell JR. Inter- and intra-subunit butanol/isoflurane sites of action in the human glycine receptor. *Frontiers in Molecular Neuroscience*. 2016;9:45-45.

293. Frenkel C, Weckbecker K, Wartenberg HC, Duch DS, Urban BW. Blocking effects of the anaesthetic etomidate on human brain sodium channels. *Neuroscience Letters*. 1998;249(2-3):131-134.

294. Haeseler G, Störmer M, Bufler J, et al. Propofol blocks human skeletal muscle sodium channels in a voltage-dependent manner. *Anesthesia and Analgesia*. 2001;92(5):1192-1198.

295. Wagner LE, Gingrich KJ, Kulli JC, Yang J. Ketamine blockade of voltage-gated sodium channels: evidence for a shared receptor site with local anesthetics. *Anesthesiology*. 2001;95(6):1406-1413.

296. Herold KF, Nau C, Ouyang W, Hemmings HC. Isoflurane inhibits the tetrodotoxin-resistant voltage-gated sodium channel Nav1.8. *Anesthesiology*. 2009;111(3):591-599.

297. Yokoyama T, Minami K, Sudo Y, et al. Effects of sevoflurane on voltage-gated sodium channel Nav1.8, Nav1.7, and Nav1.4 expressed in Xenopus oocytes. *Journal of Anesthesia*. 2011;25(4):609-613.

298. Ouyang W, Herold KF, Hemmings HC. Comparative effects of halogenated inhaled anesthetics on voltage-gated Na+ channel function. *Anesthesiology*. 2009;110(3):582-590.

299. Patel MK, Mistry D, John JE, Mounsey JP. Sodium channel isoform-specific effects of halothane: protein kinase C co-expression and slow inactivation gating. *British Journal of Pharmacology*. 2000;130(8):1785-1785.

300. Friederich P, Trellakis S, Urban BW. [Epileptic seizures from etomidate? The human Kv1.1 potassium channel in humans]. *Anasthesiol Intensivmed Notfallmed Schmerzther*. 2001;36(2):100-104.

301. Zhang YZ, Zhang R, Zeng XZ, Song CY. The inhibitory effect of propofol on Kv2.1 potassium channel in rat parietal cortical neurons. *Neuroscience Letters*. 2016;616:93-97.

302. Rothman S. Noncompetitive N-methyl-D-aspartate antagonists affect multiple ionic currents. *The Journal of Pharmacology and Experimental Therapeutics*. 1988;246(1):137-142.

303. Yin J, Fu B, Zhang Y, Yu T. Effect of ketamine on voltage-gated potassium channels in rat primary sensory cortex pyramidal neurons. *Neuroreport*. 2020;31(8):583-589.

304. Friederich P, Dybek A, Urban BW. Stereospecific interaction of ketamine with nicotinic acetylcholine receptors in human sympathetic ganglion-like SH-SY5Y cells. *Anesthesiology*. 2000;93(3):818-824.

305. Lin H, Kim JG, Park SW, et al. Enhancement of 5-HT2A receptor function and blockade of Kv1.5 by MK801 and ketamine: implications for PCP derivative-induced disease models. *Experimental & Molecular Medicine*. 2018;50(4):1-8.

306. Olcese R, Usai C, Maestrone E, Nobile M. The general anesthetic propofol inhibits transmembrane calcium current in chick sensory neurons. *Anesthesia and Analgesia*. 1994;78(5):955-960.

307. Martella G, De Persis C, Bonsi P, et al. Inhibition of persistent sodium current fraction and voltage-gated L-type calcium current by propofol in cortical neurons: implications for its antiepileptic activity. *Epilepsia*. 2005;46(5):624-635.

308. Hall AC, Lieb WR, Franks NP. Insensitivity of P-type calcium channels to inhalational and intravenous general anesthetics. *Anesthesiology*. 1994;81(1):117-123.

309. Schober A, Sokolova E, Gingrich KJ. Pentobarbital inhibition of human recombinant α1A P/Q-type voltage-gated calcium channels involves slow, open channel block. *British Journal of Pharmacology*. 2010;161(2):365-365.

310. Gundersen CB, Umbach JA, Swartz BE. Barbiturates depress currents through human brain calcium channels studied in Xenopus oocytes. *The Journal of Pharmacology and Experimental Therapeutics*. 1988;247(3):824-829.

311. Orestes P, Bojadzic D, Chow RM, Todorovic SM. Mechanisms and functional significance of inhibition of neuronal T-type calcium channels by isoflurane. *Molecular Pharmacology*. 2009;75(3):542-554.

312. Eckle VS, DiGruccio MR, Uebele VN, Renger JJ, Todorovic SM. Inhibition of T-type calcium current in rat thalamocortical neurons by isoflurane. *Neuropharmacology*. 2012;63(2):266-273.

313. Timic Stamenic T, Feseha S, Valdez R, Zhao W, Klawitter J, Todorovic SM. Alterations in oscillatory behavior of central medial thalamic neurons demonstrate a key role of Cav3.1 isoform of T-Channels during isoflurane-induced anesthesia. *Cerebral Cortex*. 2019;29(11):4679-4696.

314. Kameyama K, Aono K, Kitamura K. Isoflurane inhibits neuronal Ca2+ channels through enhancement of current inactivation. *British Journal of Anaesthesia*. 1999;82(3):402-413.

315. Kamatchi GL, Durieux ME, Lynch C. Differential sensitivity of expressed L-type calcium channels and muscarinic M1 receptors to volatile anesthetics in Xenopus oocytes. *The Journal of Pharmacology and Experimental Therapeutics*. 2001;297(3):981-990.

316. Recio-Pinto E, Nikonorov IM, Blanck TJJ. G-protein activation decreases the isoflurane inhibition of N-type Ca2+ currents. An increase in the isoflurane blocking potency of N-type Ca2+ currents may contribute to the known neuroprotection action of isoflurane during ischemia. *Journal of Neurosurgical Anesthesiology*. 2004;16(1):105-107.

317. Rajagopal S, Fang H, Lynch C, Sando JJ, Kamatchi GL. Effects of isoflurane on the expressed Cav2.2 currents in Xenopus oocytes depend on the activation of protein kinase Cδ and its phosphorylation sites in the Cav2.2α1 subunits. *Neuroscience*. 2011;182:232-240.

318. Nikonorov IM, Blanck TJJ, Recio-Pinto E. The effects of halothane on single human neuronal L-type calcium channels. *Anesthesia and Analgesia*. 1998;86(4):885-895.

319. Liu HT, Hollmann MW, Liu WH, Hoenemann CW, Durieux ME. Modulation of NMDA receptor function by ketamine and magnesium: Part I. *Anesthesia and Analgesia*. 2001;92(5):1173-1181.

320. Glasgow NG, Povysheva NV, Azofeifa AM, Johnson JW. Memantine and ketamine differentially alter NMDA receptor desensitization. *The Journal of Neuroscience*. 2017;37(40):9686-9704.

321. Kotermanski SE, Wood JT, Johnson JW. Memantine binding to a superficial site on NMDA receptors contributes to partial trapping. *The Journal of Physiology*. 2009;587(Pt 19):4589-4604.

322. Gilling KE, Jatzke C, Hechenberger M, Parsons CG. Potency, voltage-dependency, agonist concentration-dependency, blocking kinetics and partial untrapping of the uncompetitive N-

methyl-D-aspartate (NMDA) channel blocker memantine at human NMDA (GluN1/GluN2A) receptors. *Neuropharmacology*. 2009;56(5):866-875.

323. Brosnan RJ, Pham TL. Does anesthetic additivity imply a similar molecular mechanism of anesthetic action at N-methyl-D-aspartate receptors? *Anesthesia and Analgesia*. 2011;112(3):568.

324. Weigt HU, Föhr KJ, Georgieff M, Georgieff EM, Senftleben U, Adolph O. Xenon blocks AMPA and NMDA receptor channels by different mechanisms. *Acta Neurobiologiae Experimentalis*. 2009;69(4):429-440.

325. Molderings GJ, Schmidt K, Bönisch H, Göthert M. Inhibition of 5-HT3 receptor function by imidazolines in mouse neuroblastoma cells: potential involvement of sigma 2 binding sites. *Naunyn-Schmiedeberg's Archives of Pharmacology*. 1996;354(3):245-252.

326. Matsunaga F, Gao L, Huang XP, Saven JG, Roth BL, Liu R. Molecular interactions between general anesthetics and the 5HT2B receptor. *Journal of Biomolecular Structure and Dynamics*. 2015;33(1):211-218.

327. Plested AJR, Wildman SS, Lieb WR, Franks NP. Determinants of the sensitivity of AMPA receptors to xenon. *Anesthesiology*. 2004;100(2):347-358.

328. Paris A, Philipp M, Tonner PH, et al. Activation of α2B-adrenoceptors mediates the cardiovascular effects of etomidate. *Anesthesiology*. 2003;99(4):889-895.

329. Schwinn DA, Correa-Sales C, Page SO, Maze M. Functional effects of activation of alpha-1 adrenoceptors by dexmedetomidine: in vivo and in vitro studies. *The Journal of Pharmacology and Experimental Therapeutics*. 1991;259(3):1147-1152.

330. Waugh DJ, Gaivin RJ, Damron DS, Murray PA, Perez DM. Binding, partial agonism, and potentiation of α1-adrenergic receptor function by benzodiazepines: a potential site of allosteric modulation. *The Journal of Pharmacology and Experimental Therapeutics*. 1999;291(3):1164-1171.

331. Bevan RK, Rose MA, Duggan KA. Evidence for direct interaction of ketamine with α1- and β2-adrenoceptors. *Clinical and Experimental Pharmacology and Physiology*. 1997;24(12):923-926.
